# Supplementary figures and images for: Genomic landscape of TP53-mutated myeloid malignancies
Source: medRxiv. 2023 Jan 11:2023.01.10.23284322. Preprint. [Version 1] doi: 10.1101/2023.01.10.23284322 (PMC9882519; doi:10.1101/2023.01.10.23284322)

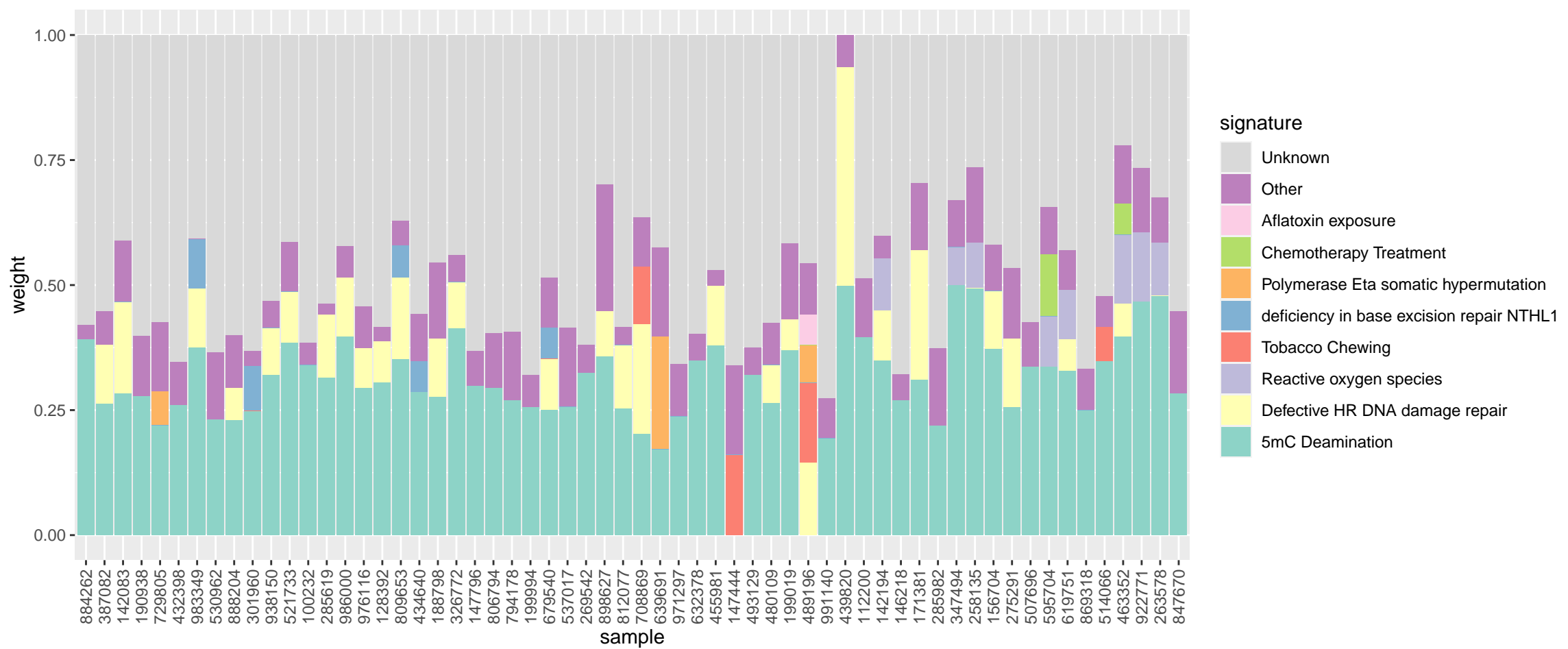

Supplement: Supplement 2 — Supplementary Figure 1. Mutation signatures [file media-2.pdf]

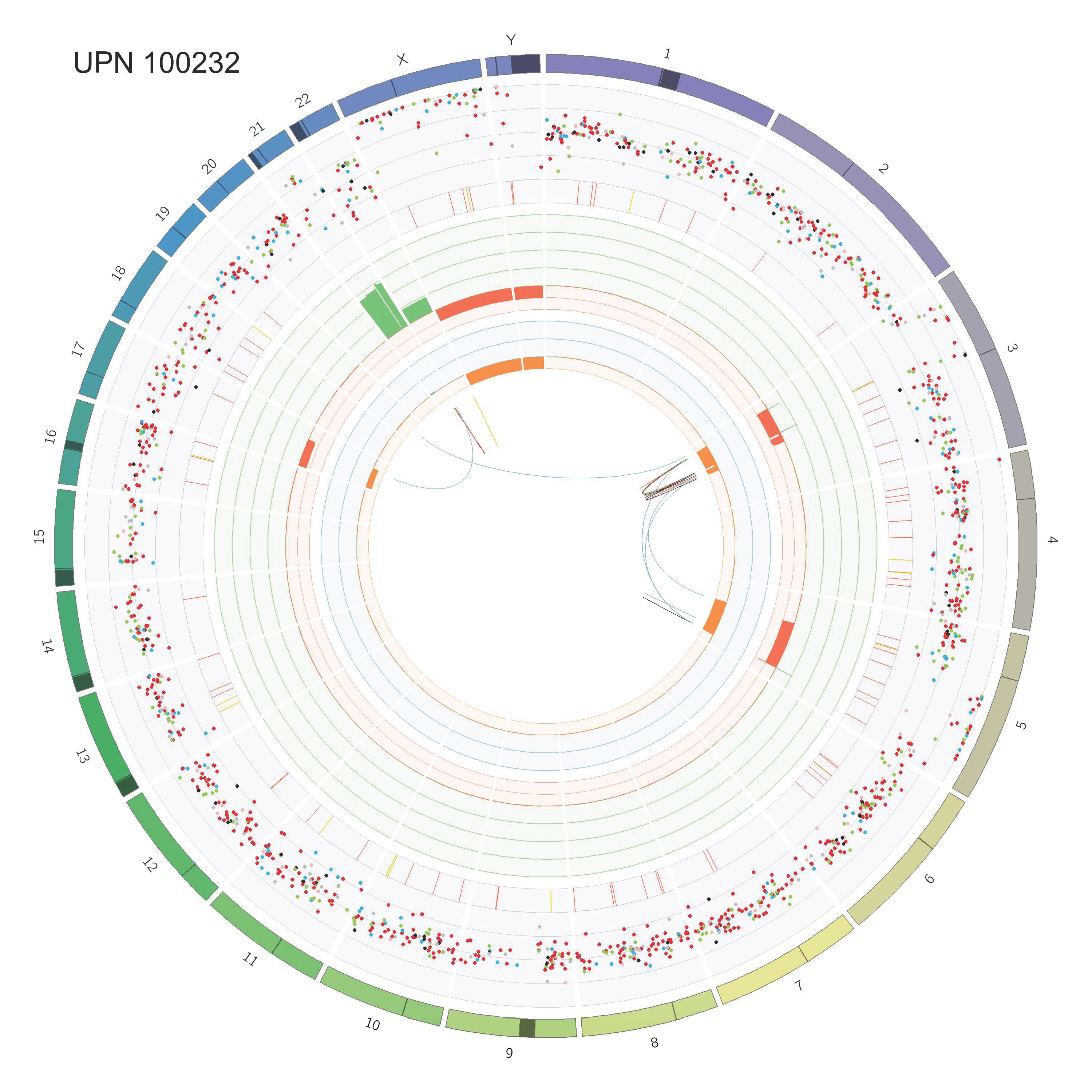

Supplement: Supplement 3 — Supplementary Figure 2. Circos plots [file media-3.zip › Supp_Fig_2_circos_Page_01.jpg]

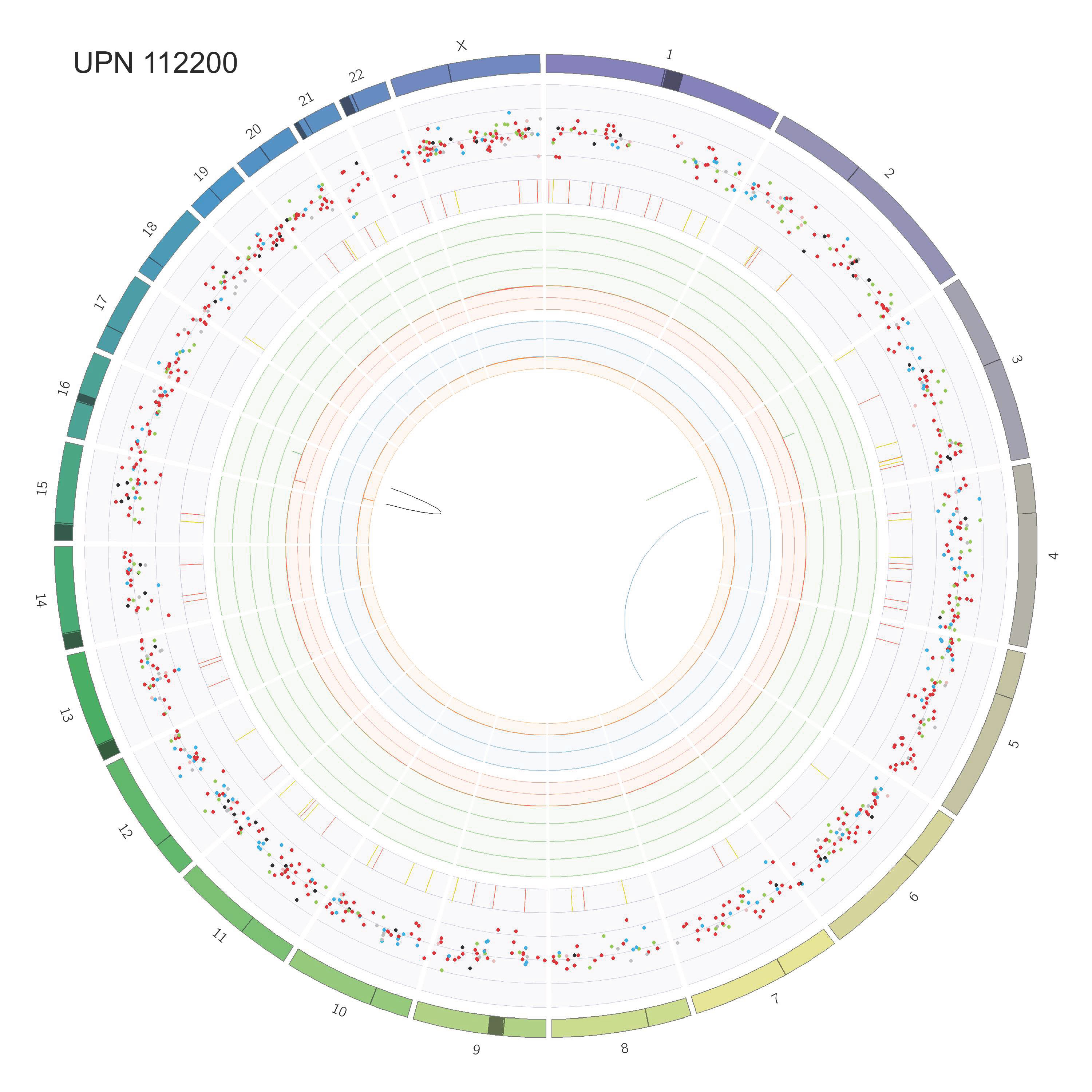

Supplement: Supplement 3 — Supplementary Figure 2. Circos plots [file media-3.zip › Supp_Fig_2_circos_Page_02.jpg]

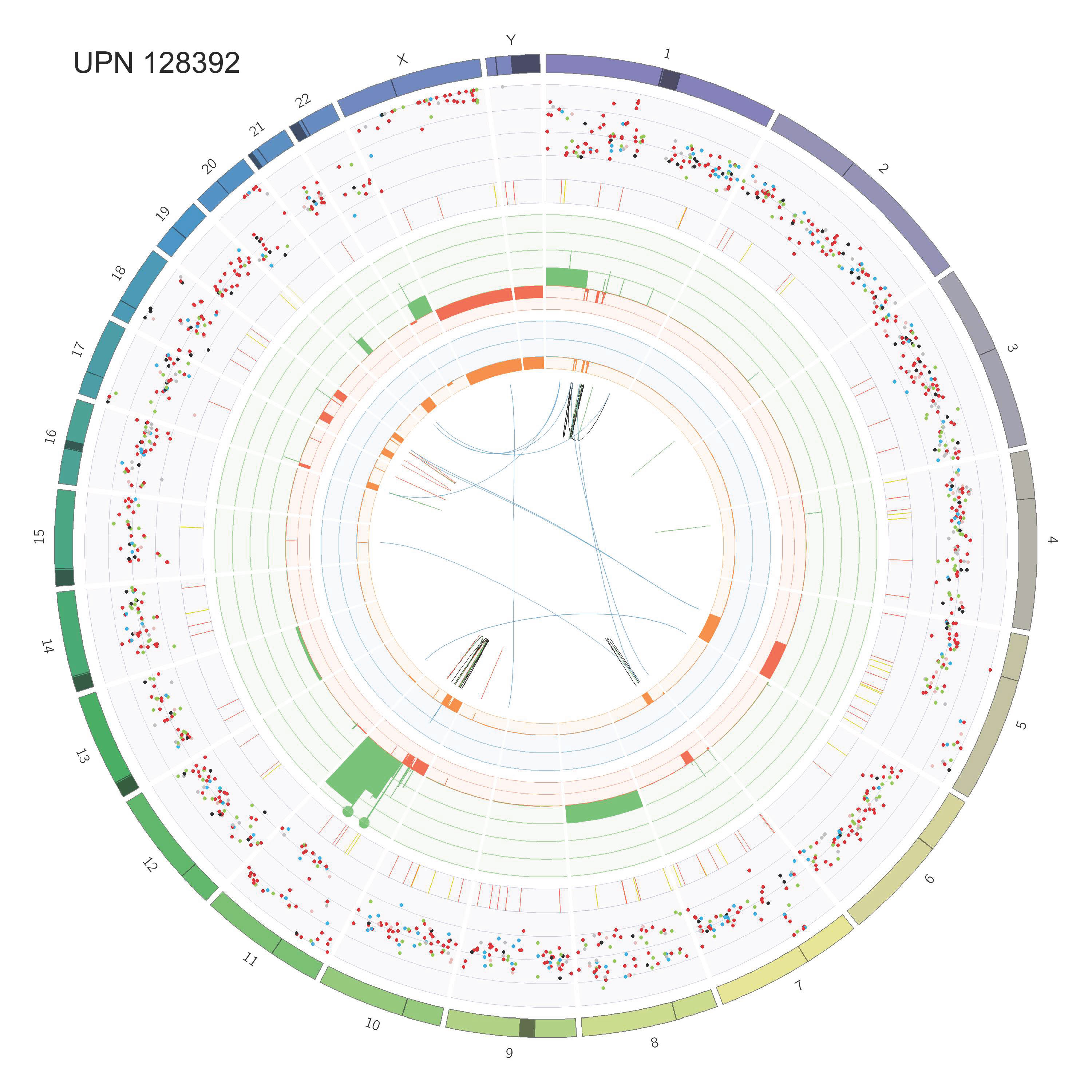

Supplement: Supplement 3 — Supplementary Figure 2. Circos plots [file media-3.zip › Supp_Fig_2_circos_Page_03.jpg]

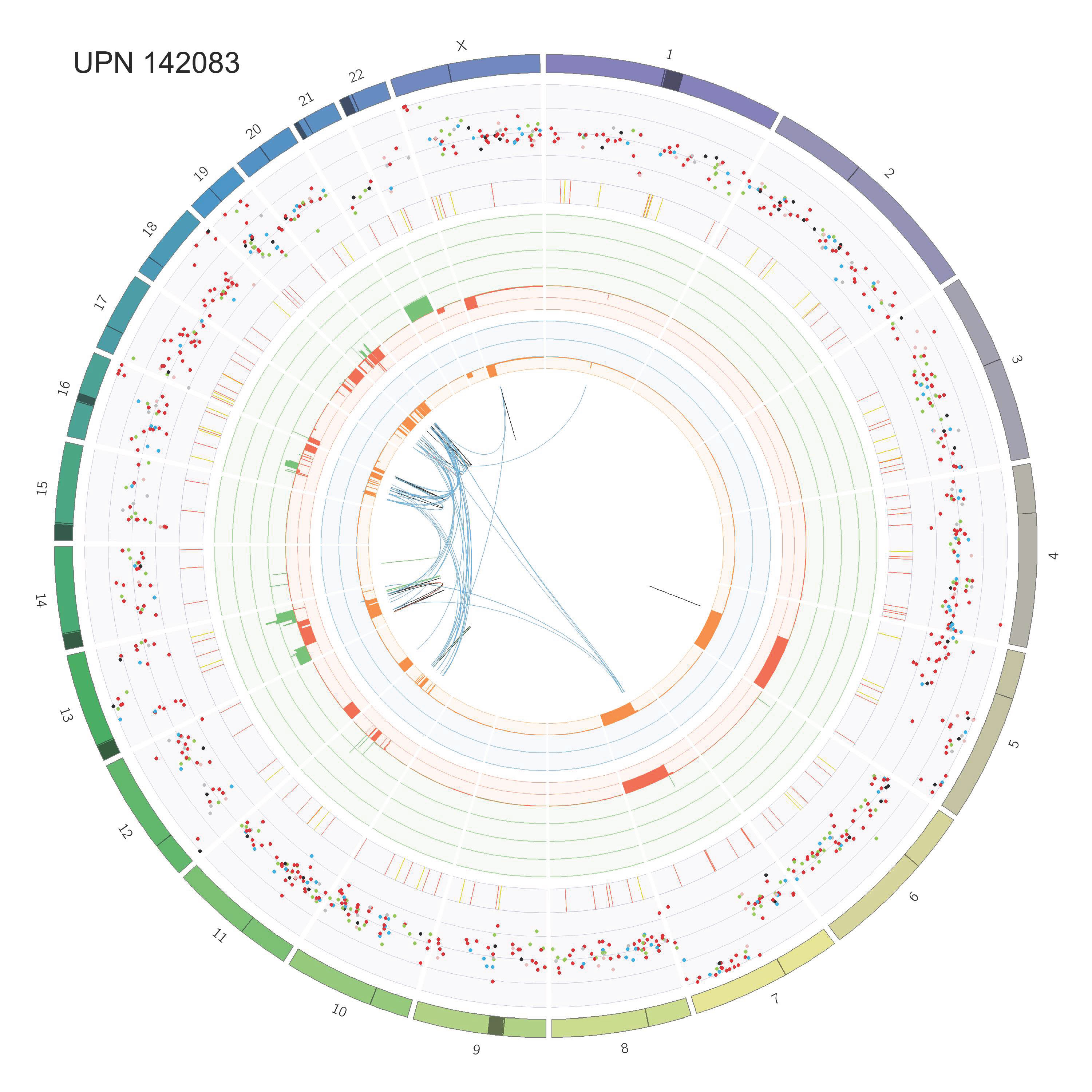

Supplement: Supplement 3 — Supplementary Figure 2. Circos plots [file media-3.zip › Supp_Fig_2_circos_Page_04.jpg]

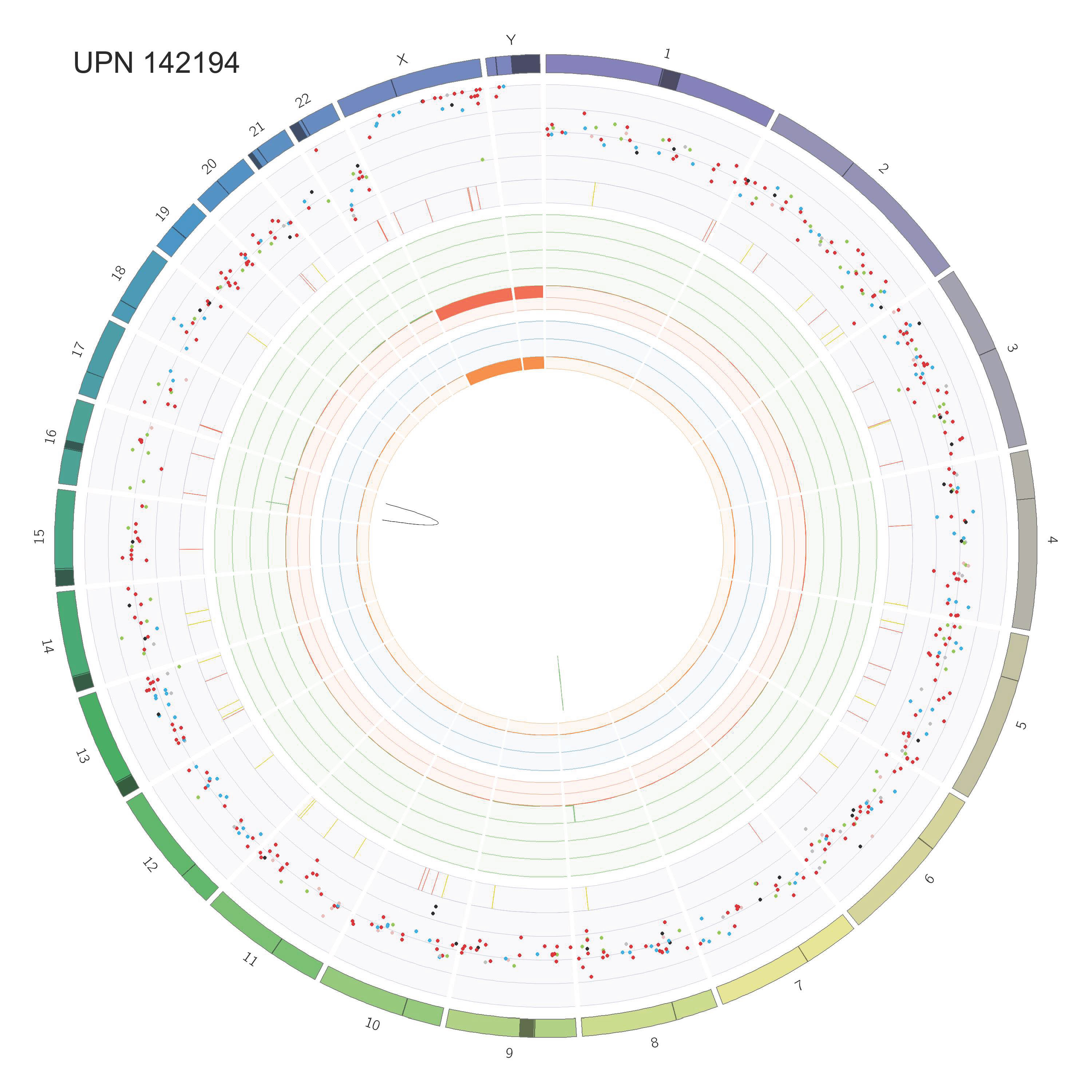

Supplement: Supplement 3 — Supplementary Figure 2. Circos plots [file media-3.zip › Supp_Fig_2_circos_Page_05.jpg]

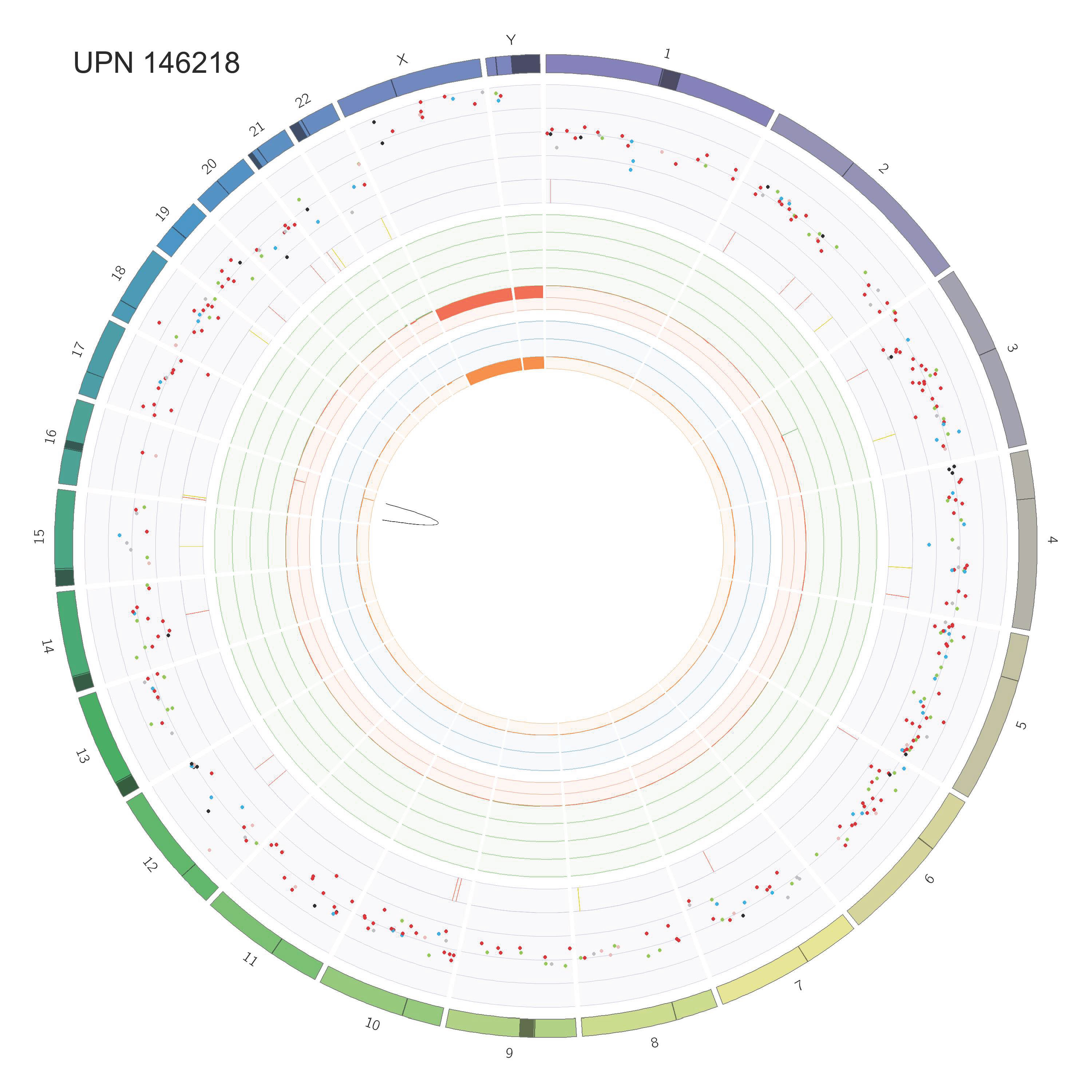

Supplement: Supplement 3 — Supplementary Figure 2. Circos plots [file media-3.zip › Supp_Fig_2_circos_Page_06.jpg]

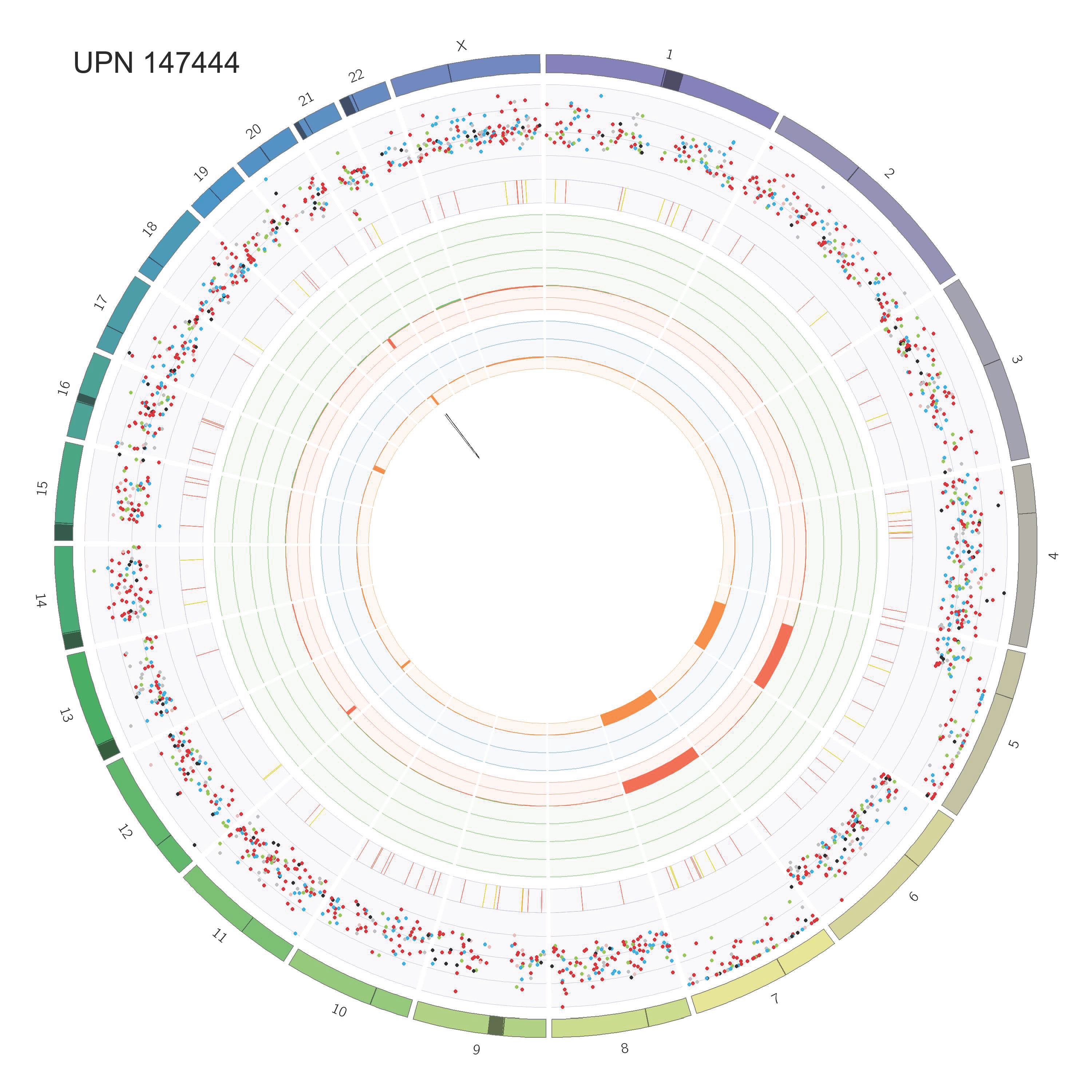

Supplement: Supplement 3 — Supplementary Figure 2. Circos plots [file media-3.zip › Supp_Fig_2_circos_Page_07.jpg]

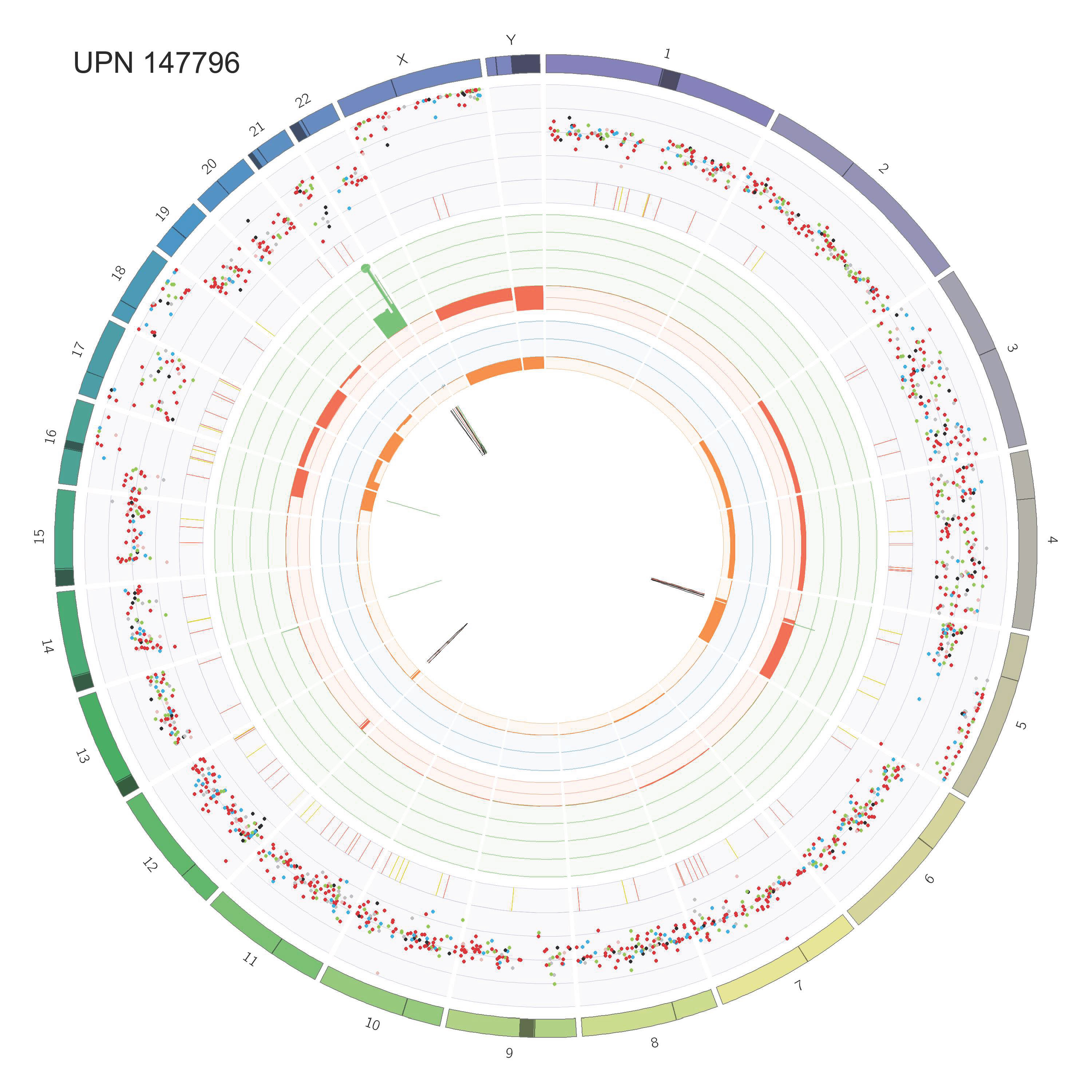

Supplement: Supplement 3 — Supplementary Figure 2. Circos plots [file media-3.zip › Supp_Fig_2_circos_Page_08.jpg]

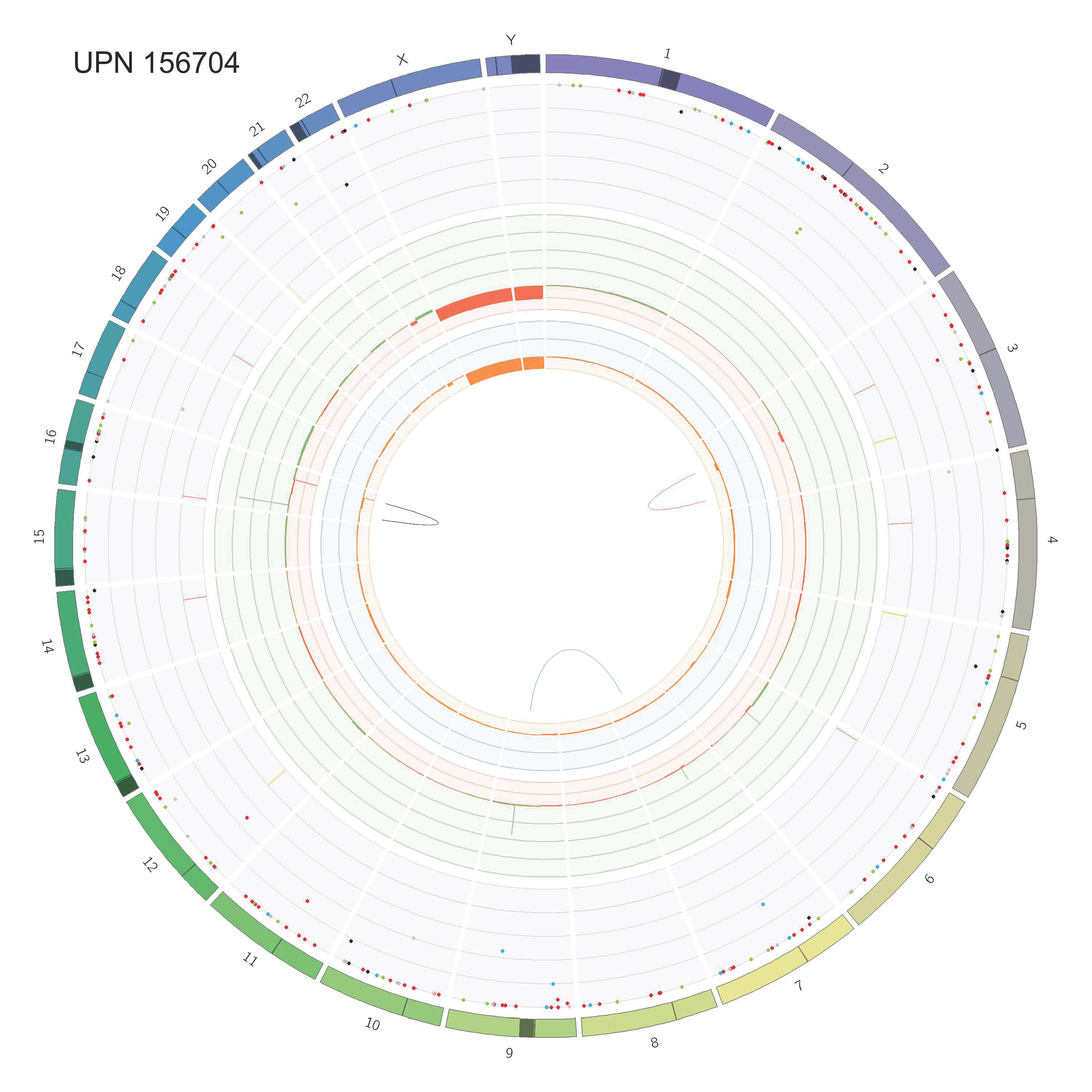

Supplement: Supplement 3 — Supplementary Figure 2. Circos plots [file media-3.zip › Supp_Fig_2_circos_Page_09.jpg]

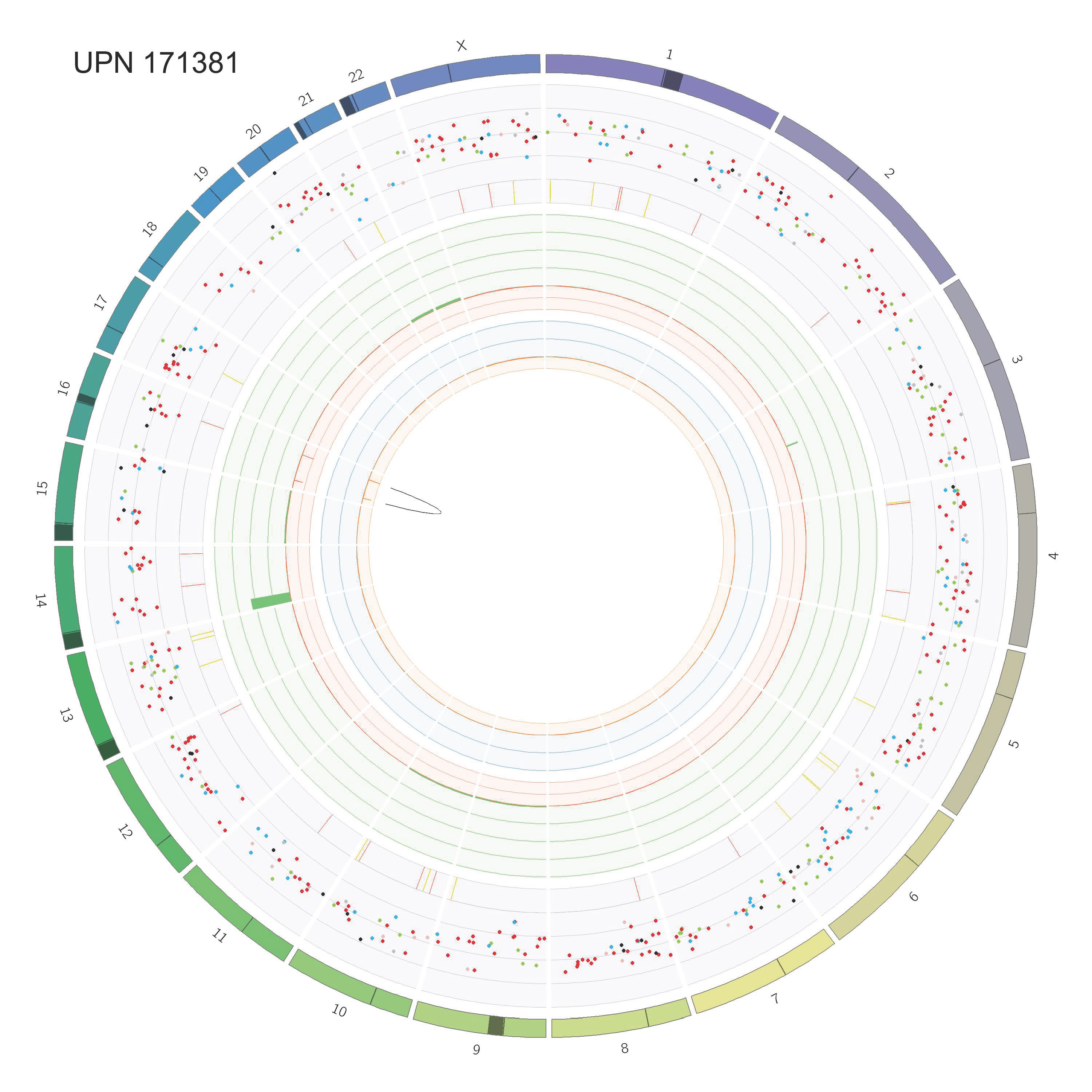

Supplement: Supplement 3 — Supplementary Figure 2. Circos plots [file media-3.zip › Supp_Fig_2_circos_Page_10.jpg]

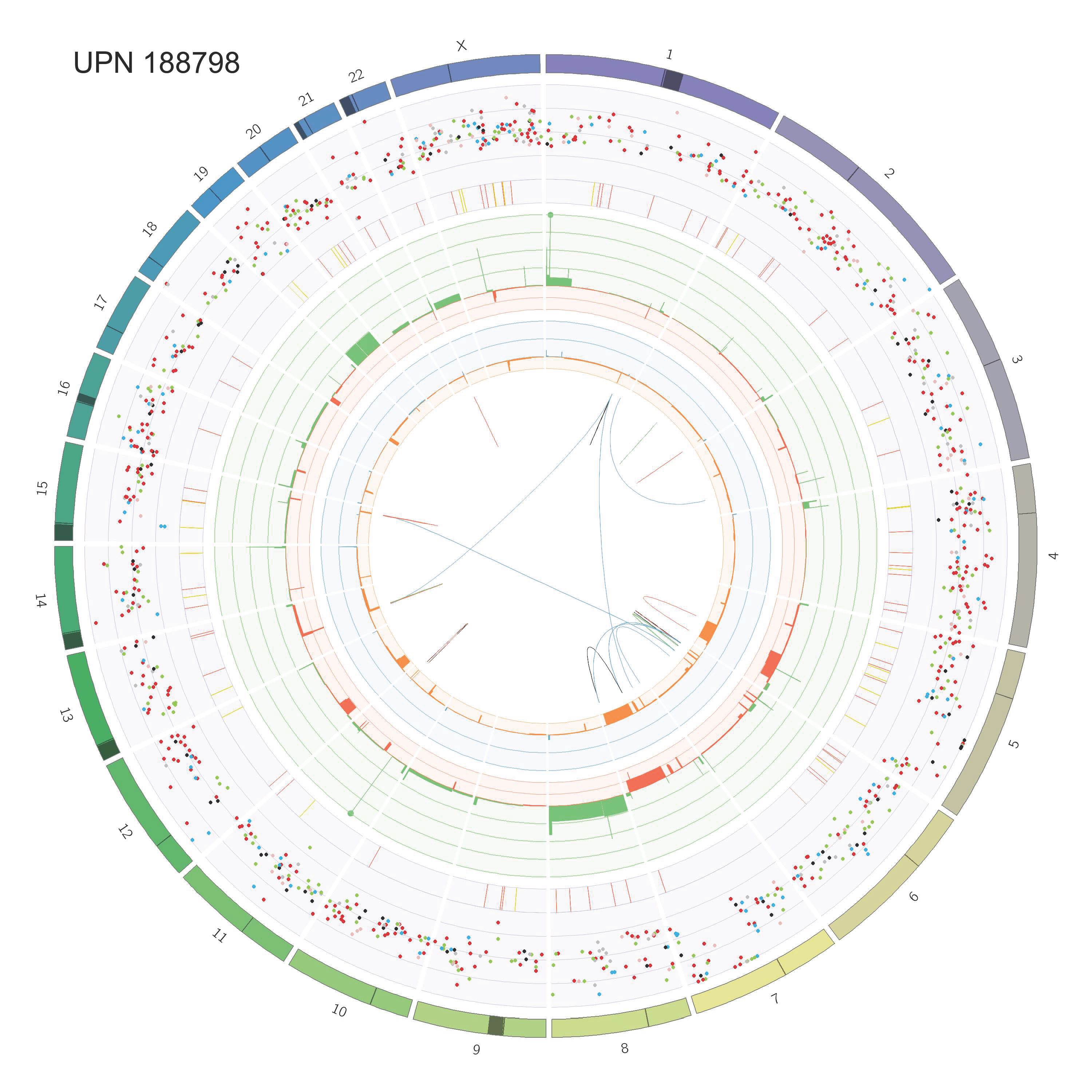

Supplement: Supplement 3 — Supplementary Figure 2. Circos plots [file media-3.zip › Supp_Fig_2_circos_Page_11.jpg]

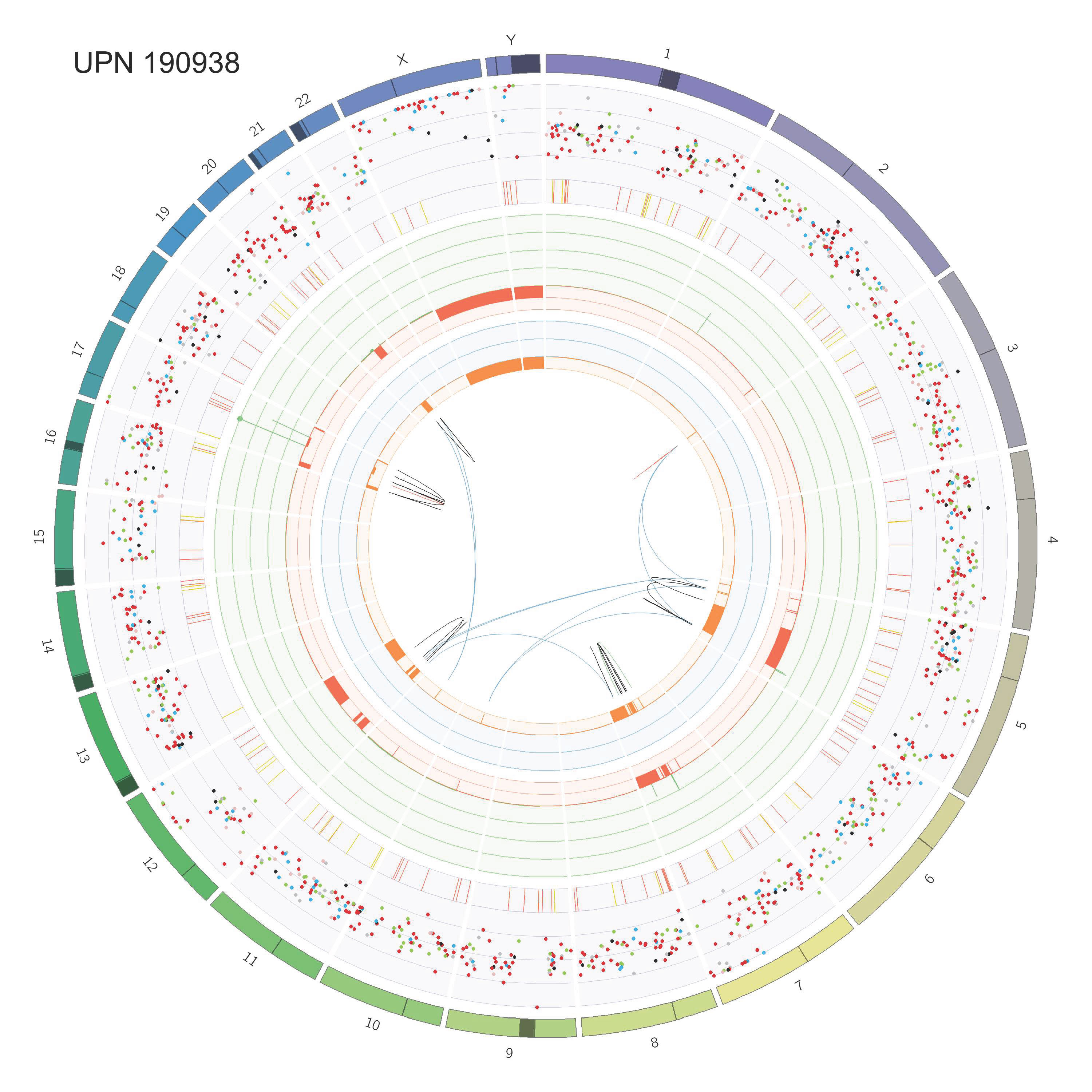

Supplement: Supplement 3 — Supplementary Figure 2. Circos plots [file media-3.zip › Supp_Fig_2_circos_Page_12.jpg]

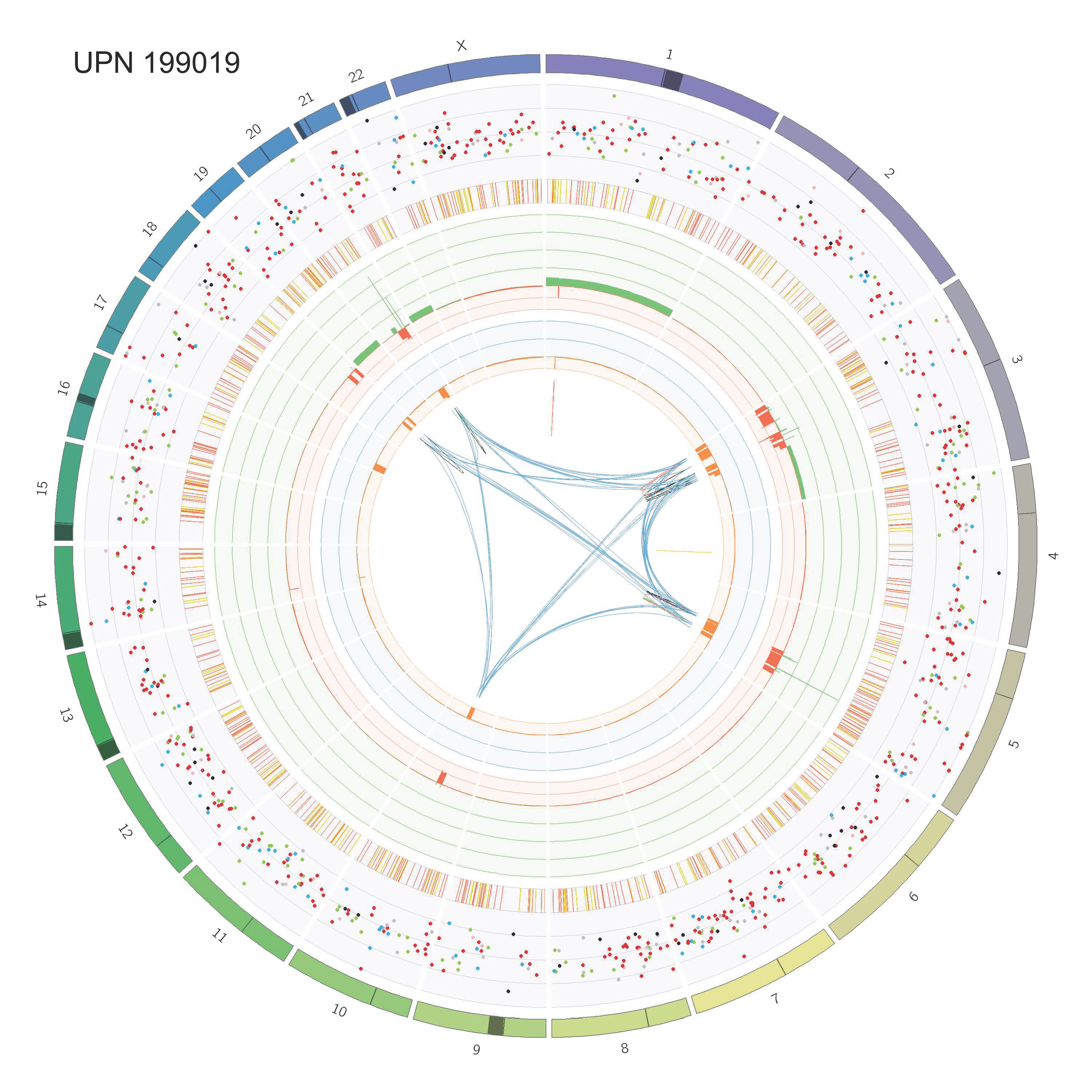

Supplement: Supplement 3 — Supplementary Figure 2. Circos plots [file media-3.zip › Supp_Fig_2_circos_Page_13.jpg]

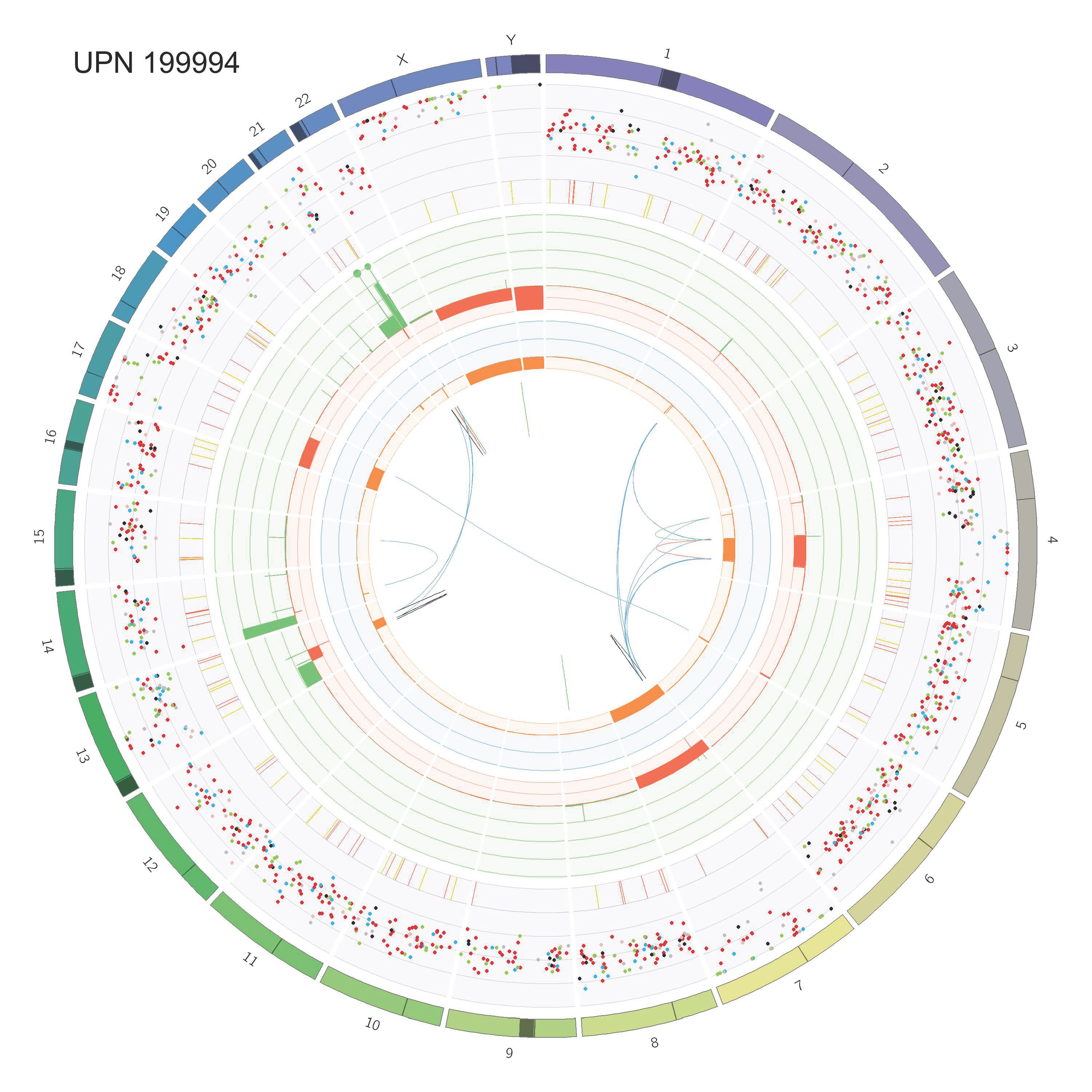

Supplement: Supplement 3 — Supplementary Figure 2. Circos plots [file media-3.zip › Supp_Fig_2_circos_Page_14.jpg]

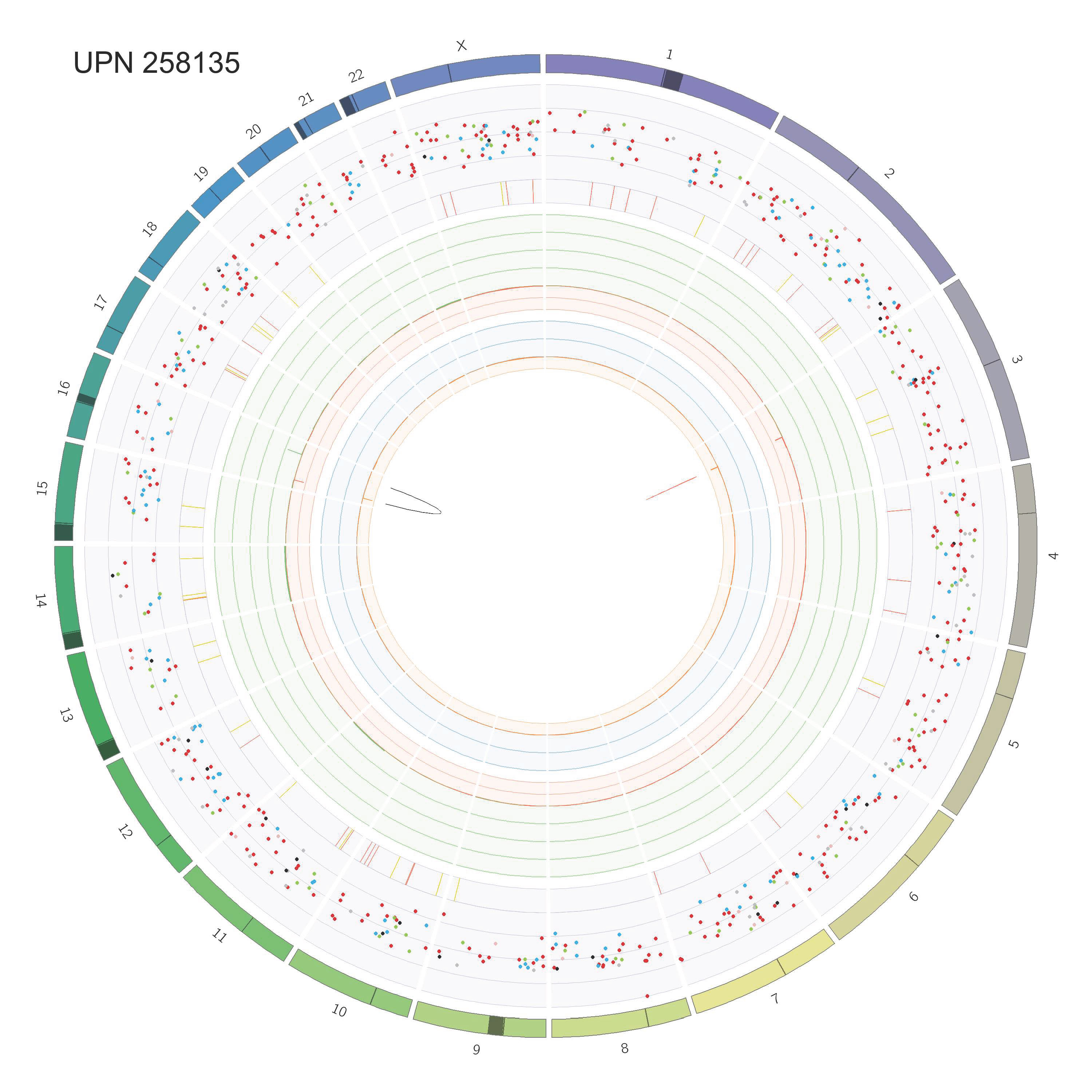

Supplement: Supplement 3 — Supplementary Figure 2. Circos plots [file media-3.zip › Supp_Fig_2_circos_Page_15.jpg]

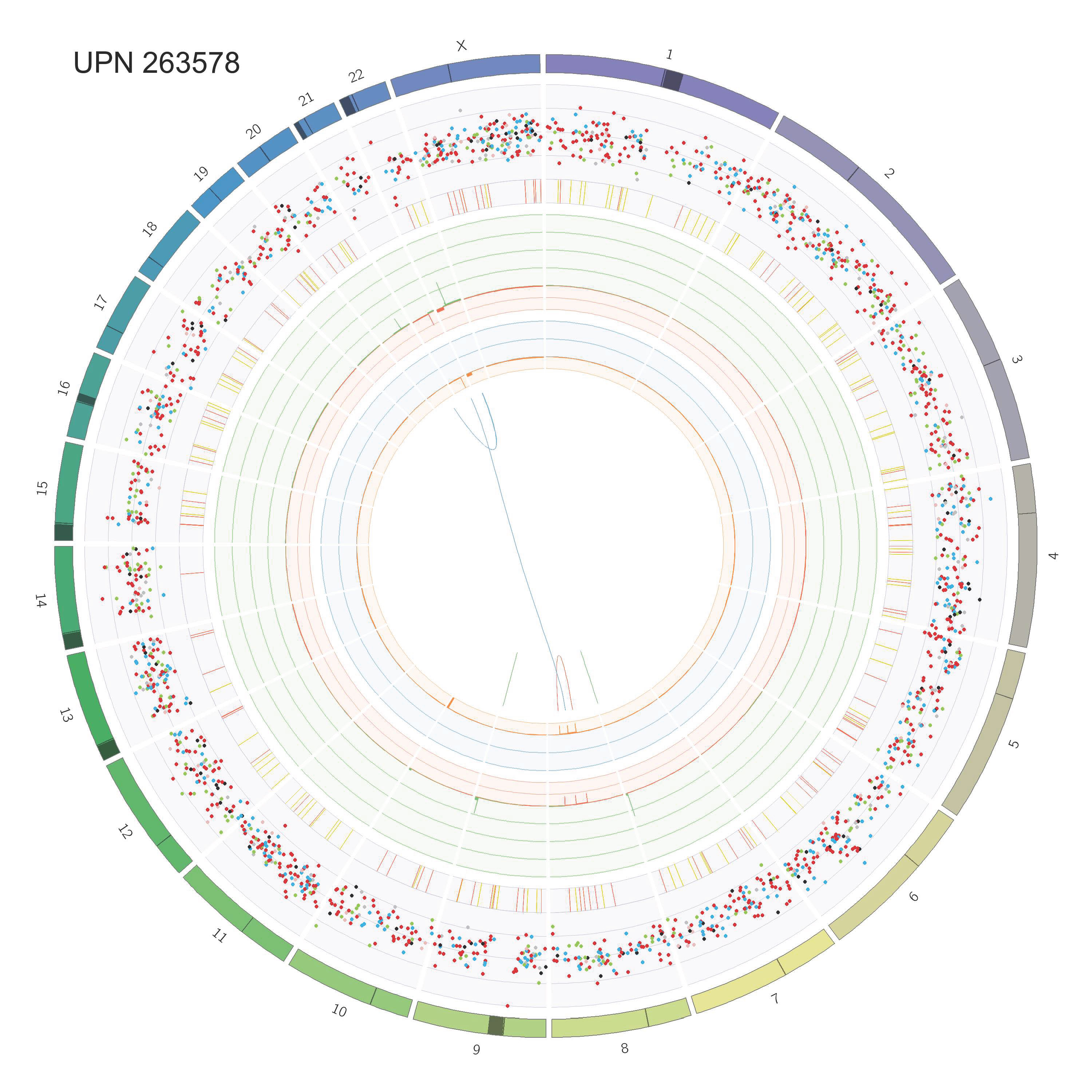

Supplement: Supplement 3 — Supplementary Figure 2. Circos plots [file media-3.zip › Supp_Fig_2_circos_Page_16.jpg]

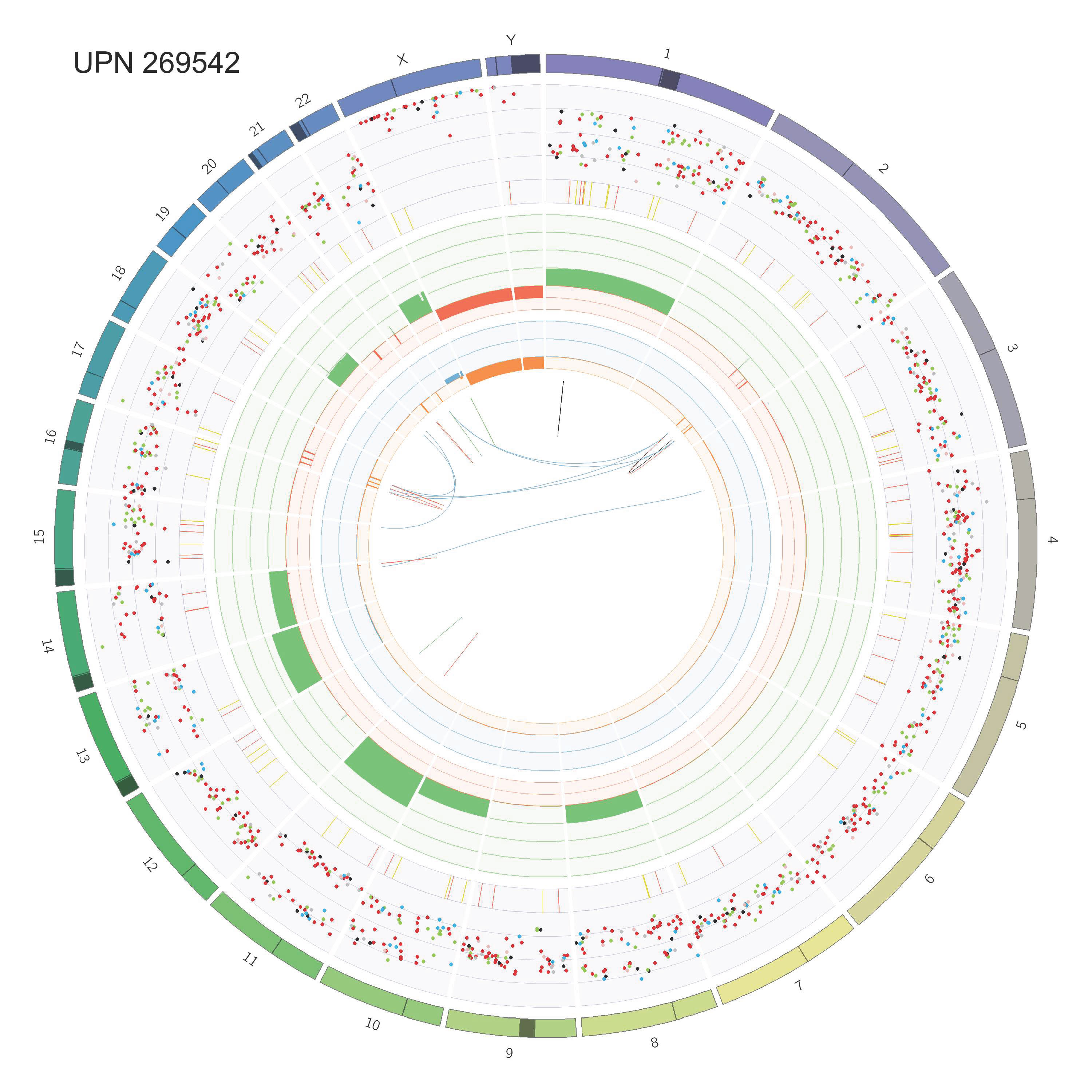

Supplement: Supplement 3 — Supplementary Figure 2. Circos plots [file media-3.zip › Supp_Fig_2_circos_Page_17.jpg]

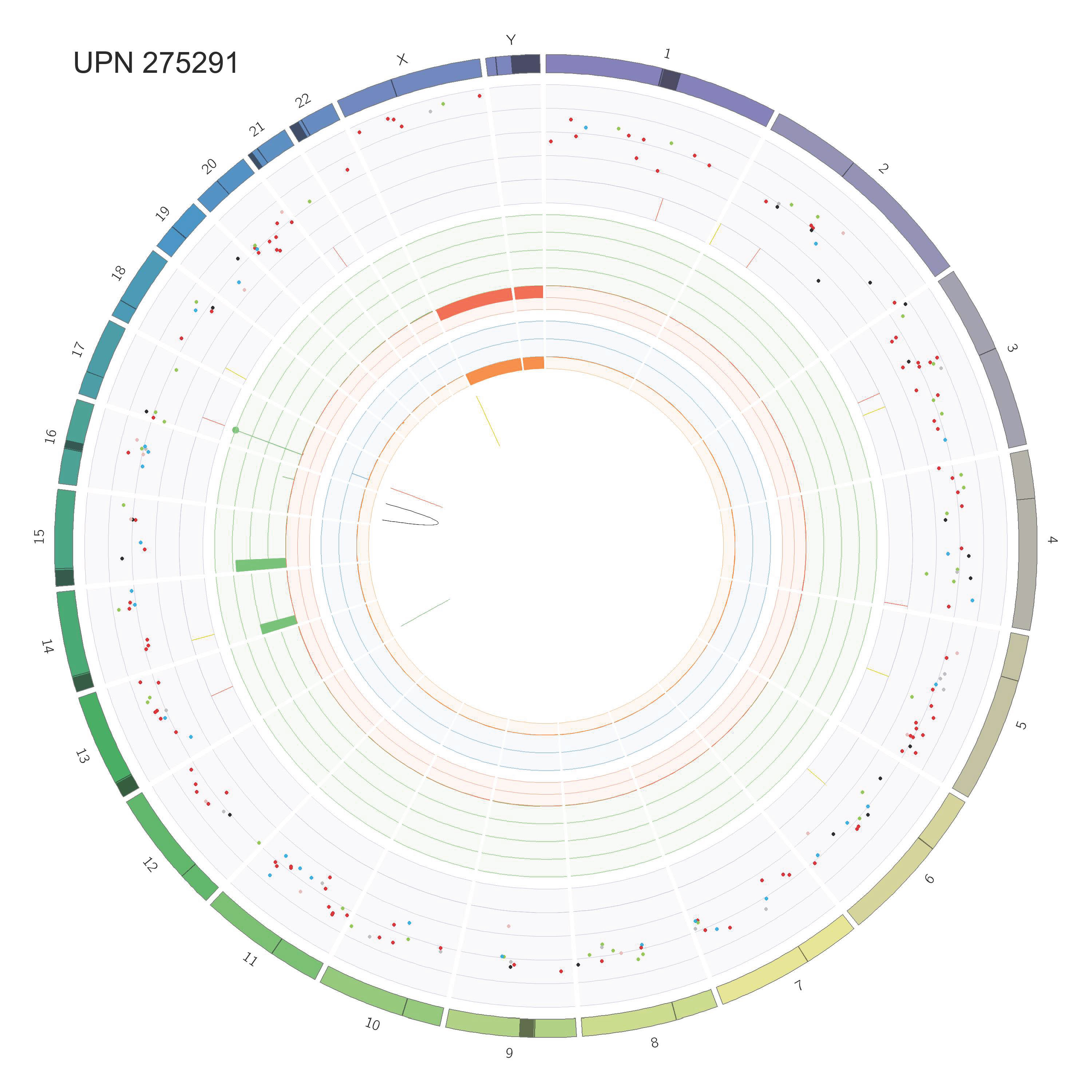

Supplement: Supplement 3 — Supplementary Figure 2. Circos plots [file media-3.zip › Supp_Fig_2_circos_Page_18.jpg]

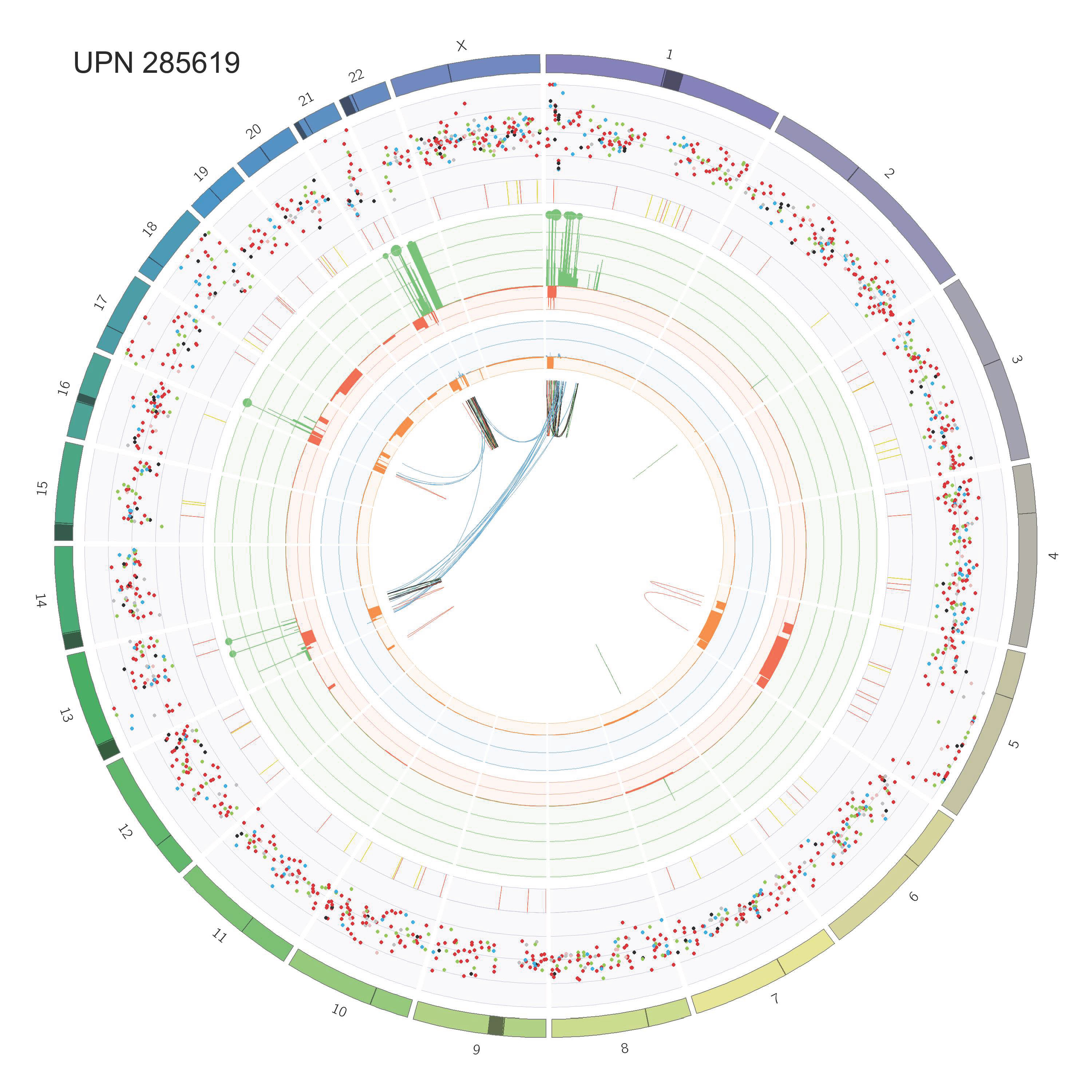

Supplement: Supplement 3 — Supplementary Figure 2. Circos plots [file media-3.zip › Supp_Fig_2_circos_Page_19.jpg]

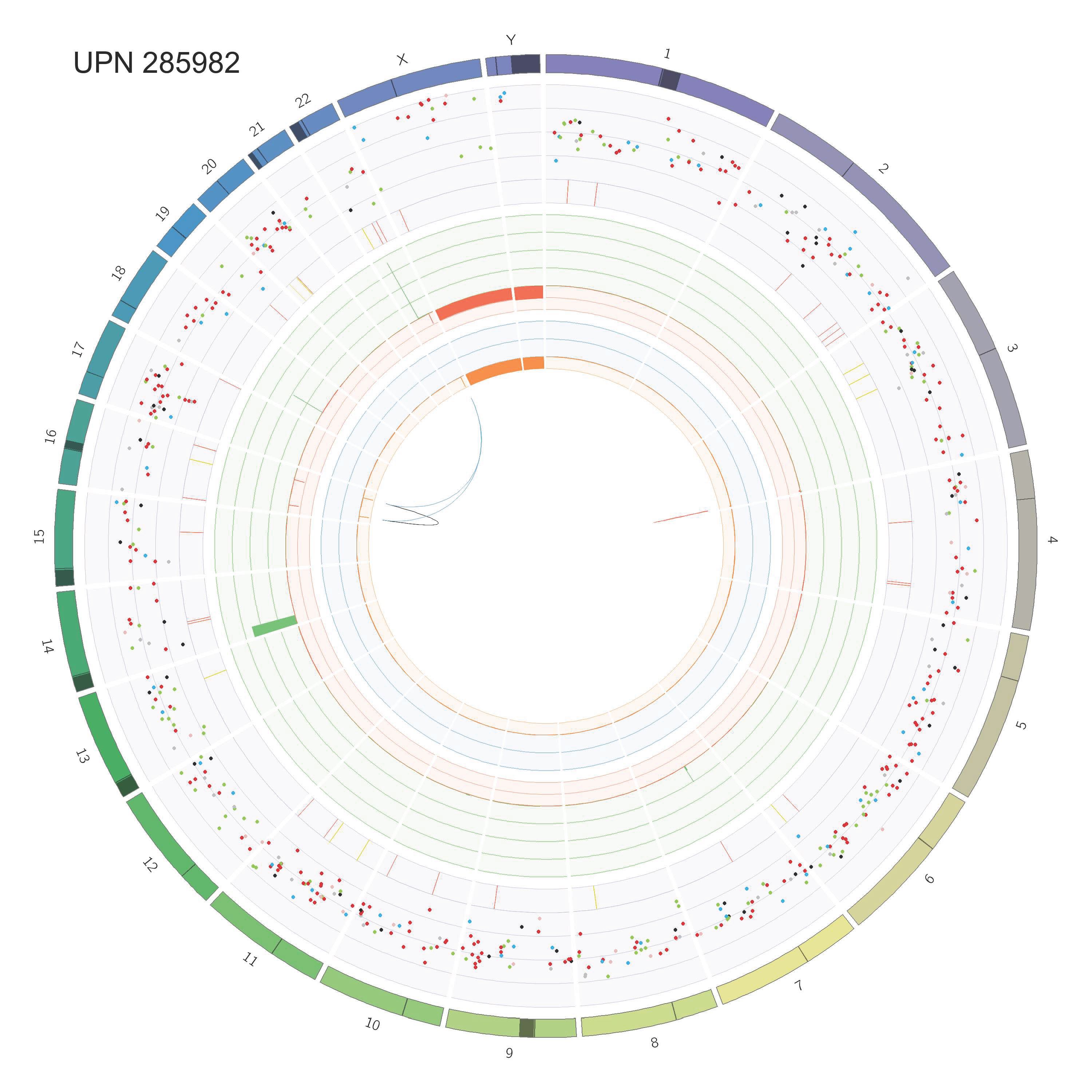

Supplement: Supplement 3 — Supplementary Figure 2. Circos plots [file media-3.zip › Supp_Fig_2_circos_Page_20.jpg]

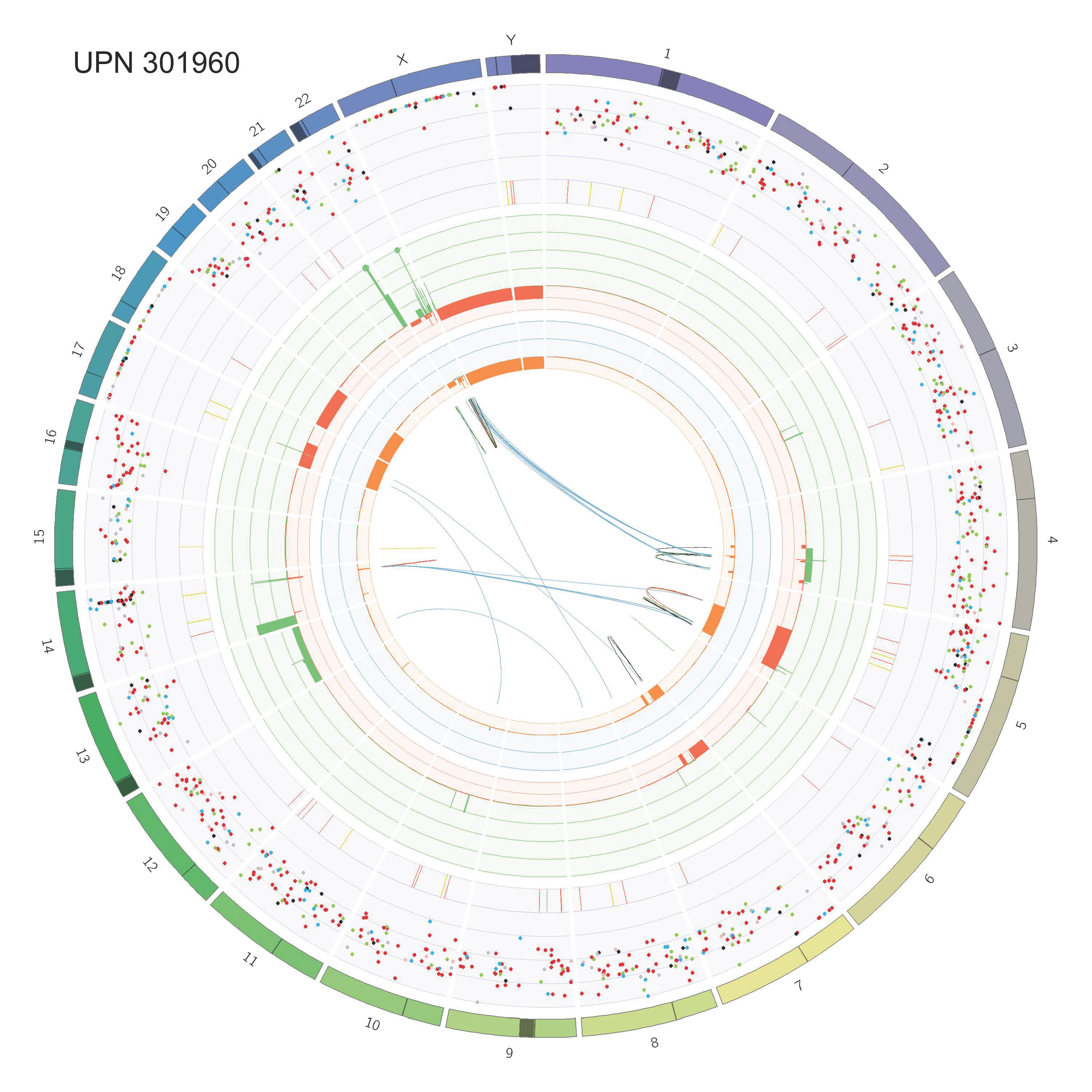

Supplement: Supplement 3 — Supplementary Figure 2. Circos plots [file media-3.zip › Supp_Fig_2_circos_Page_21.jpg]

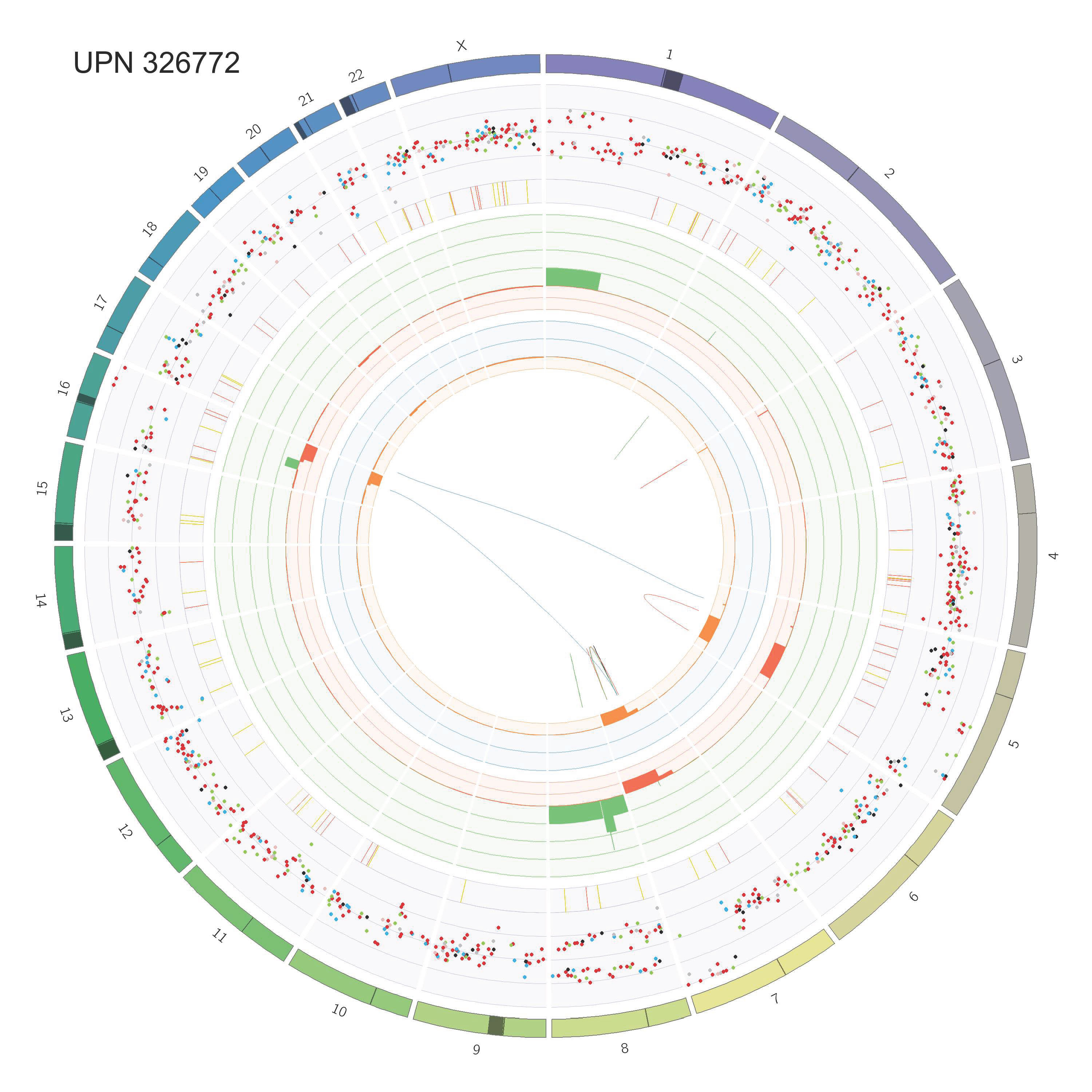

Supplement: Supplement 3 — Supplementary Figure 2. Circos plots [file media-3.zip › Supp_Fig_2_circos_Page_22.jpg]

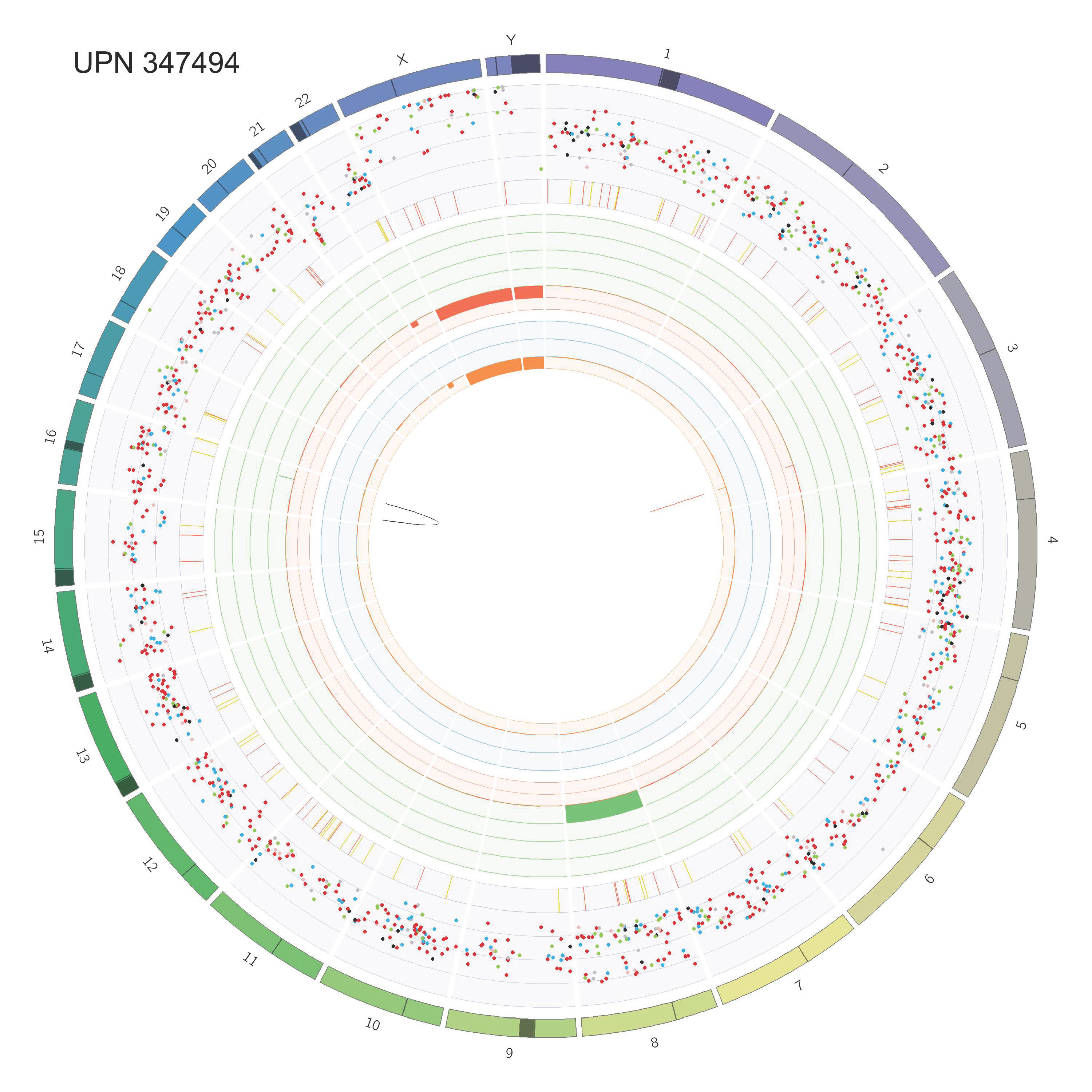

Supplement: Supplement 3 — Supplementary Figure 2. Circos plots [file media-3.zip › Supp_Fig_2_circos_Page_23.jpg]

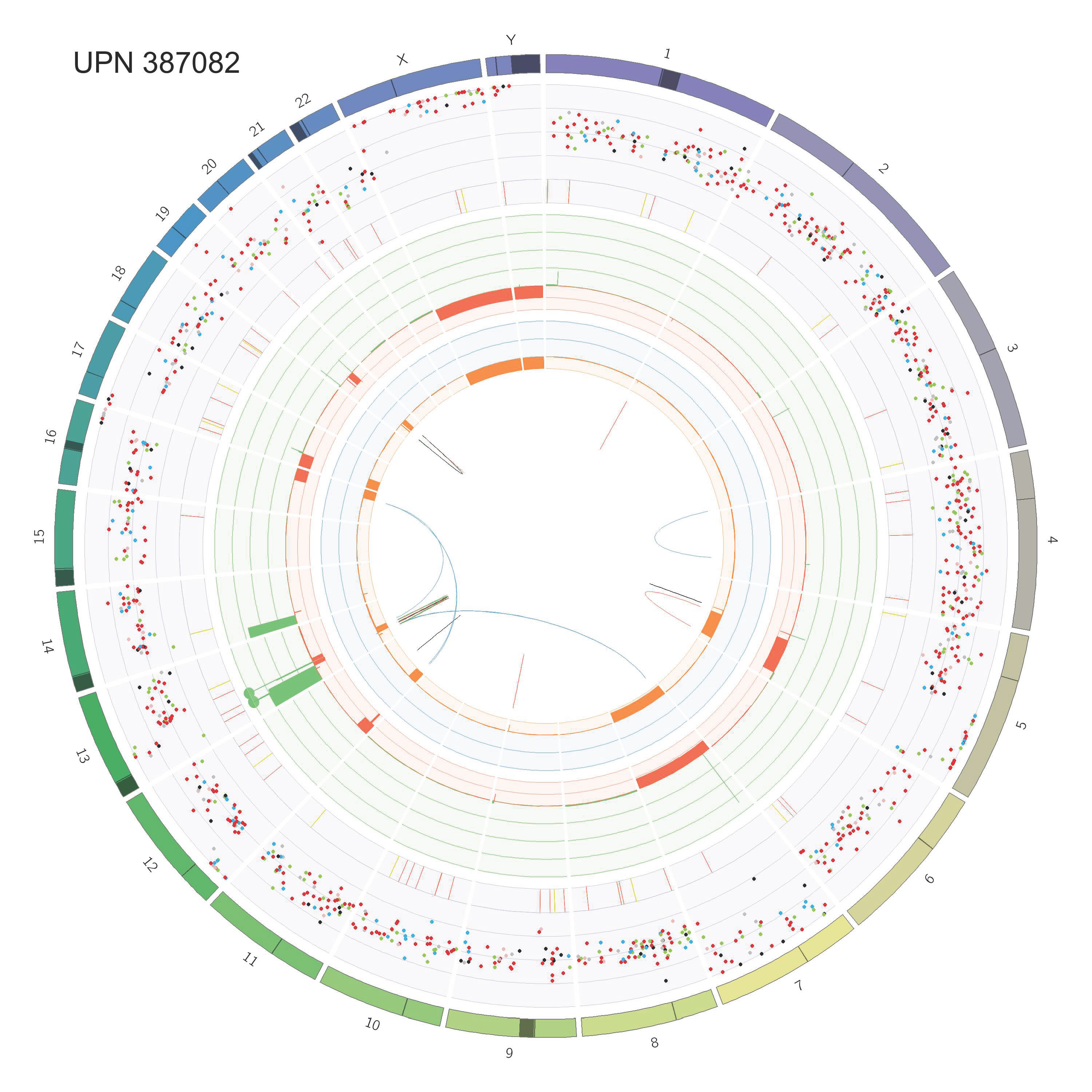

Supplement: Supplement 3 — Supplementary Figure 2. Circos plots [file media-3.zip › Supp_Fig_2_circos_Page_24.jpg]

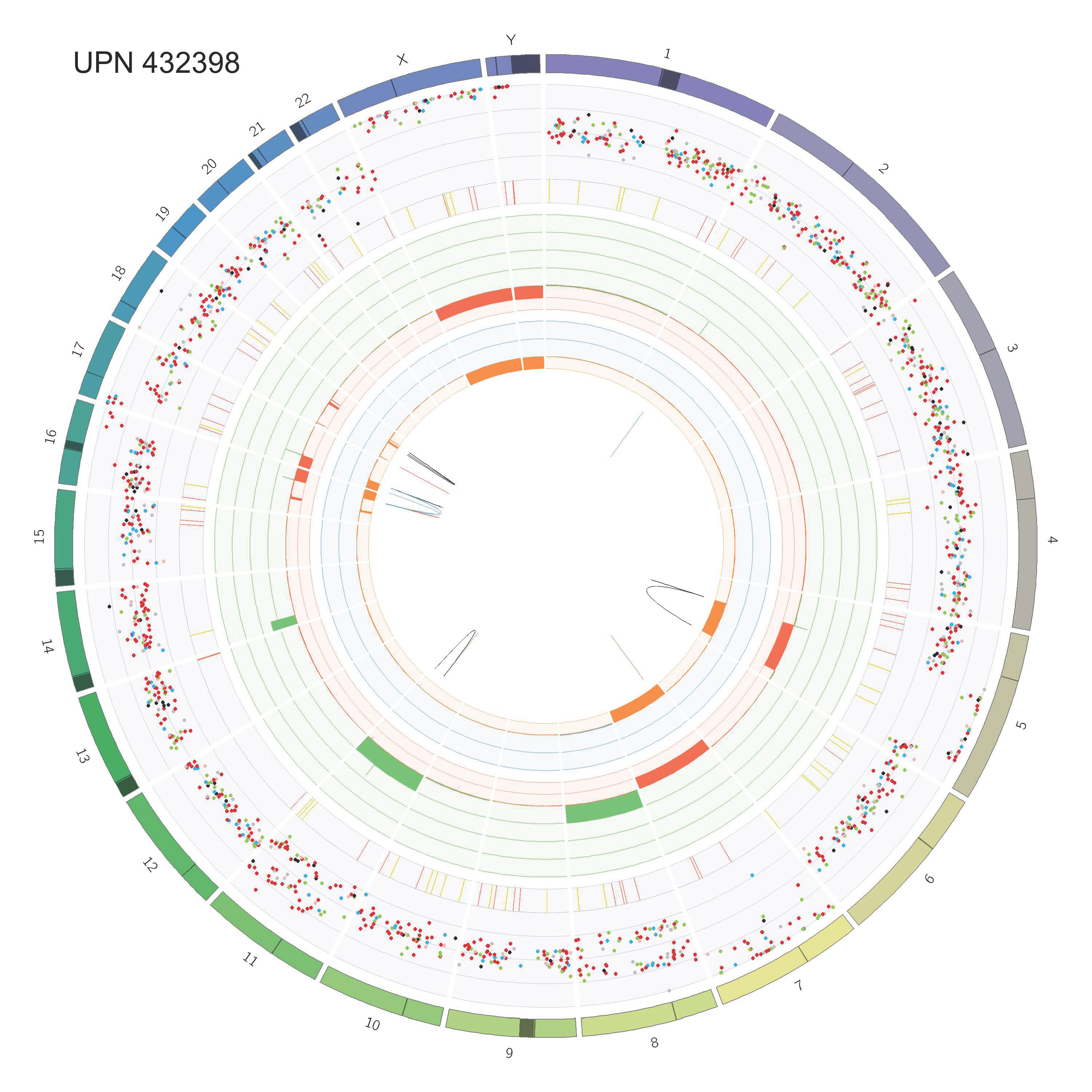

Supplement: Supplement 3 — Supplementary Figure 2. Circos plots [file media-3.zip › Supp_Fig_2_circos_Page_25.jpg]

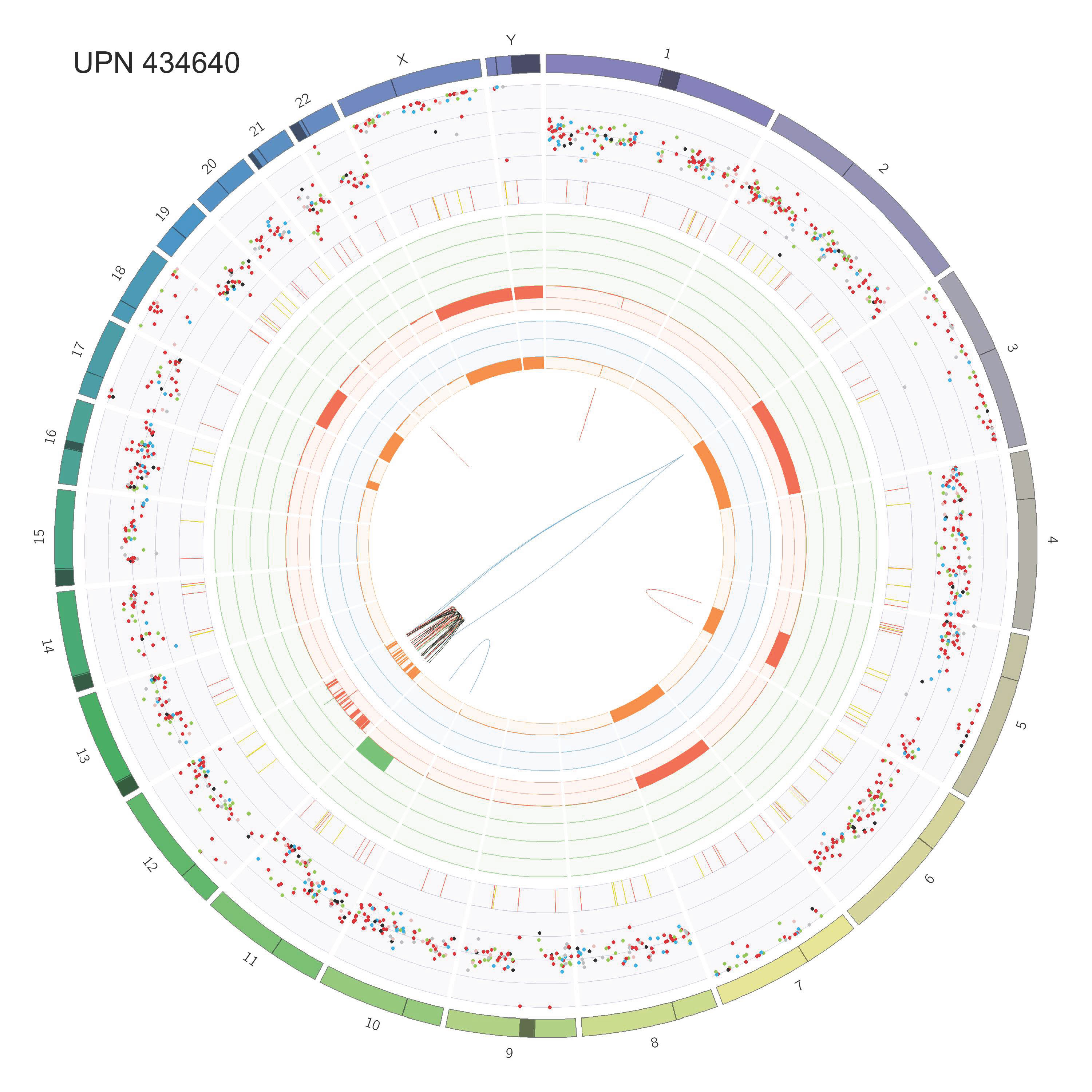

Supplement: Supplement 3 — Supplementary Figure 2. Circos plots [file media-3.zip › Supp_Fig_2_circos_Page_26.jpg]

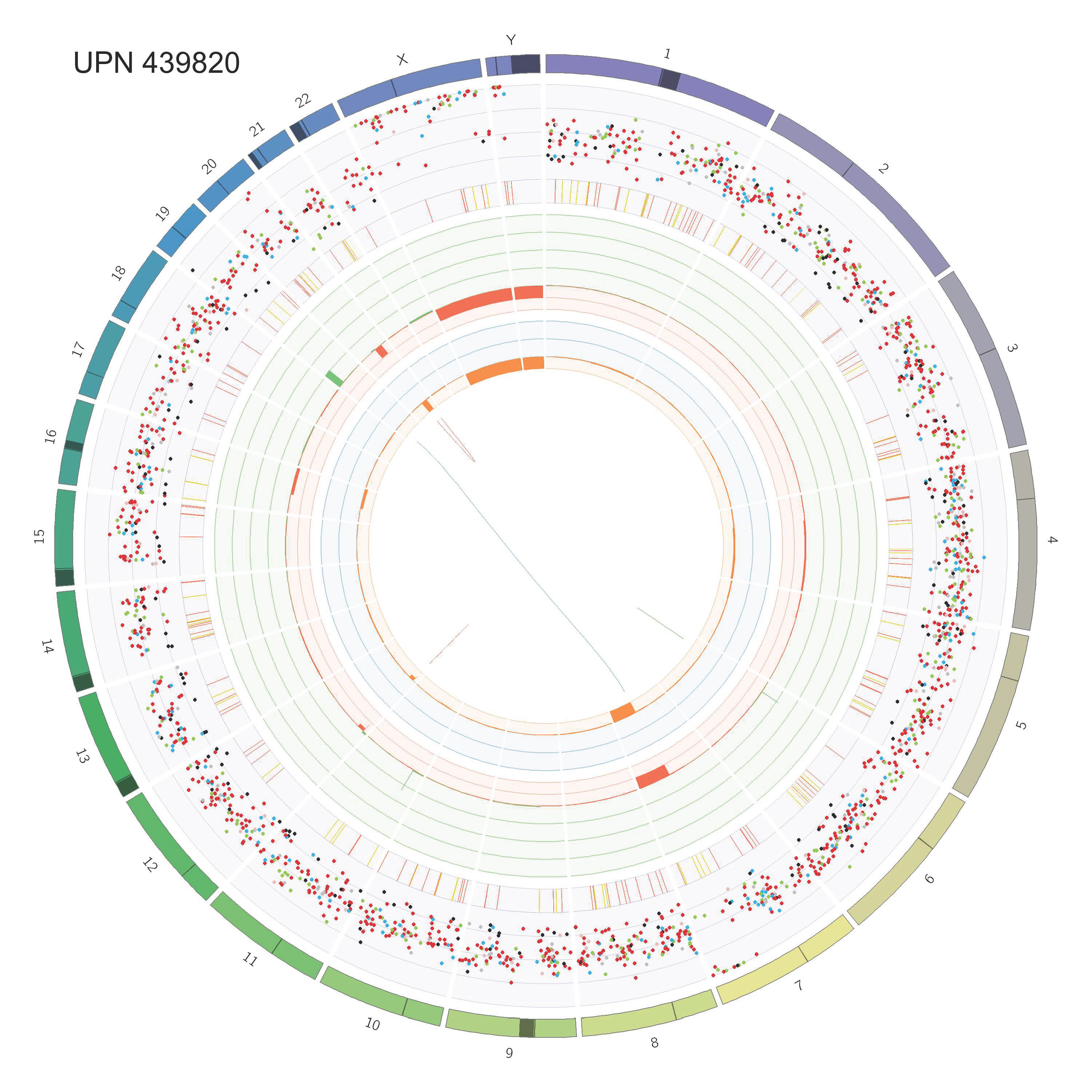

Supplement: Supplement 3 — Supplementary Figure 2. Circos plots [file media-3.zip › Supp_Fig_2_circos_Page_27.jpg]

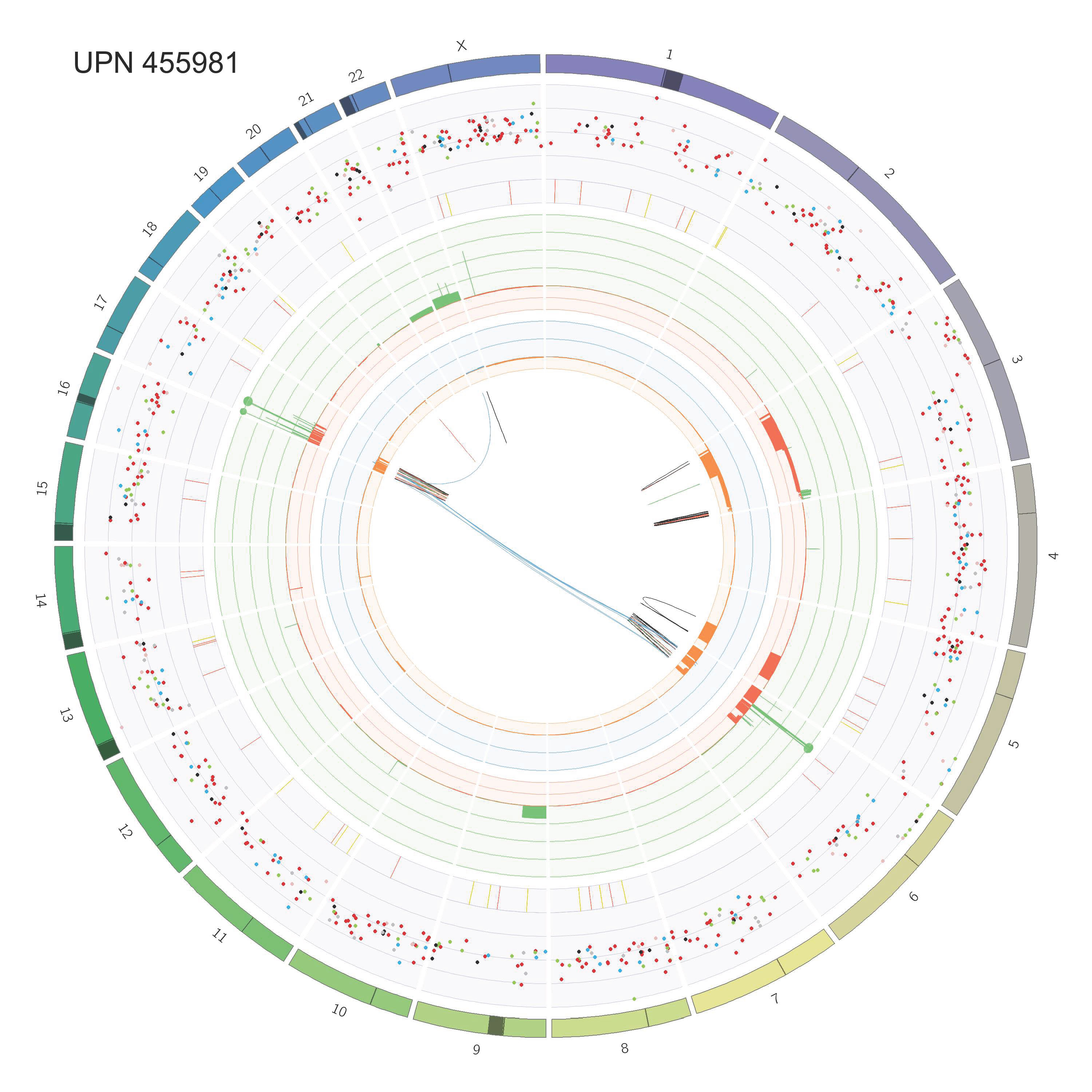

Supplement: Supplement 3 — Supplementary Figure 2. Circos plots [file media-3.zip › Supp_Fig_2_circos_Page_28.jpg]

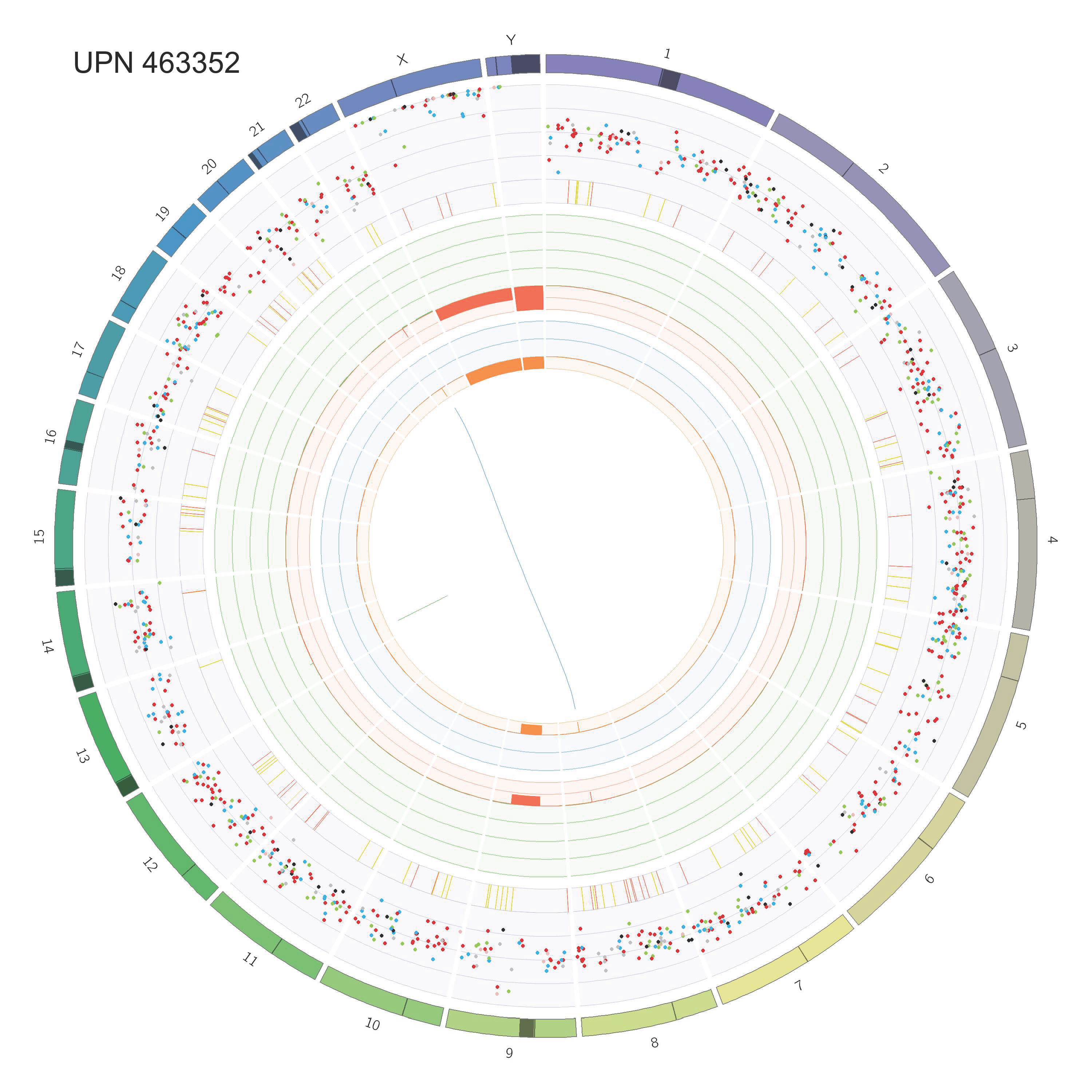

Supplement: Supplement 3 — Supplementary Figure 2. Circos plots [file media-3.zip › Supp_Fig_2_circos_Page_29.jpg]

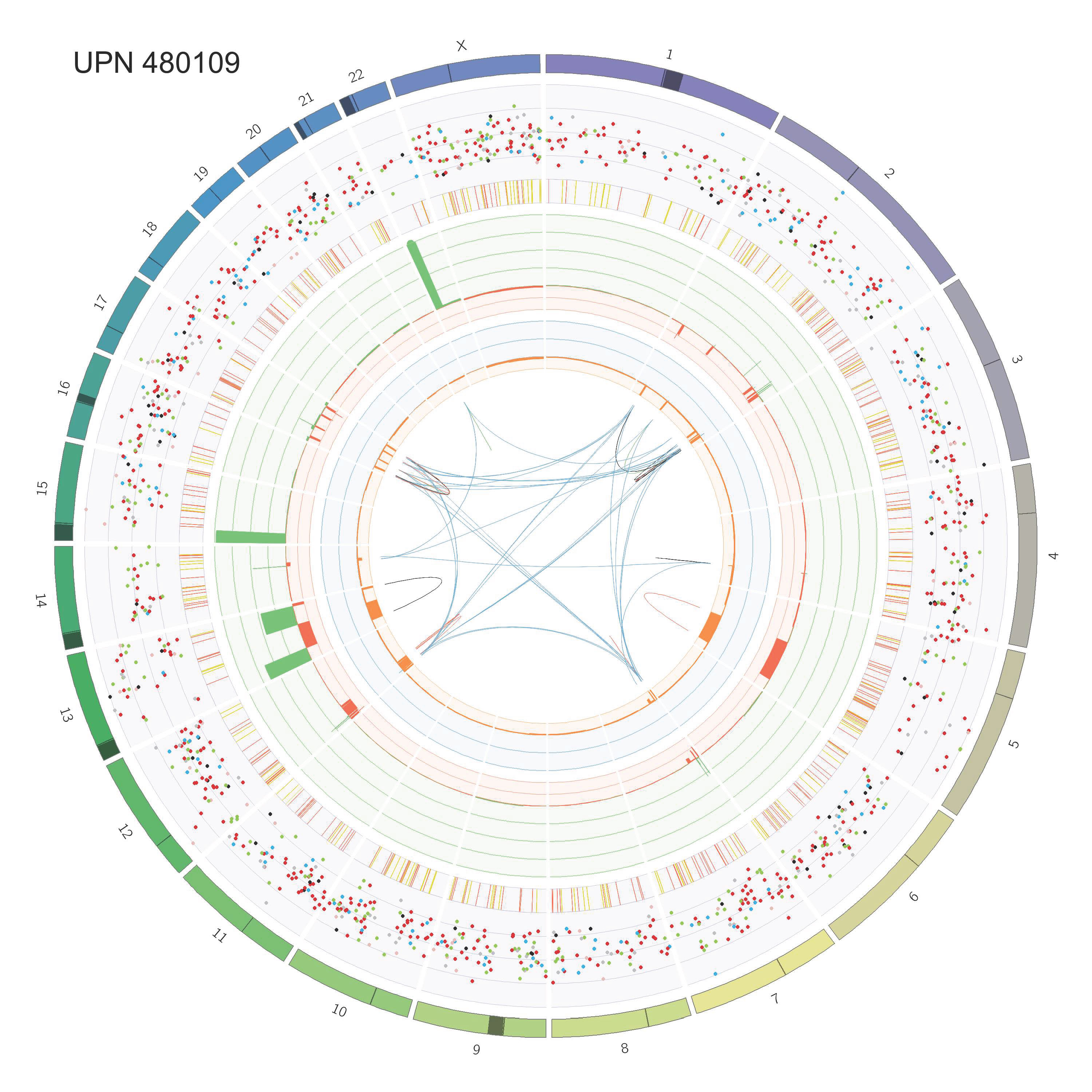

Supplement: Supplement 3 — Supplementary Figure 2. Circos plots [file media-3.zip › Supp_Fig_2_circos_Page_30.jpg]

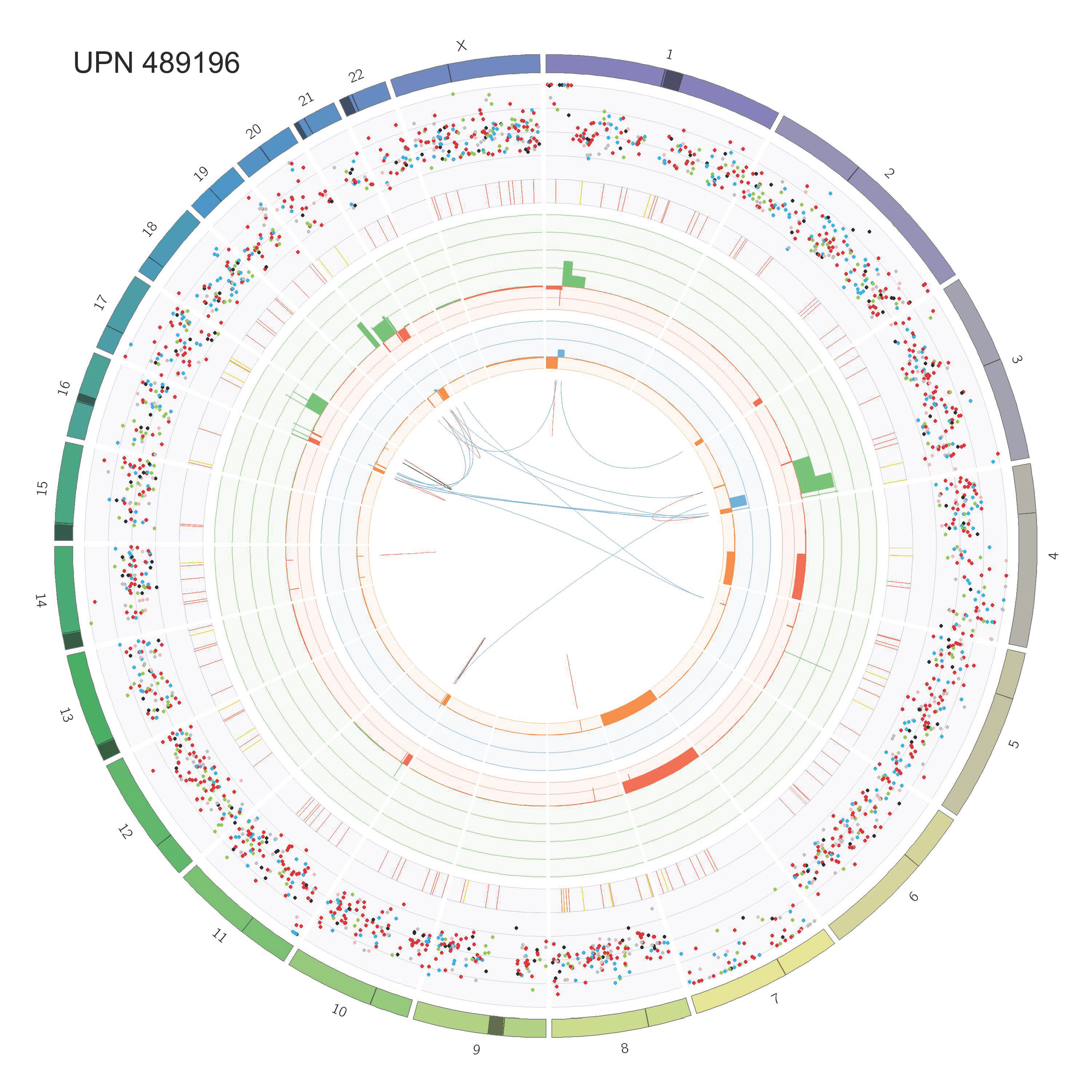

Supplement: Supplement 3 — Supplementary Figure 2. Circos plots [file media-3.zip › Supp_Fig_2_circos_Page_31.jpg]

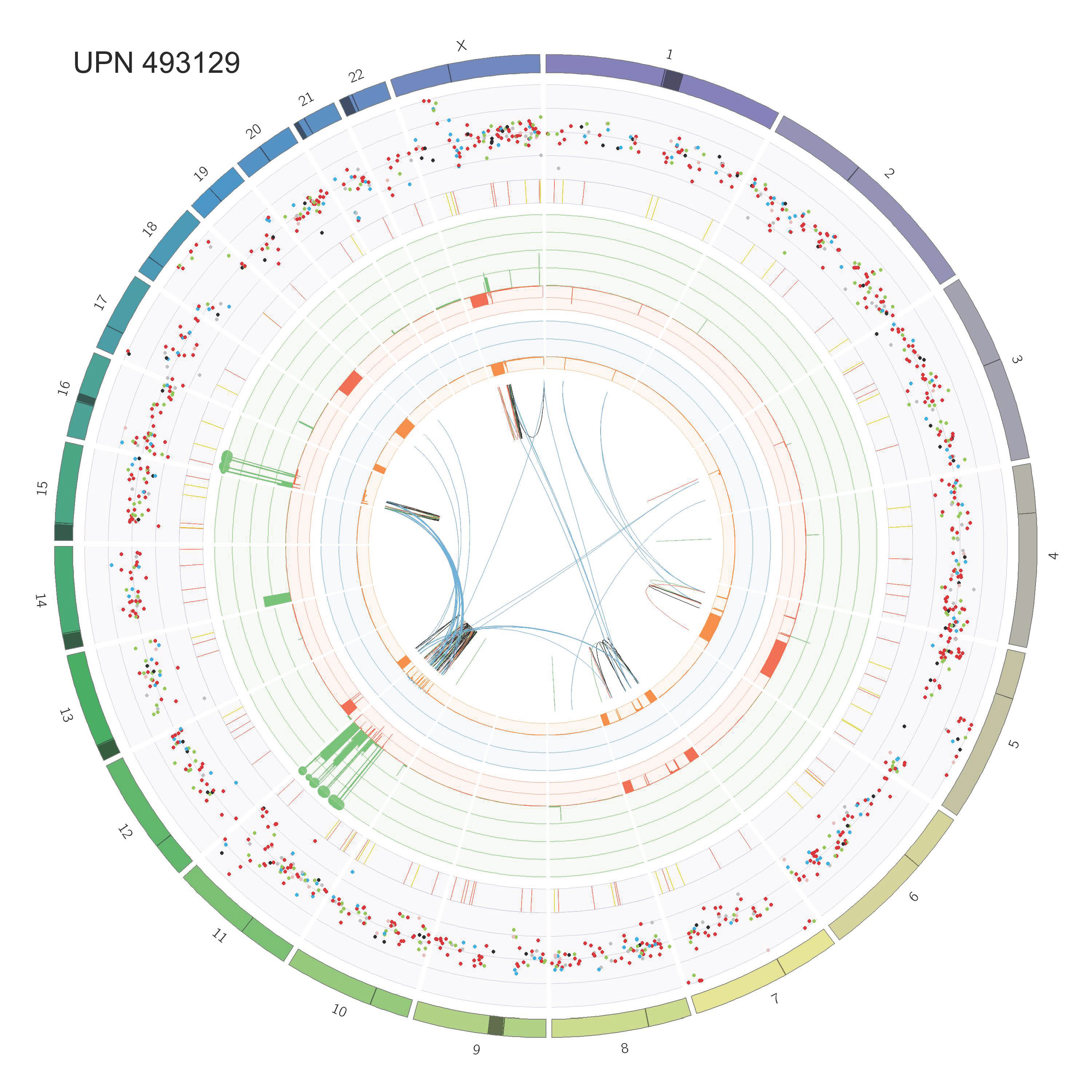

Supplement: Supplement 3 — Supplementary Figure 2. Circos plots [file media-3.zip › Supp_Fig_2_circos_Page_32.jpg]

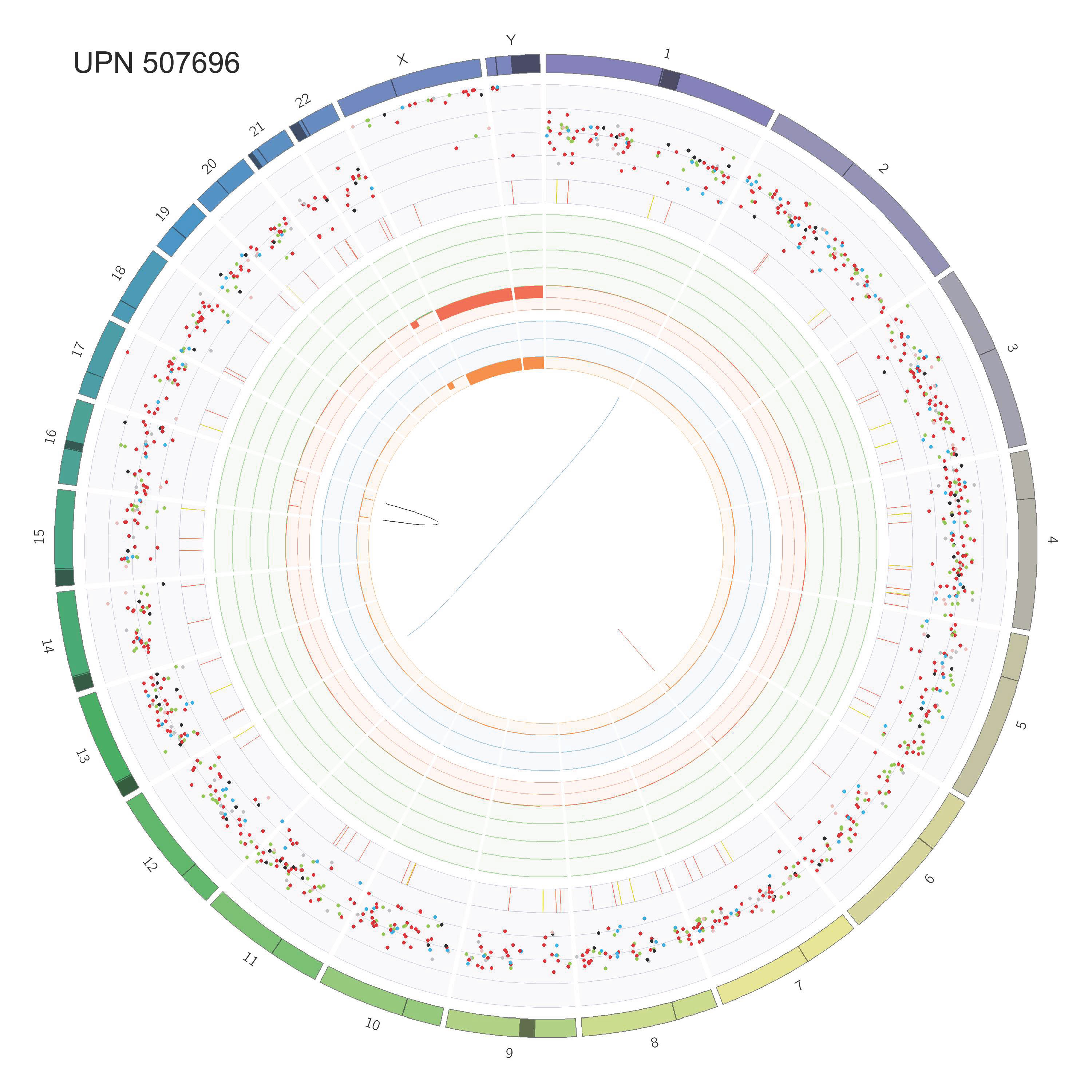

Supplement: Supplement 3 — Supplementary Figure 2. Circos plots [file media-3.zip › Supp_Fig_2_circos_Page_33.jpg]

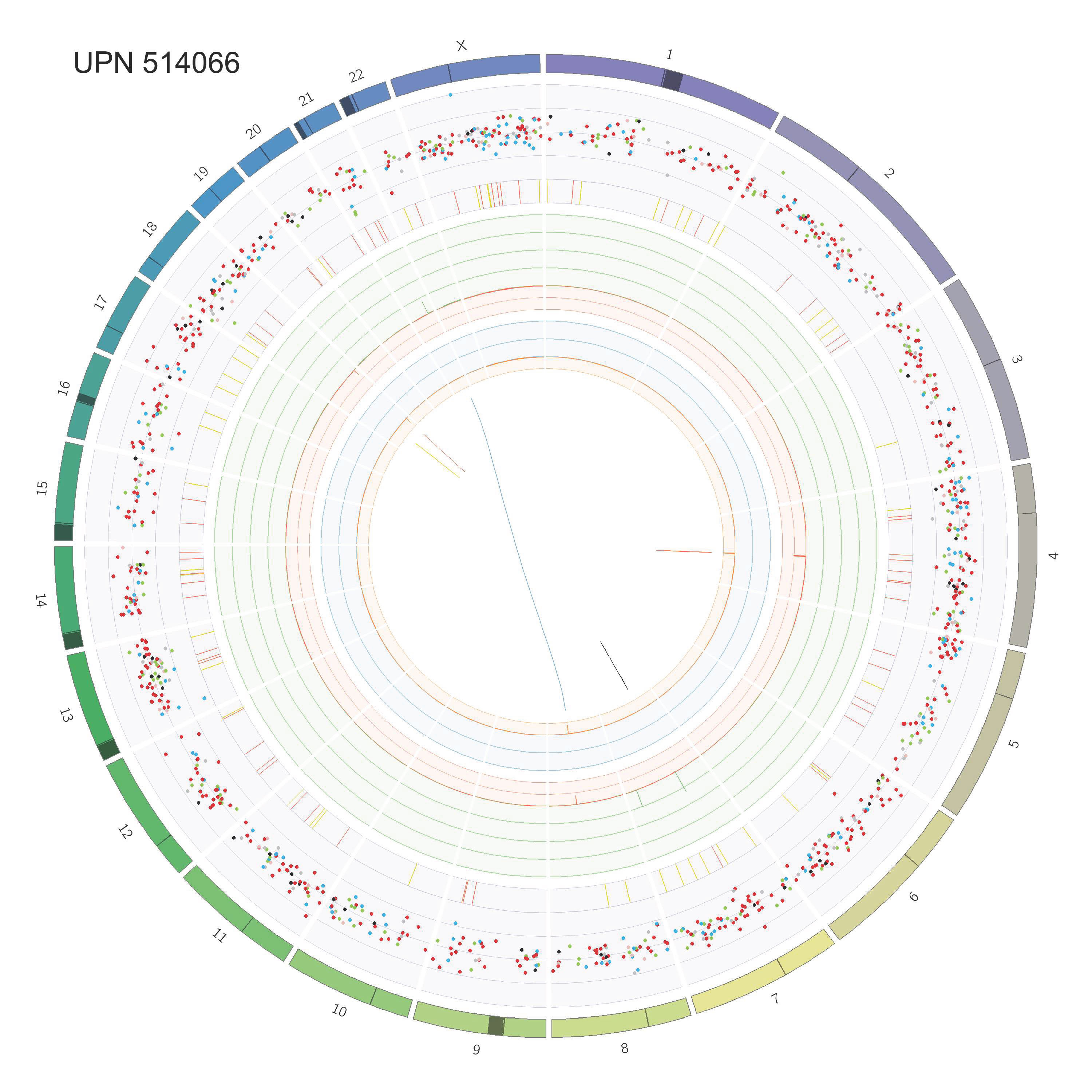

Supplement: Supplement 3 — Supplementary Figure 2. Circos plots [file media-3.zip › Supp_Fig_2_circos_Page_34.jpg]

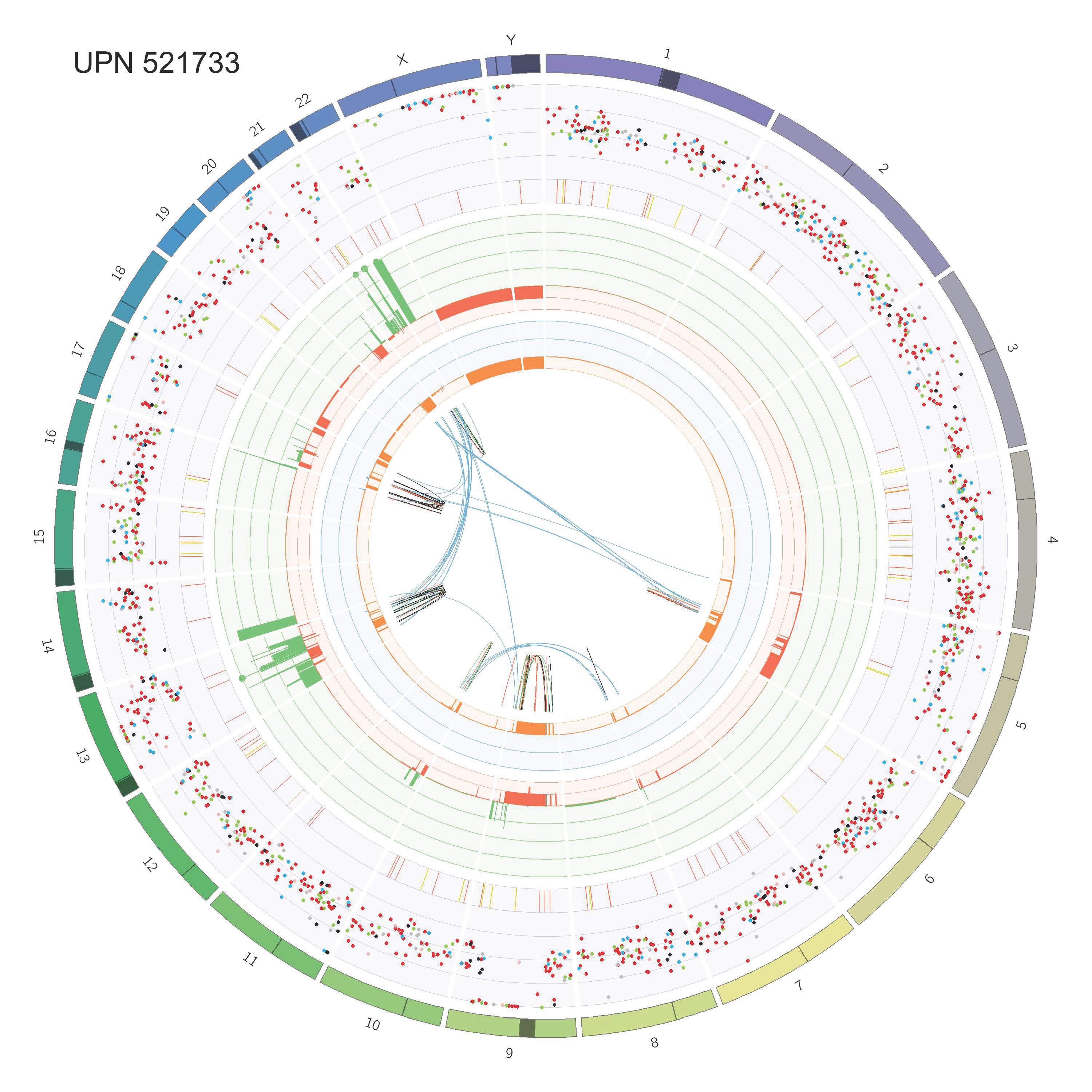

Supplement: Supplement 3 — Supplementary Figure 2. Circos plots [file media-3.zip › Supp_Fig_2_circos_Page_35.jpg]

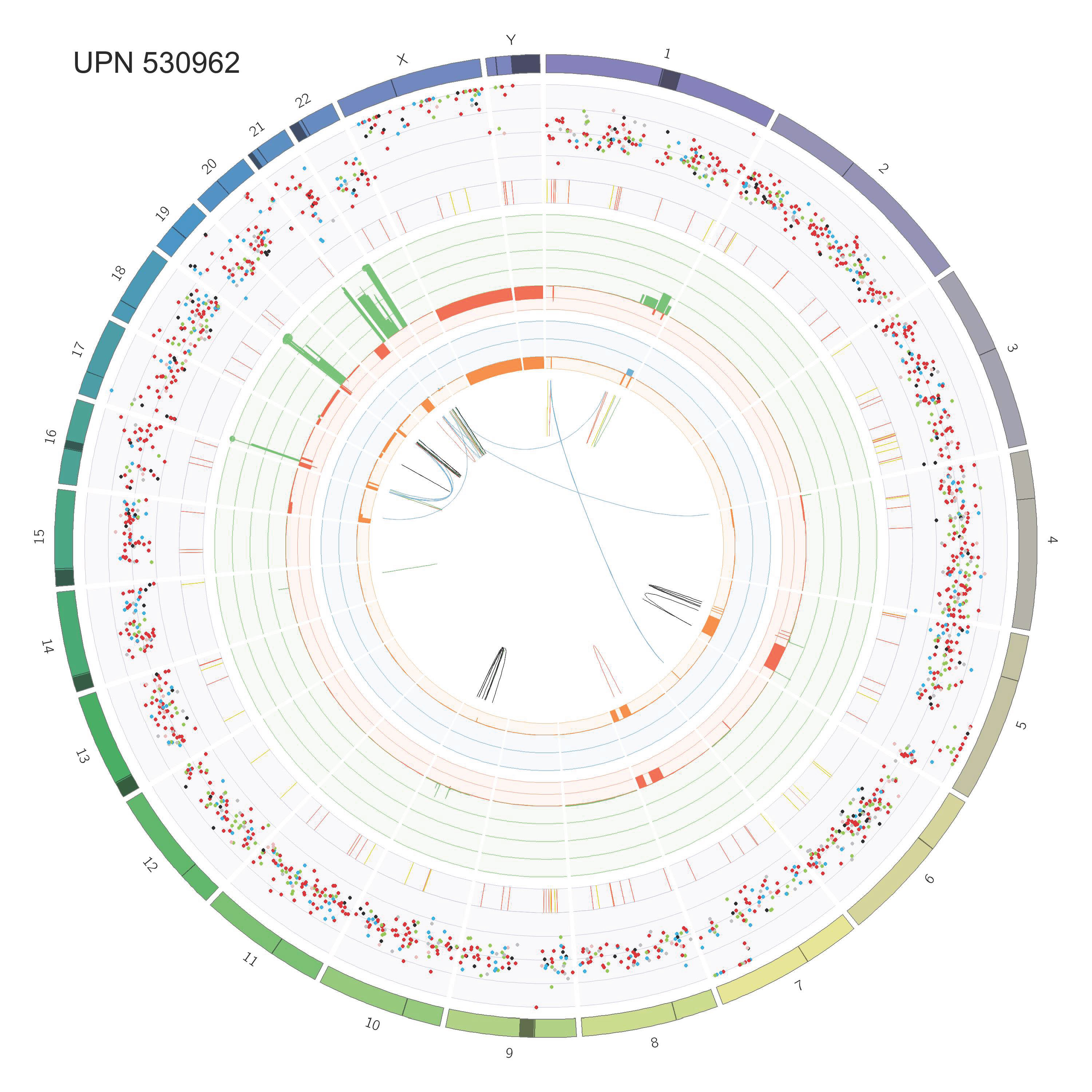

Supplement: Supplement 3 — Supplementary Figure 2. Circos plots [file media-3.zip › Supp_Fig_2_circos_Page_36.jpg]

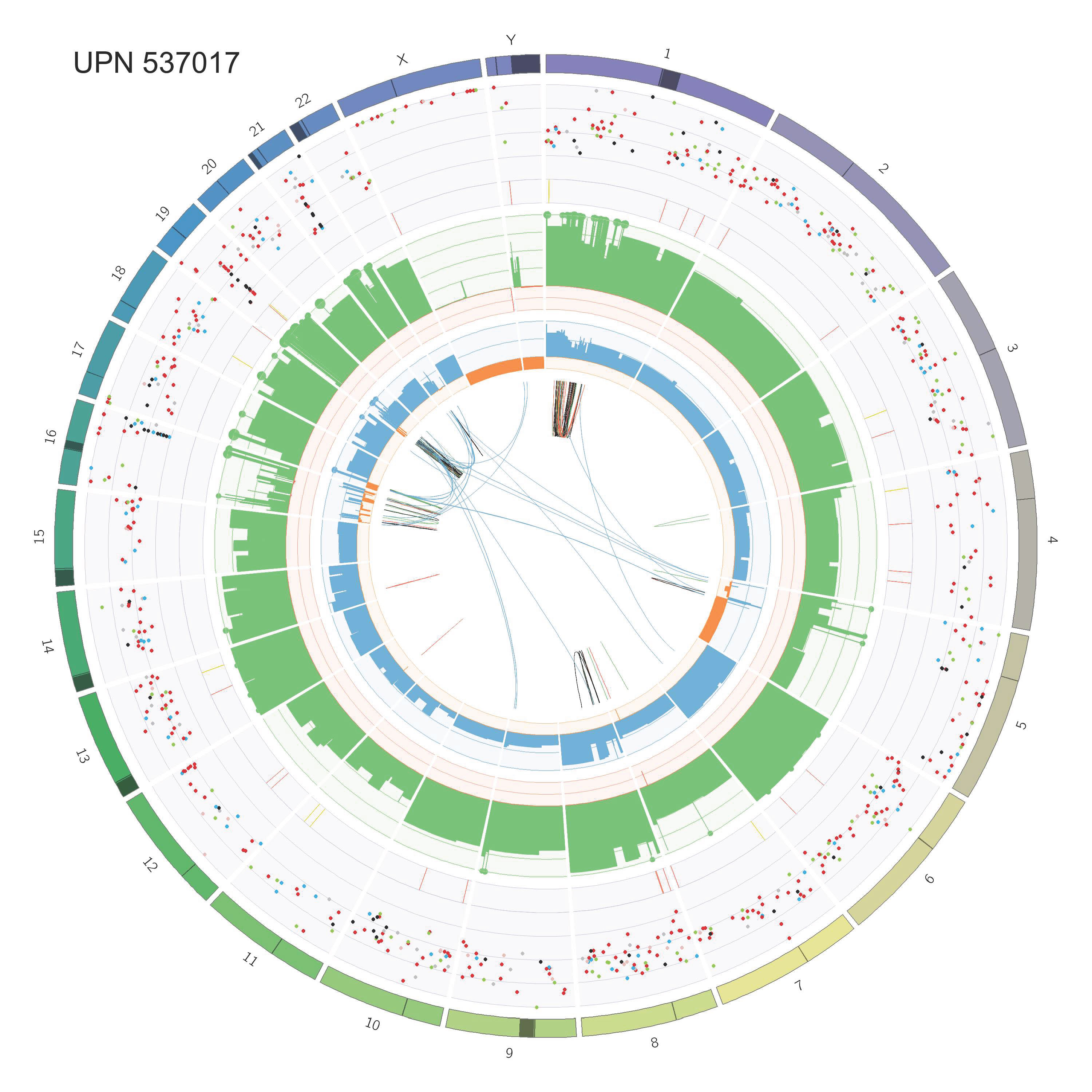

Supplement: Supplement 3 — Supplementary Figure 2. Circos plots [file media-3.zip › Supp_Fig_2_circos_Page_37.jpg]

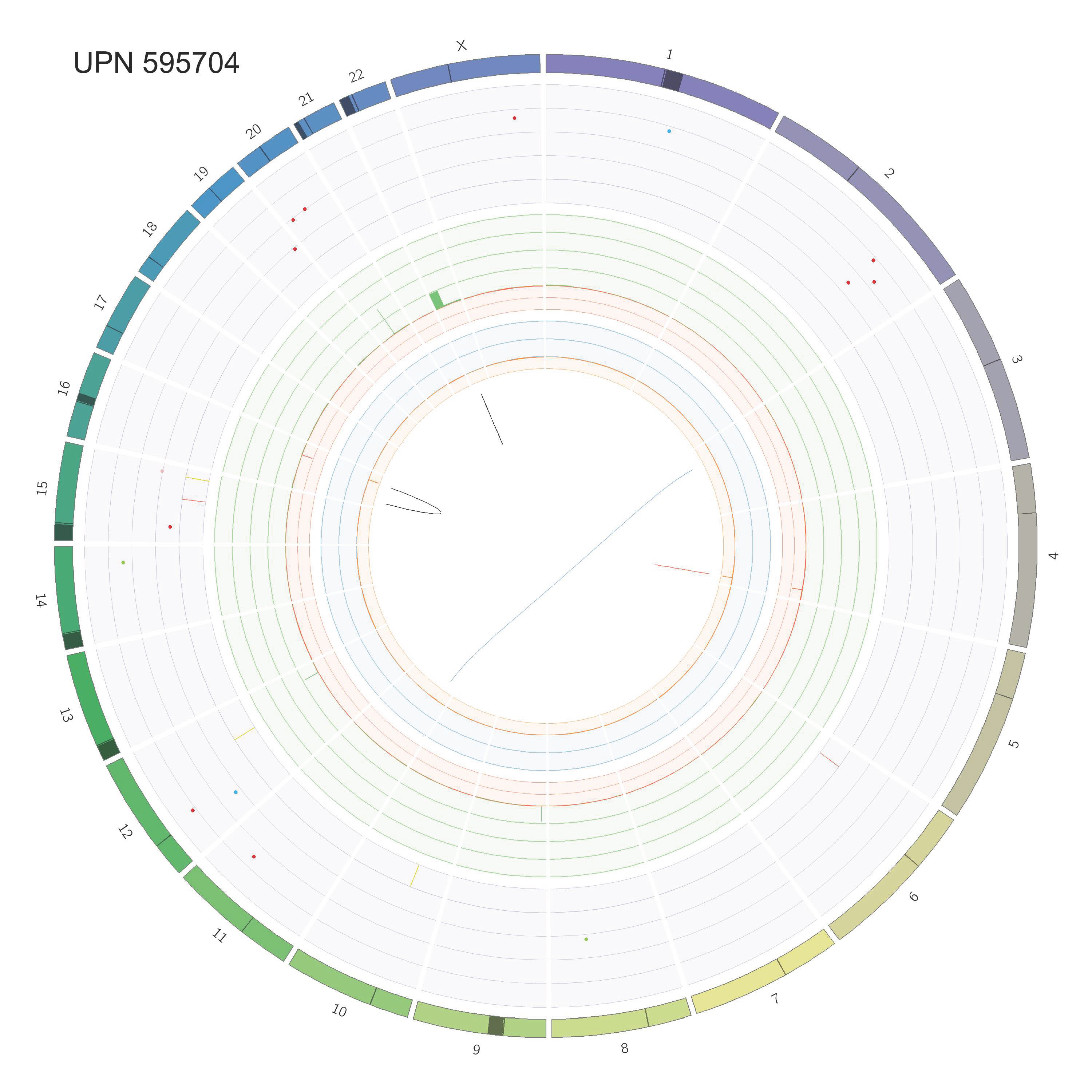

Supplement: Supplement 3 — Supplementary Figure 2. Circos plots [file media-3.zip › Supp_Fig_2_circos_Page_38.jpg]

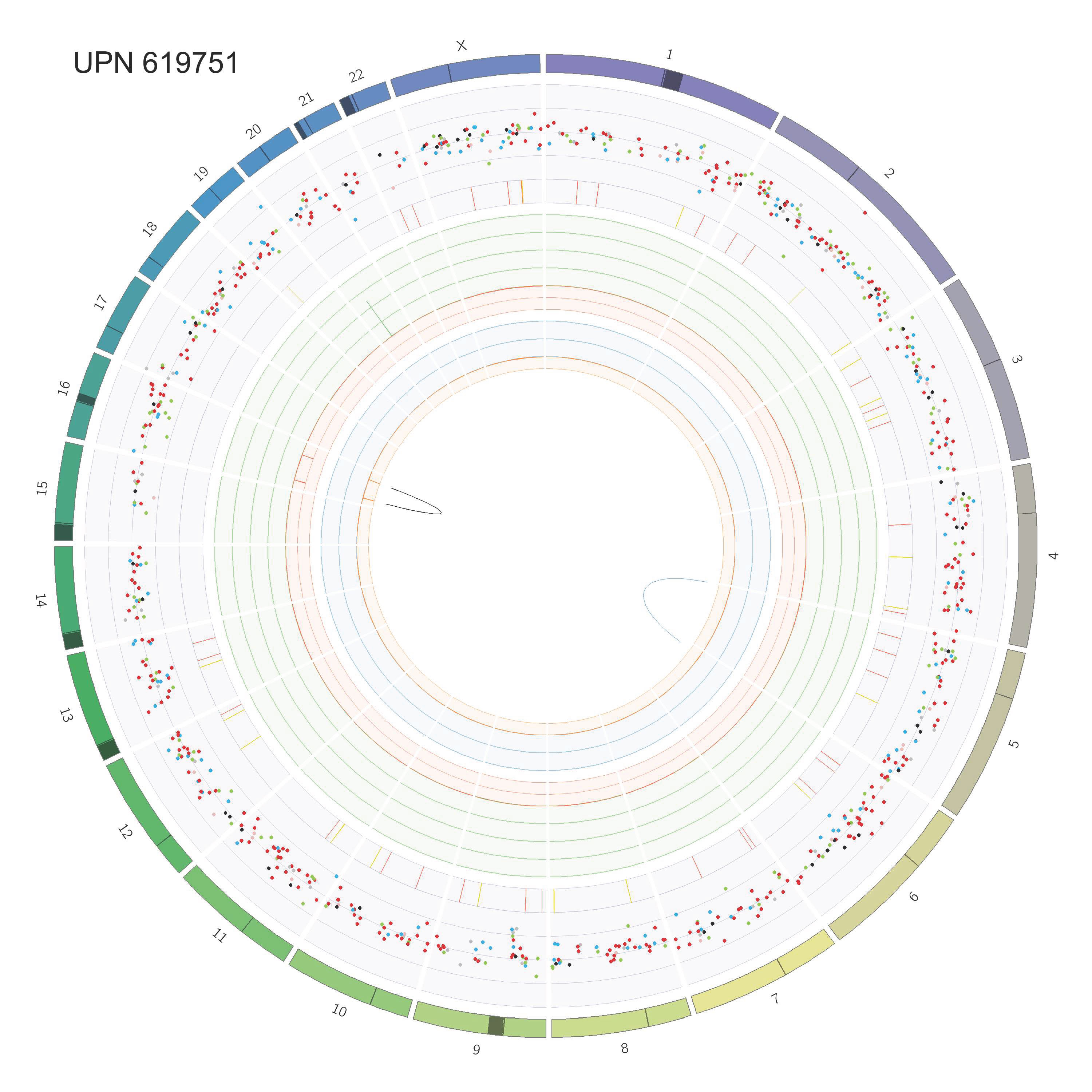

Supplement: Supplement 3 — Supplementary Figure 2. Circos plots [file media-3.zip › Supp_Fig_2_circos_Page_39.jpg]

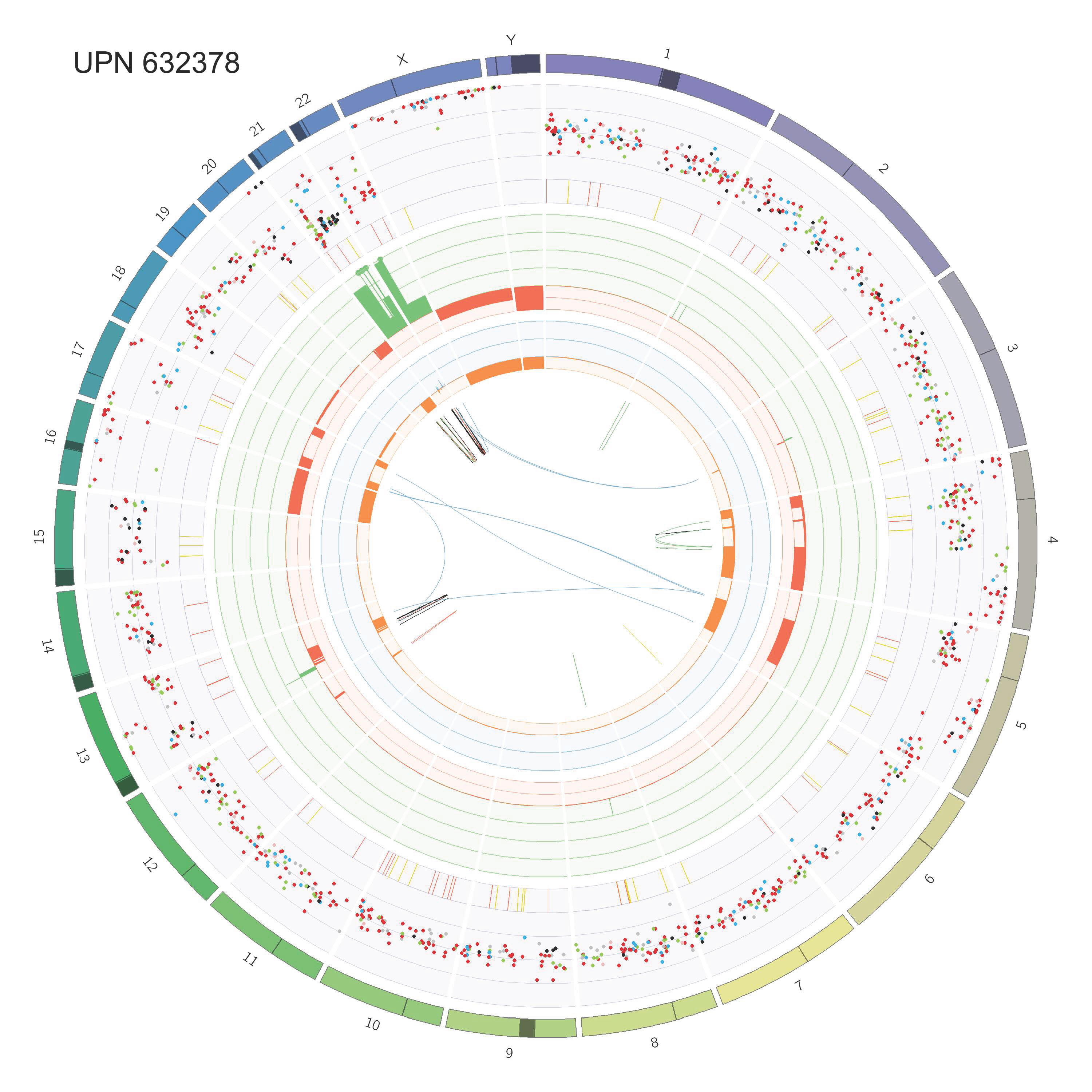

Supplement: Supplement 3 — Supplementary Figure 2. Circos plots [file media-3.zip › Supp_Fig_2_circos_Page_40.jpg]

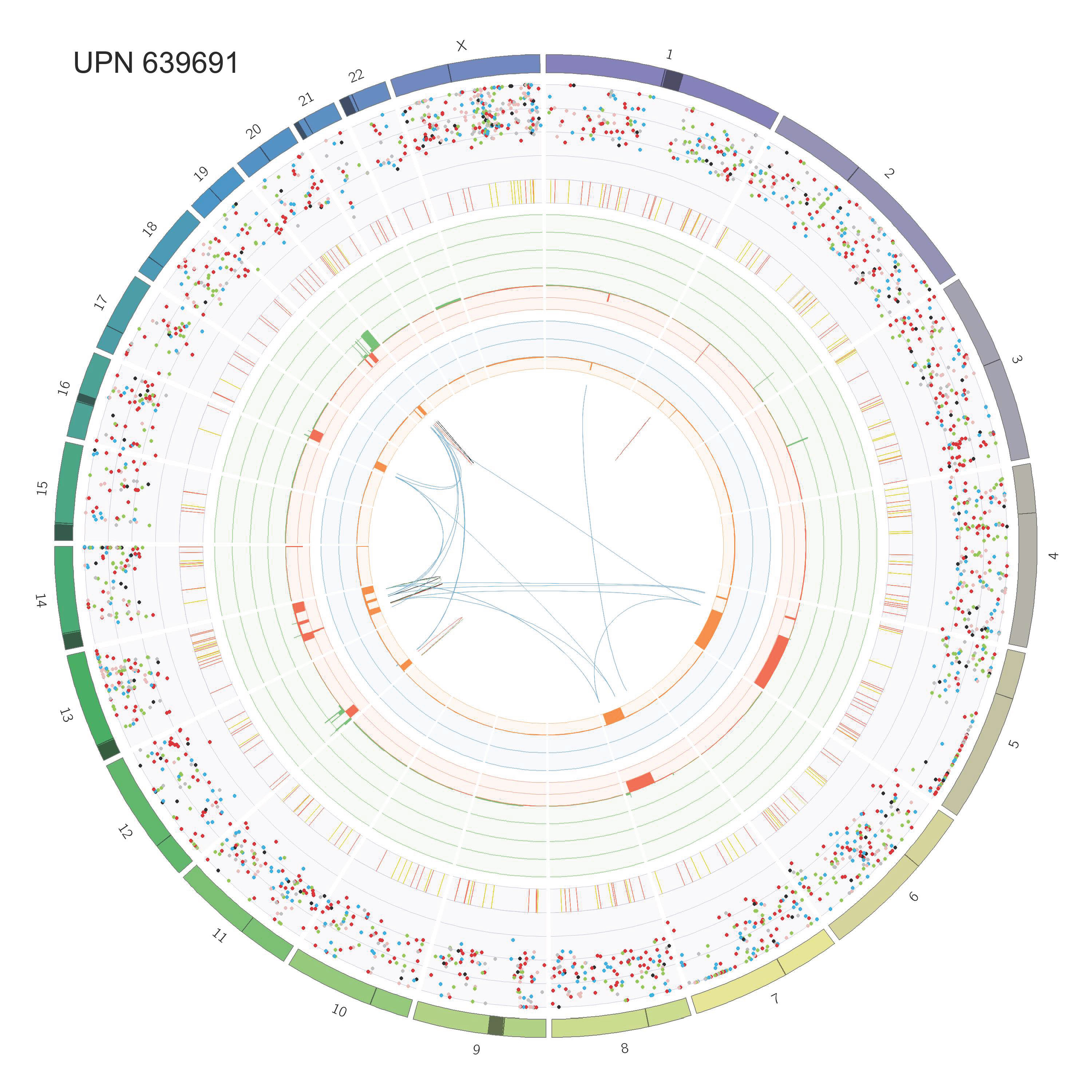

Supplement: Supplement 3 — Supplementary Figure 2. Circos plots [file media-3.zip › Supp_Fig_2_circos_Page_41.jpg]

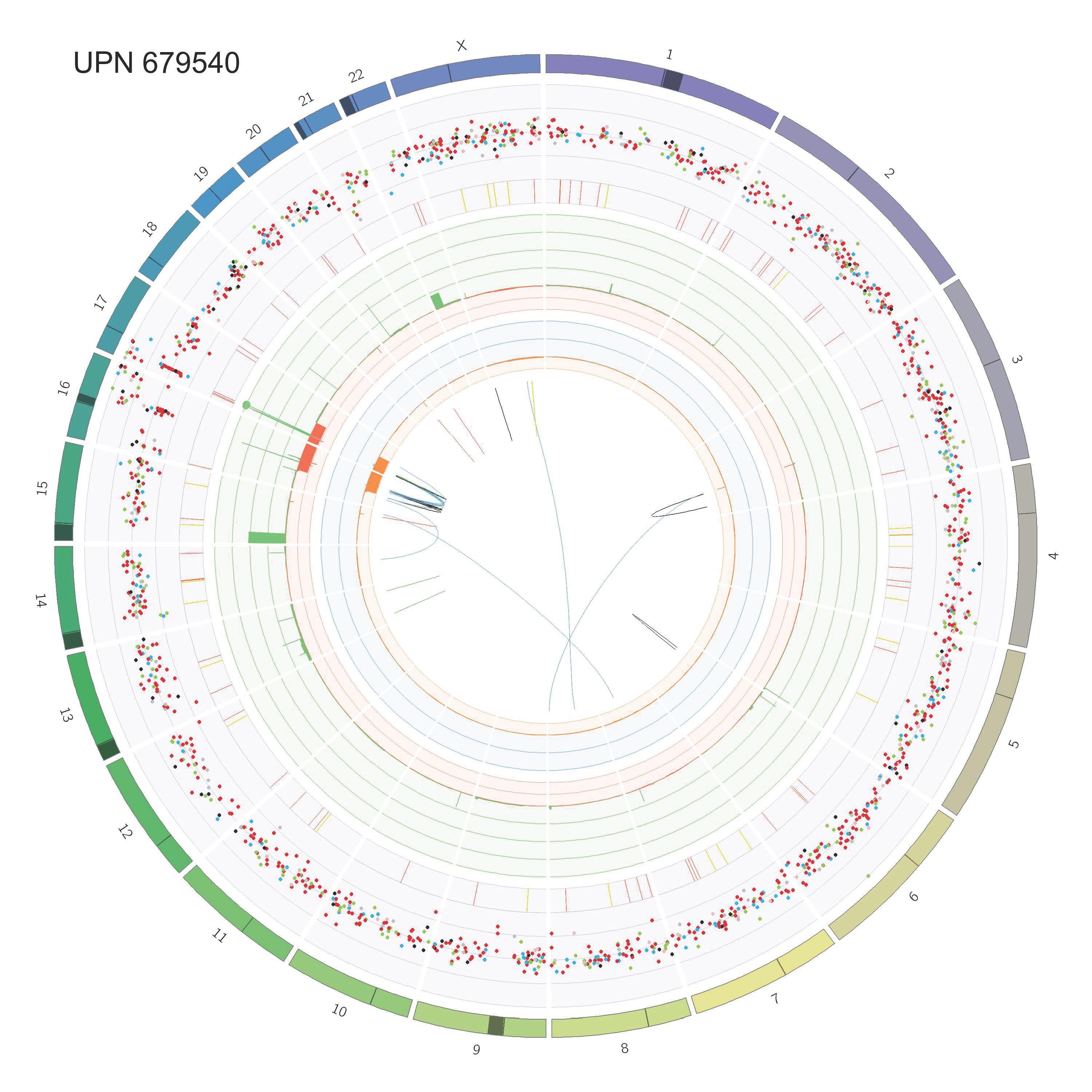

Supplement: Supplement 3 — Supplementary Figure 2. Circos plots [file media-3.zip › Supp_Fig_2_circos_Page_42.jpg]

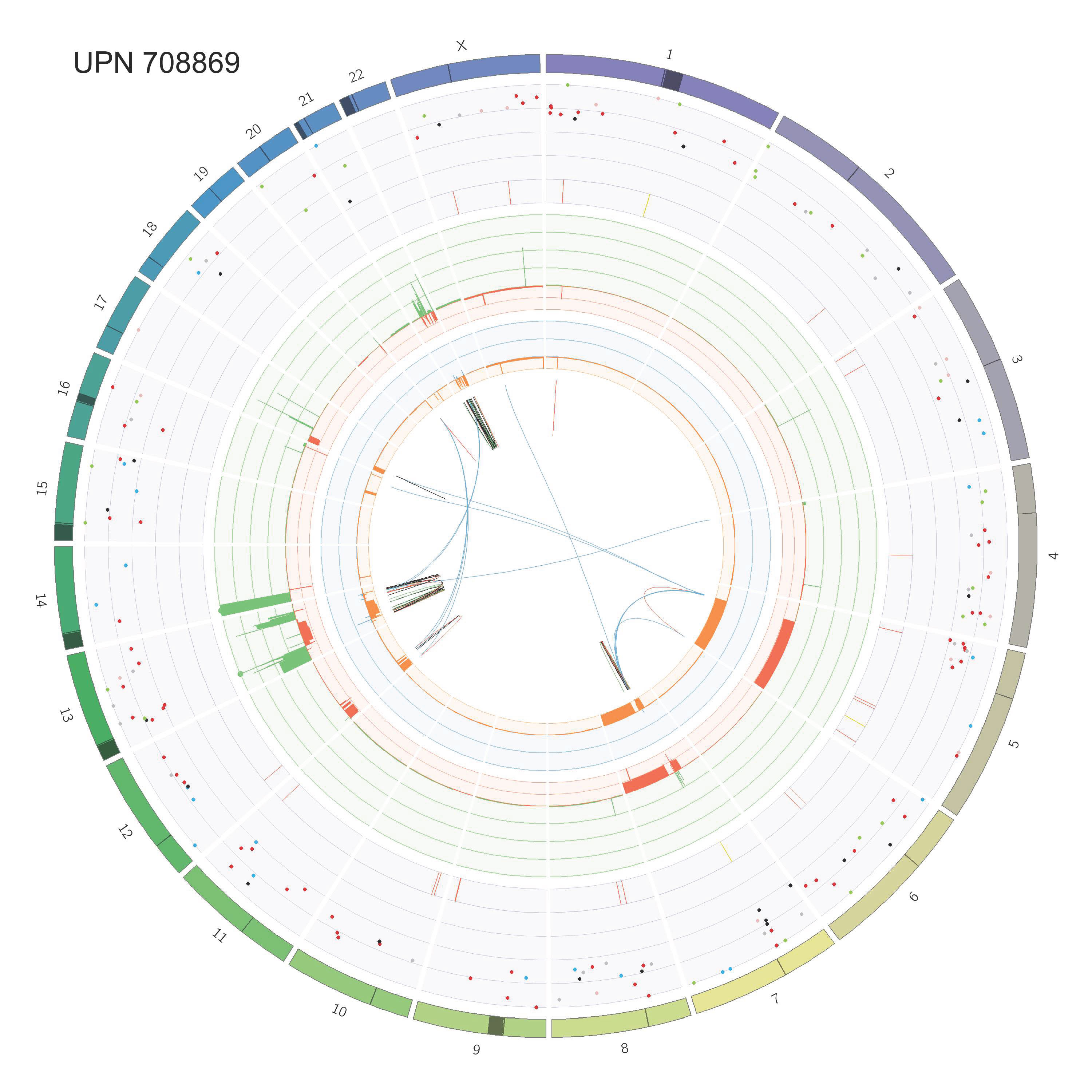

Supplement: Supplement 3 — Supplementary Figure 2. Circos plots [file media-3.zip › Supp_Fig_2_circos_Page_43.jpg]

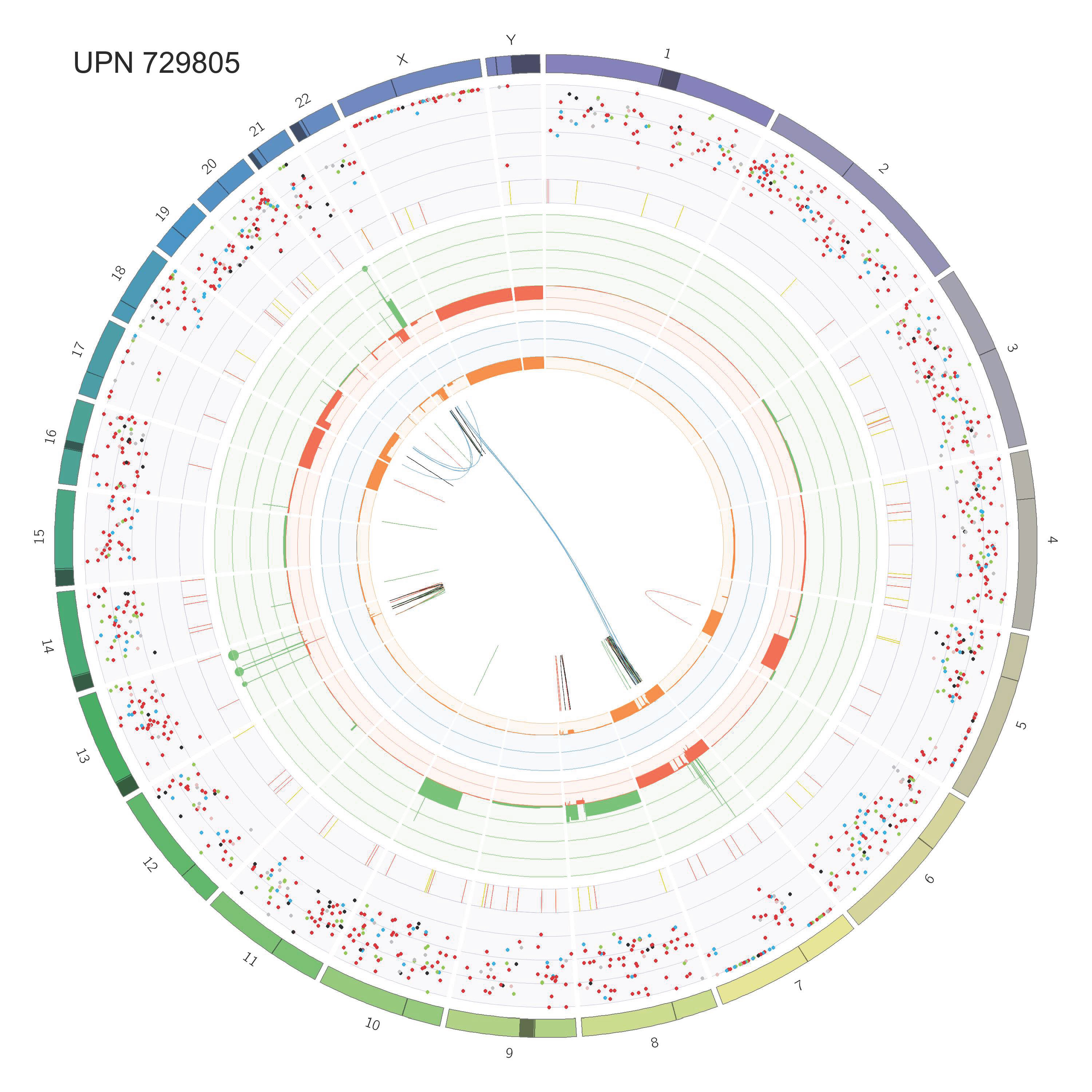

Supplement: Supplement 3 — Supplementary Figure 2. Circos plots [file media-3.zip › Supp_Fig_2_circos_Page_44.jpg]

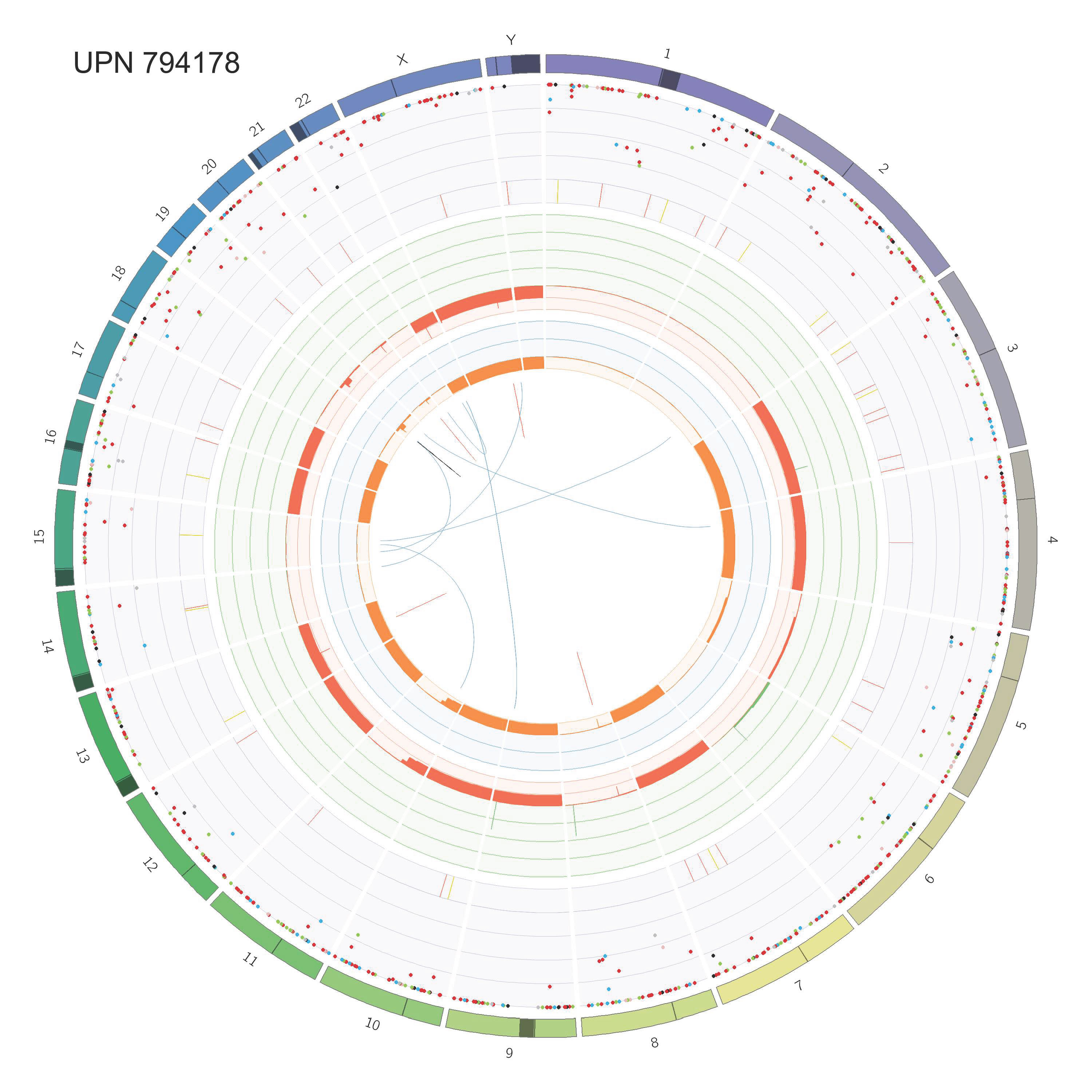

Supplement: Supplement 3 — Supplementary Figure 2. Circos plots [file media-3.zip › Supp_Fig_2_circos_Page_45.jpg]

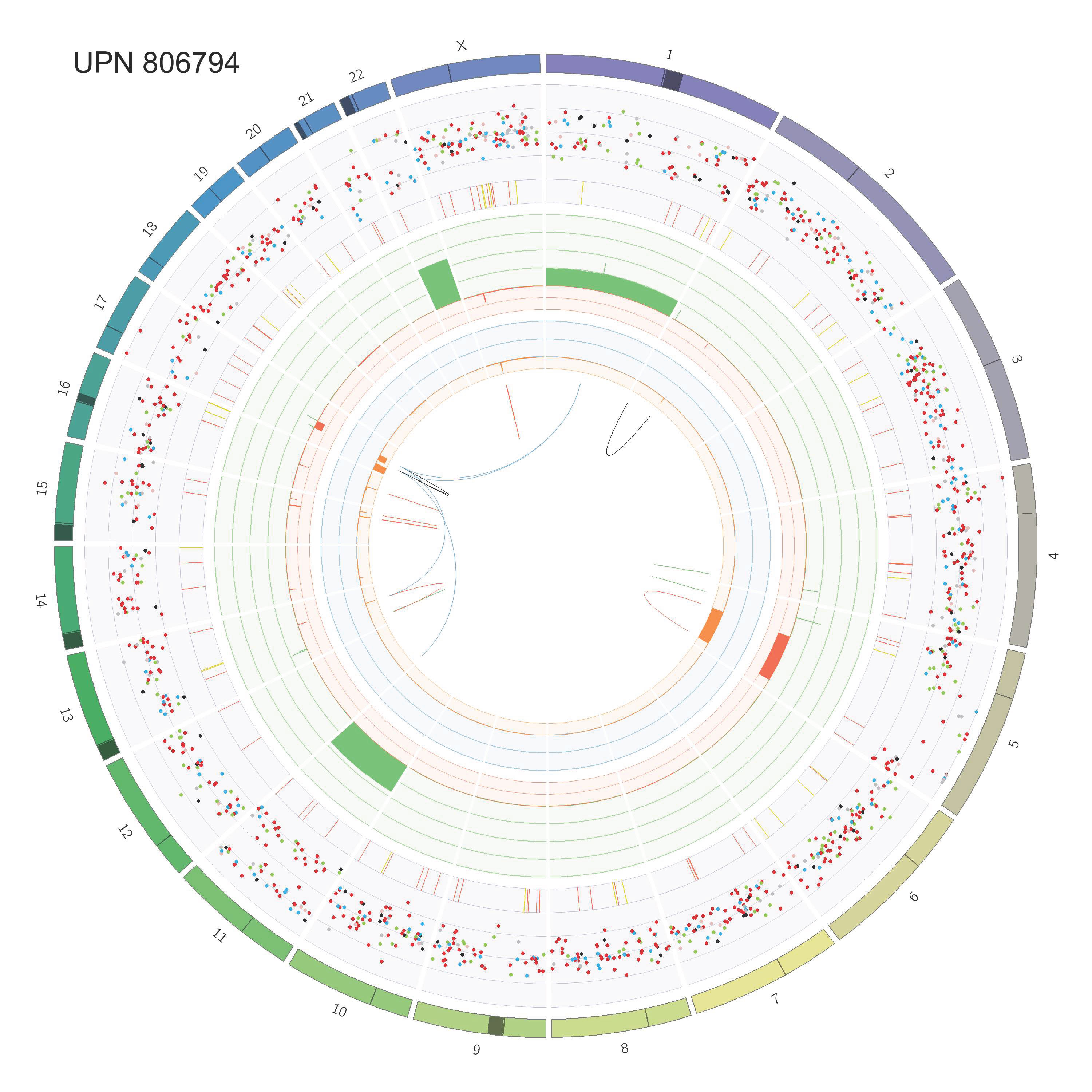

Supplement: Supplement 3 — Supplementary Figure 2. Circos plots [file media-3.zip › Supp_Fig_2_circos_Page_46.jpg]

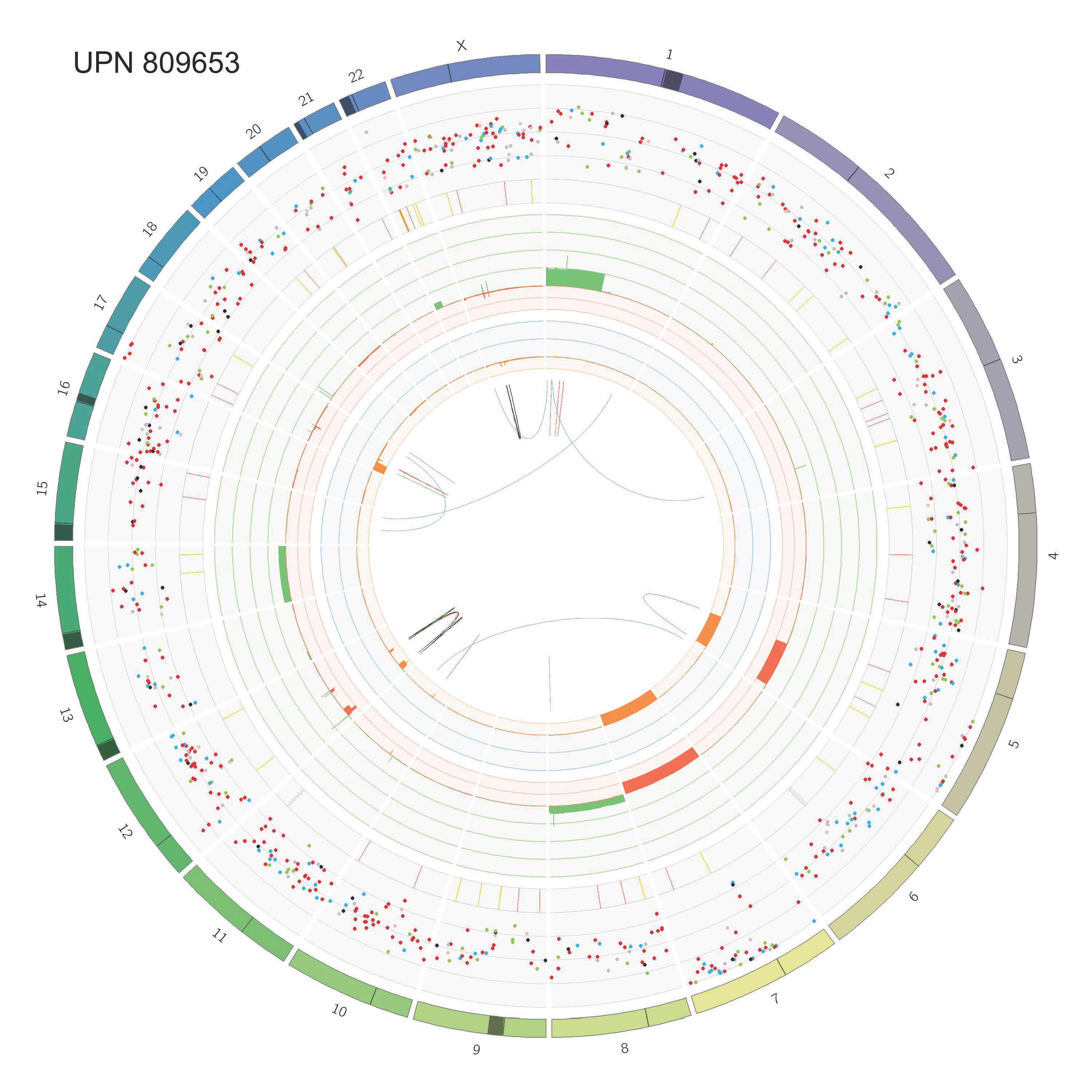

Supplement: Supplement 3 — Supplementary Figure 2. Circos plots [file media-3.zip › Supp_Fig_2_circos_Page_47.jpg]

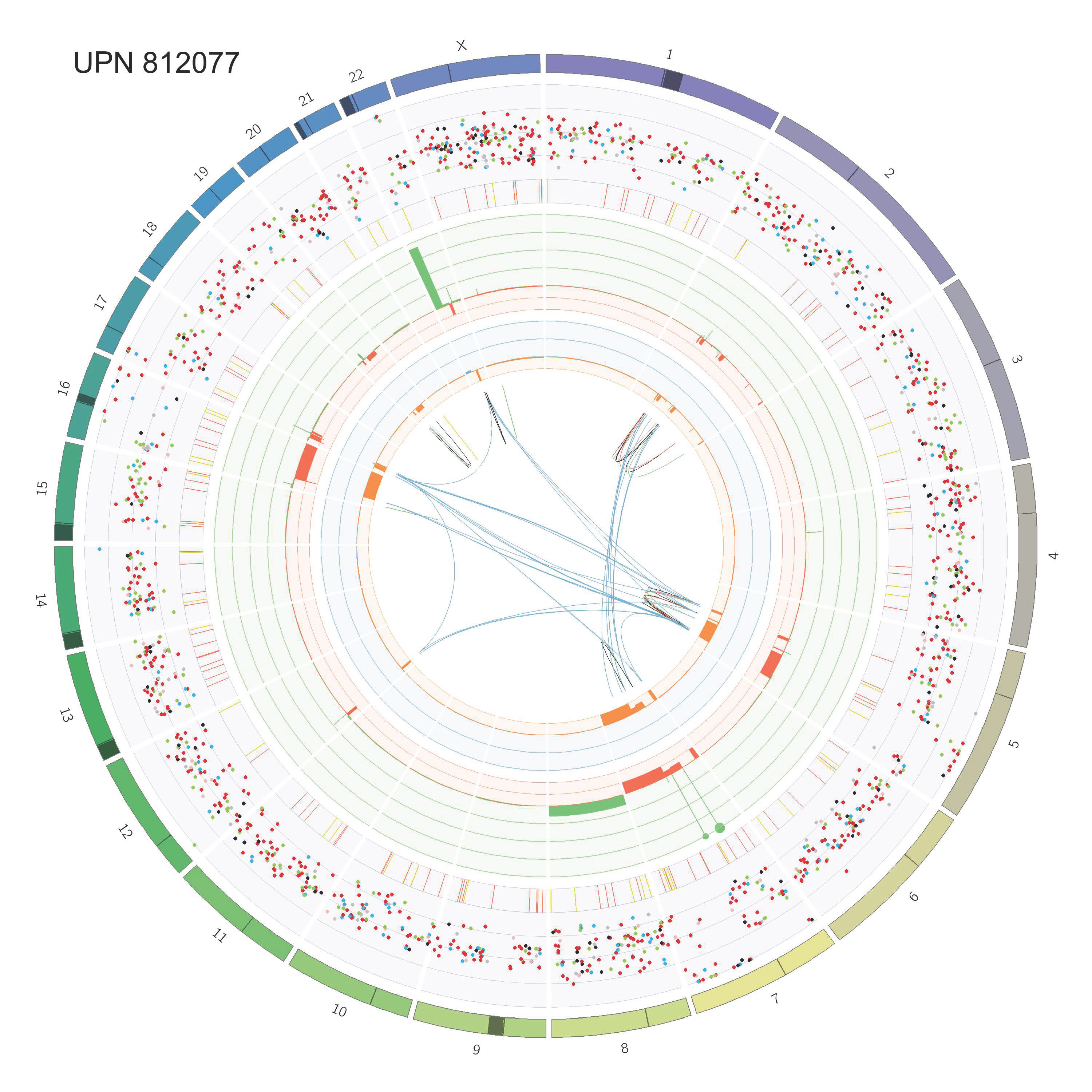

Supplement: Supplement 3 — Supplementary Figure 2. Circos plots [file media-3.zip › Supp_Fig_2_circos_Page_48.jpg]

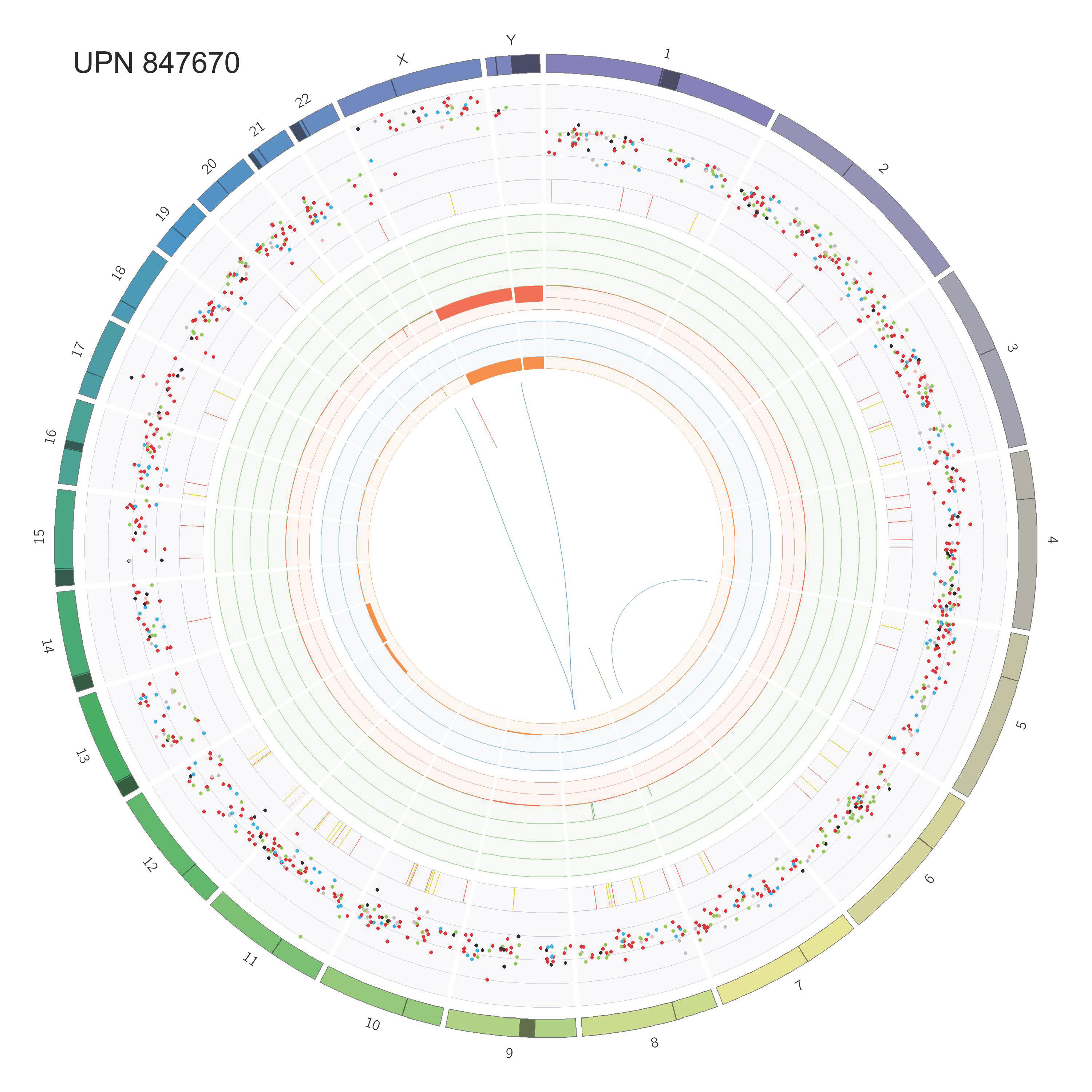

Supplement: Supplement 3 — Supplementary Figure 2. Circos plots [file media-3.zip › Supp_Fig_2_circos_Page_49.jpg]

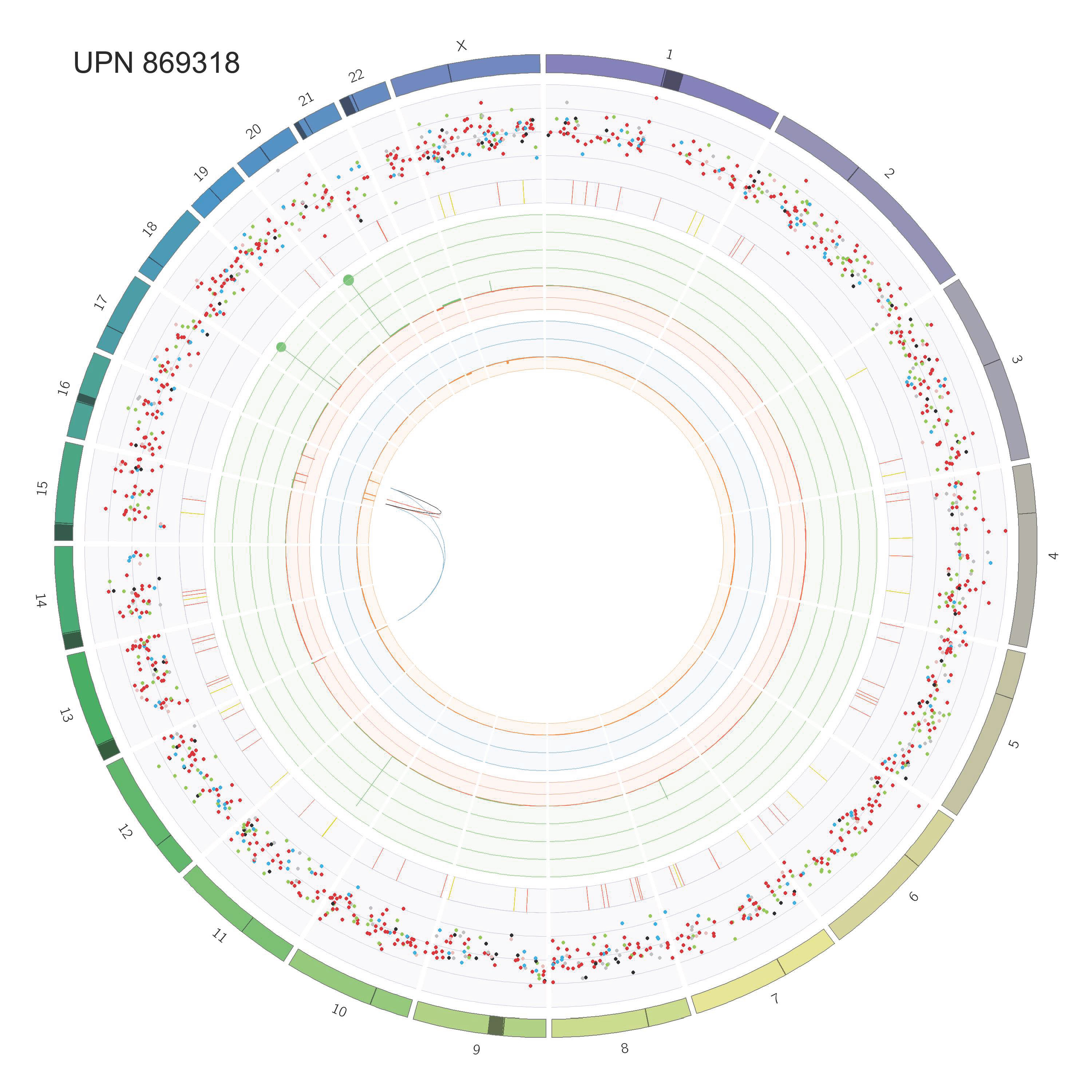

Supplement: Supplement 3 — Supplementary Figure 2. Circos plots [file media-3.zip › Supp_Fig_2_circos_Page_50.jpg]

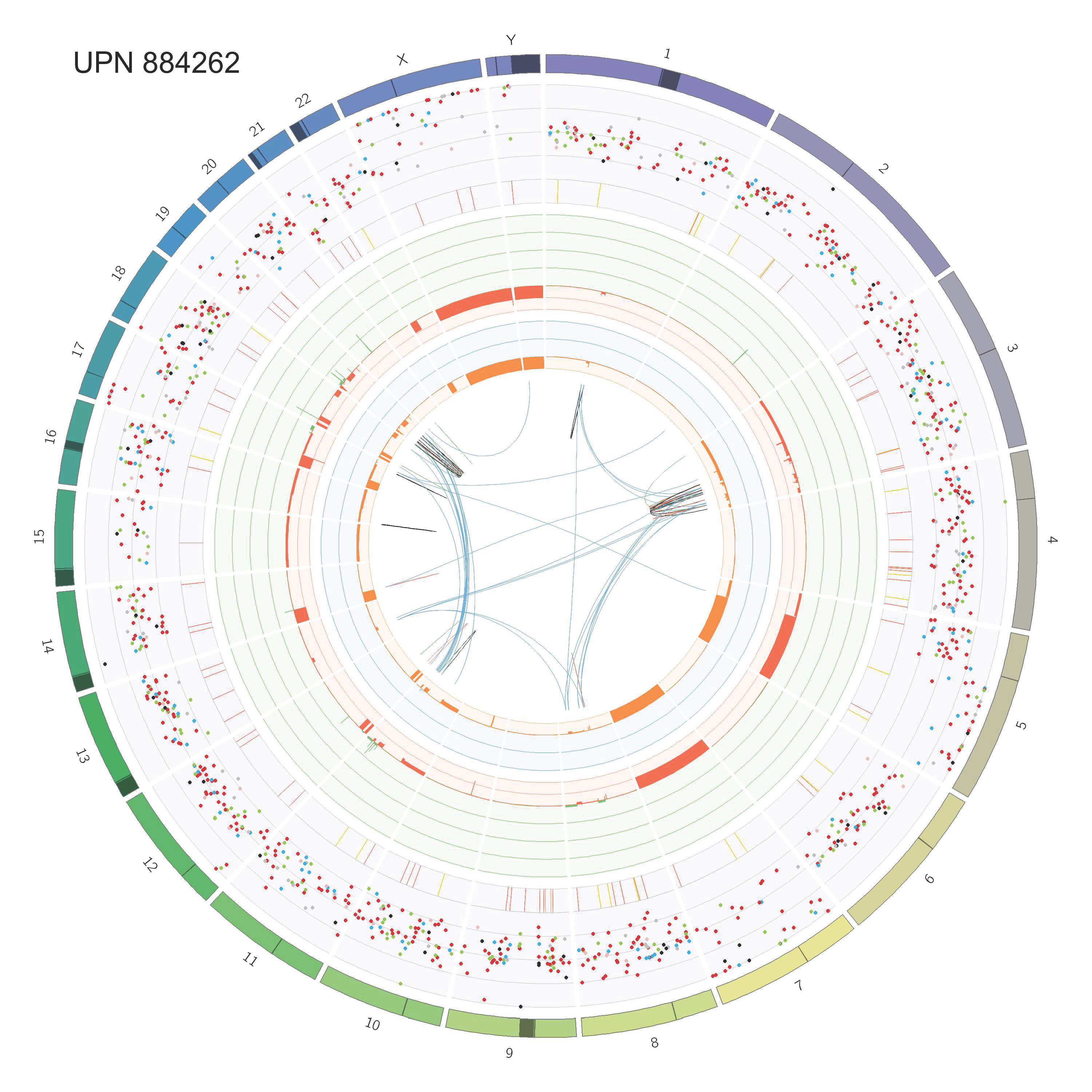

Supplement: Supplement 3 — Supplementary Figure 2. Circos plots [file media-3.zip › Supp_Fig_2_circos_Page_51.jpg]

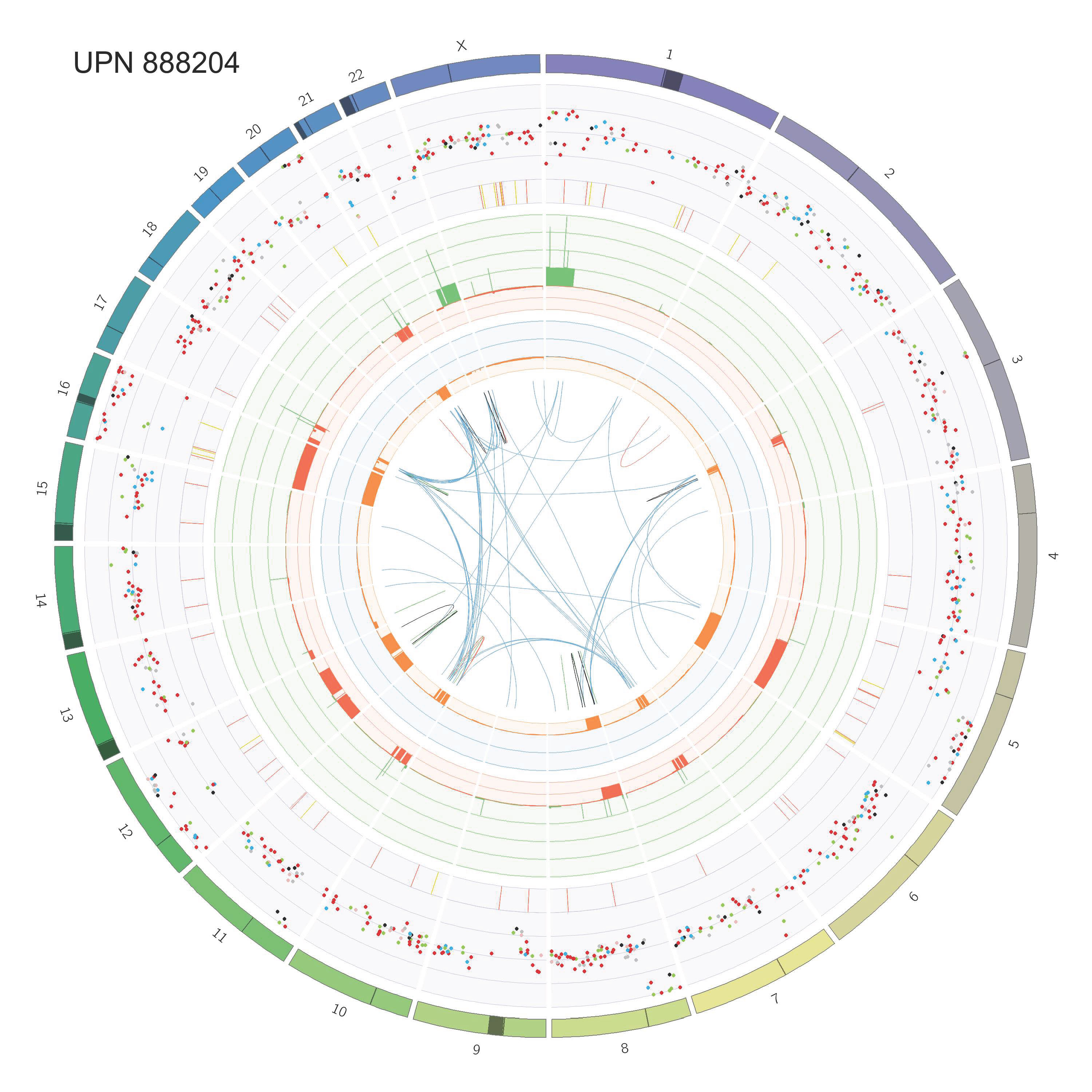

Supplement: Supplement 3 — Supplementary Figure 2. Circos plots [file media-3.zip › Supp_Fig_2_circos_Page_52.jpg]

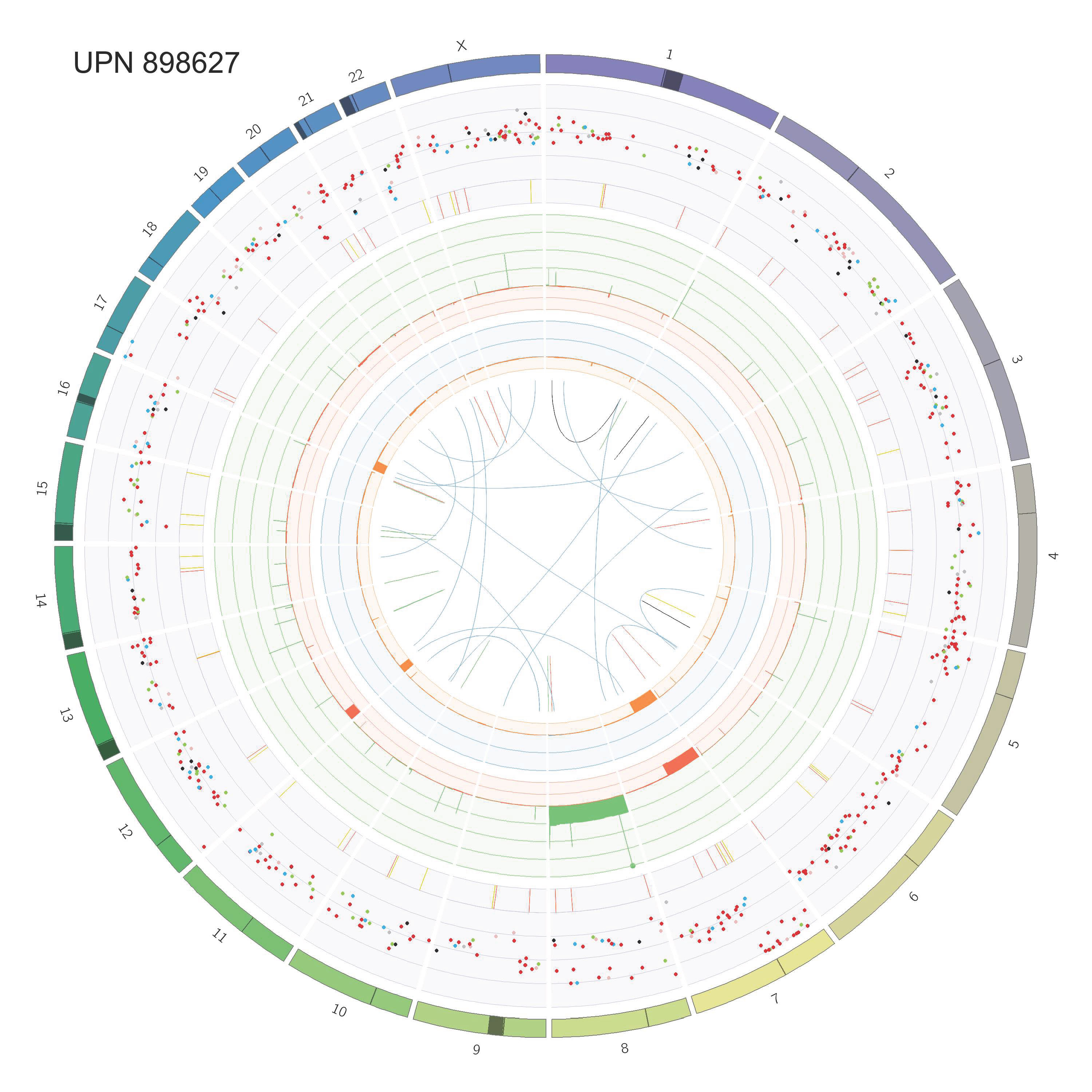

Supplement: Supplement 3 — Supplementary Figure 2. Circos plots [file media-3.zip › Supp_Fig_2_circos_Page_53.jpg]

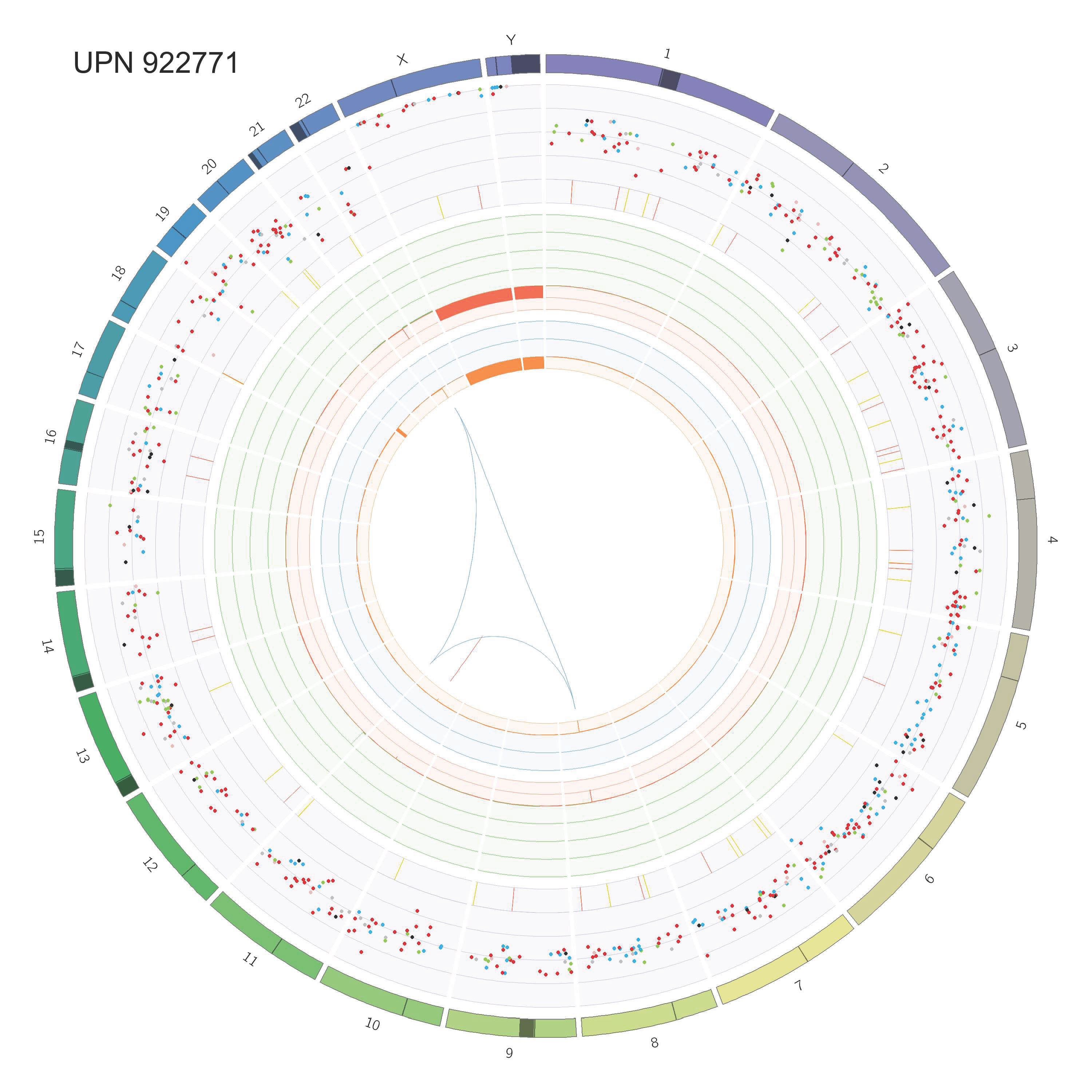

Supplement: Supplement 3 — Supplementary Figure 2. Circos plots [file media-3.zip › Supp_Fig_2_circos_Page_54.jpg]

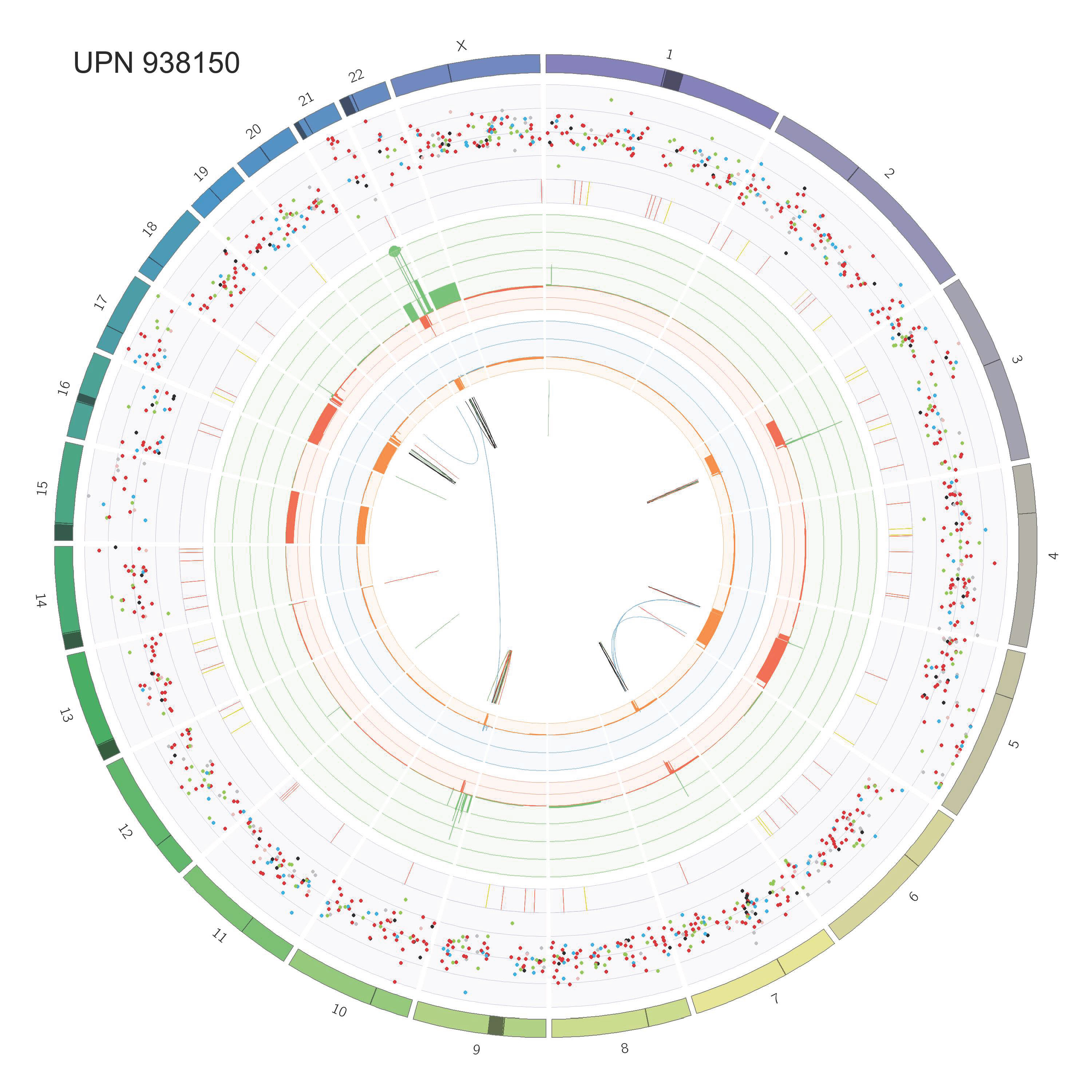

Supplement: Supplement 3 — Supplementary Figure 2. Circos plots [file media-3.zip › Supp_Fig_2_circos_Page_55.jpg]

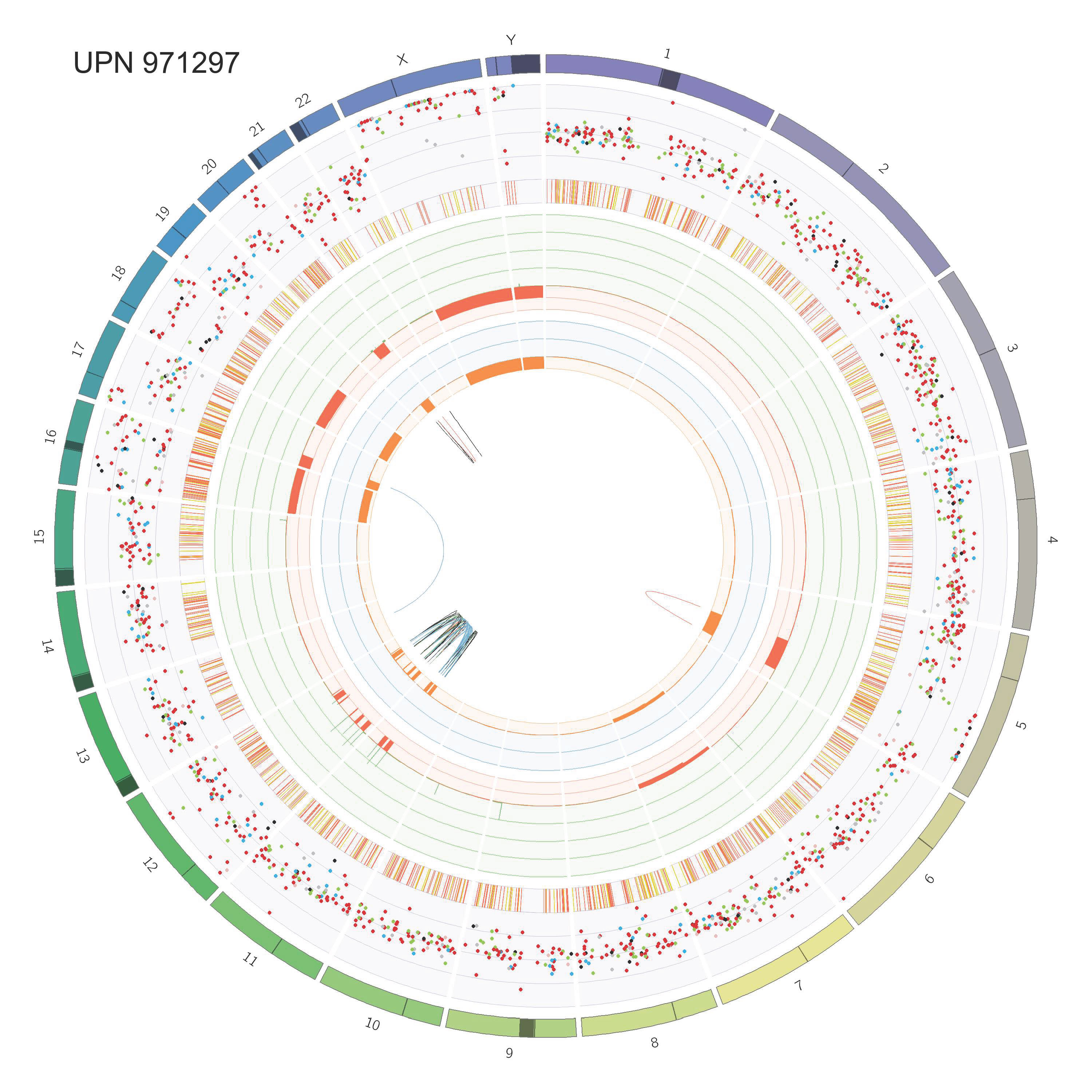

Supplement: Supplement 3 — Supplementary Figure 2. Circos plots [file media-3.zip › Supp_Fig_2_circos_Page_56.jpg]

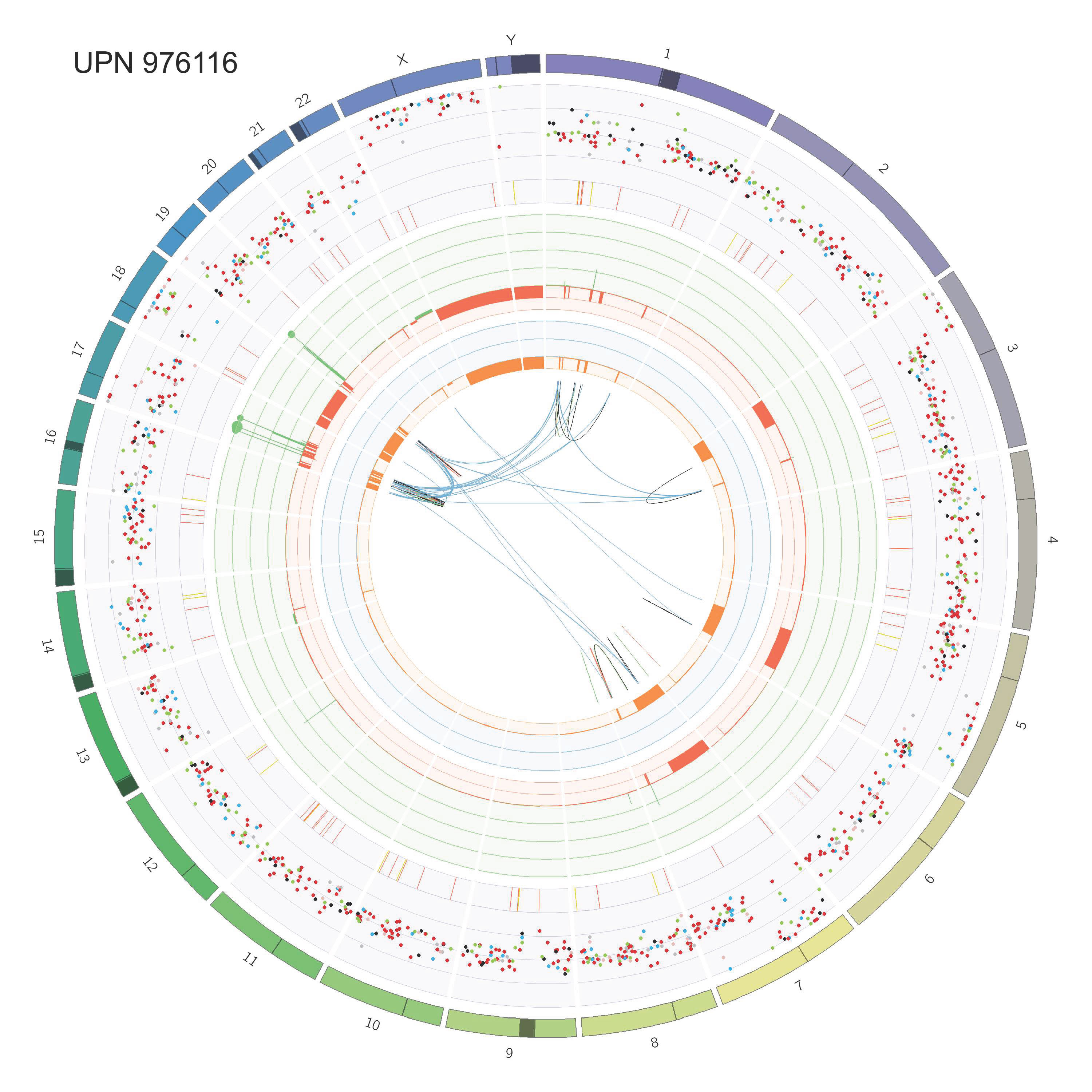

Supplement: Supplement 3 — Supplementary Figure 2. Circos plots [file media-3.zip › Supp_Fig_2_circos_Page_57.jpg]

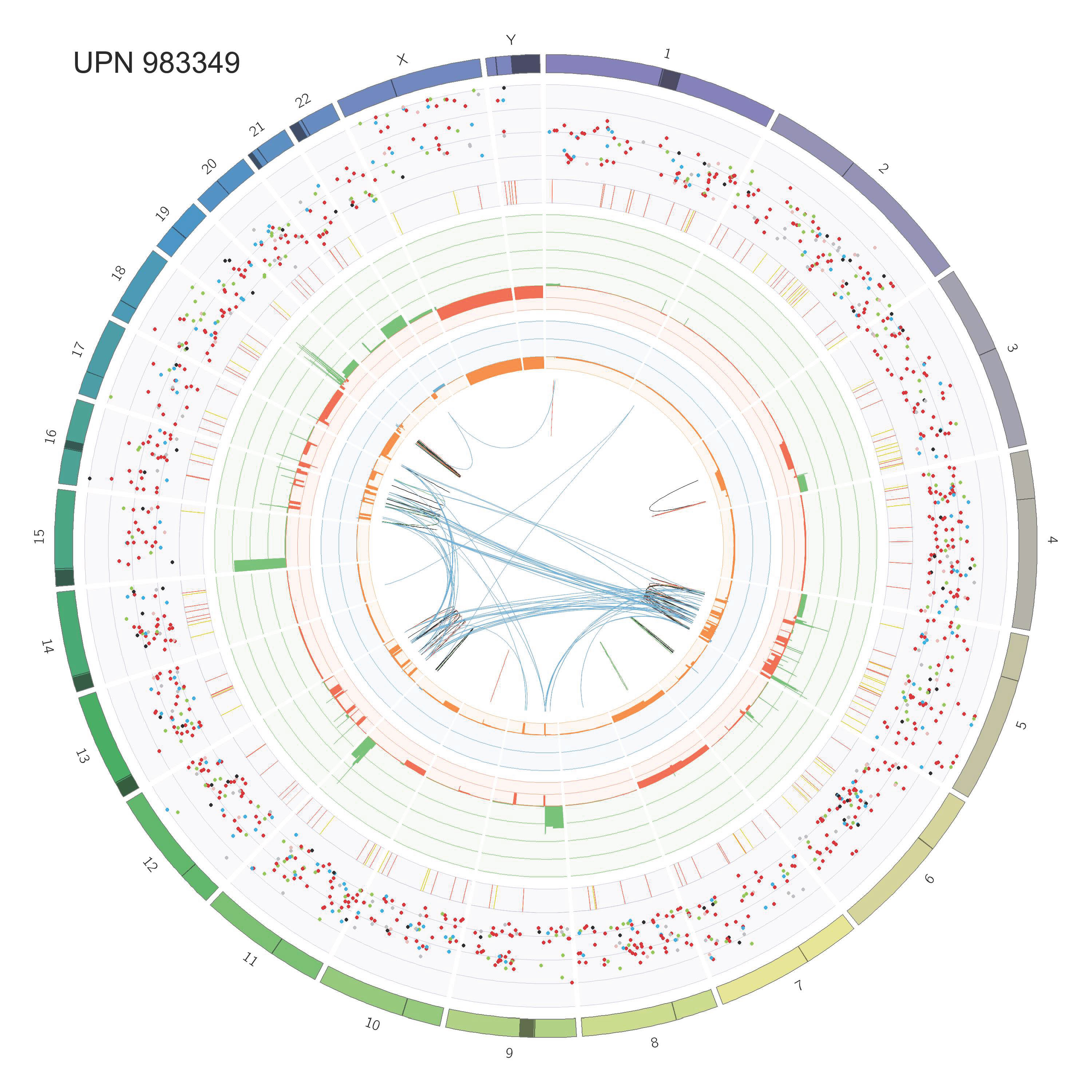

Supplement: Supplement 3 — Supplementary Figure 2. Circos plots [file media-3.zip › Supp_Fig_2_circos_Page_58.jpg]

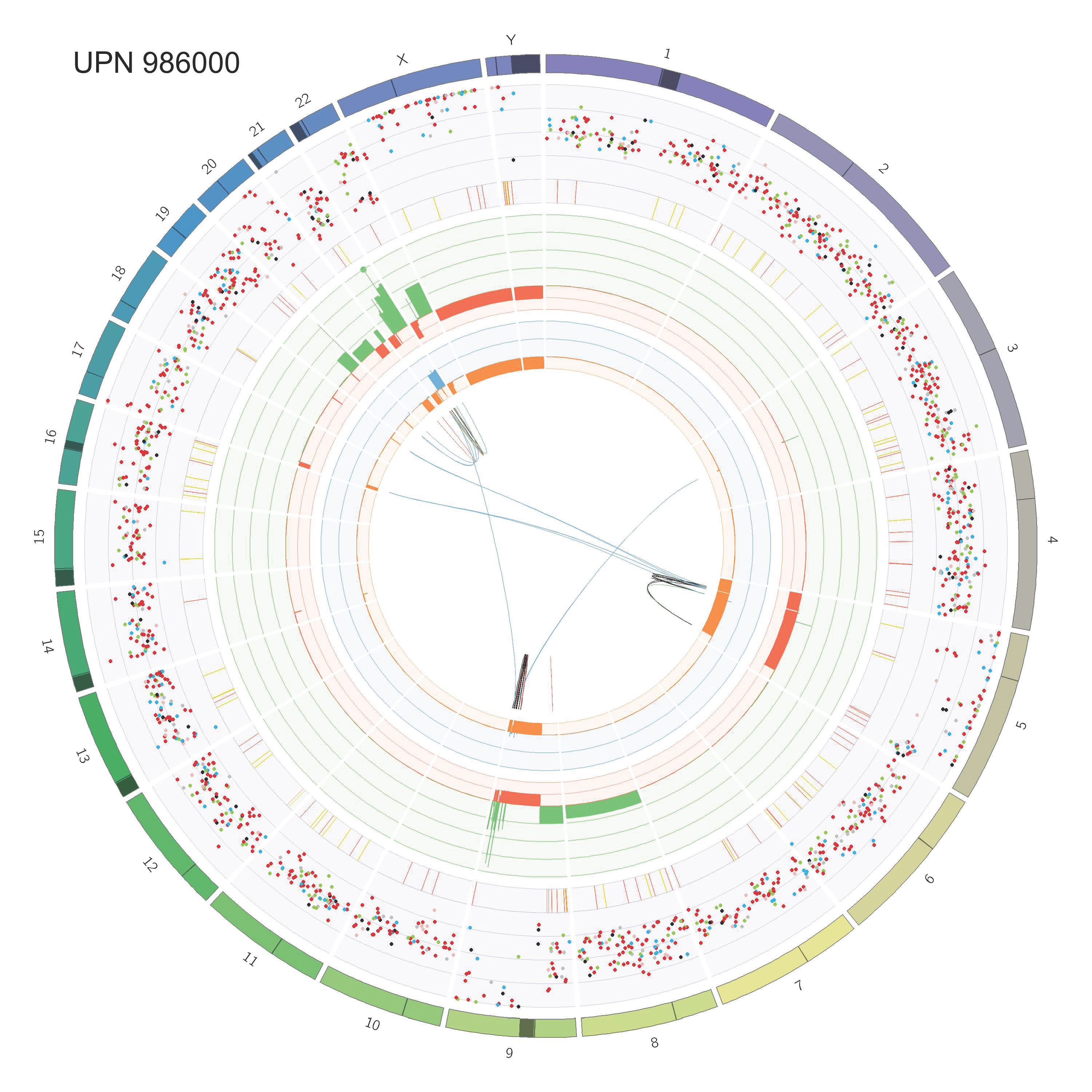

Supplement: Supplement 3 — Supplementary Figure 2. Circos plots [file media-3.zip › Supp_Fig_2_circos_Page_59.jpg]

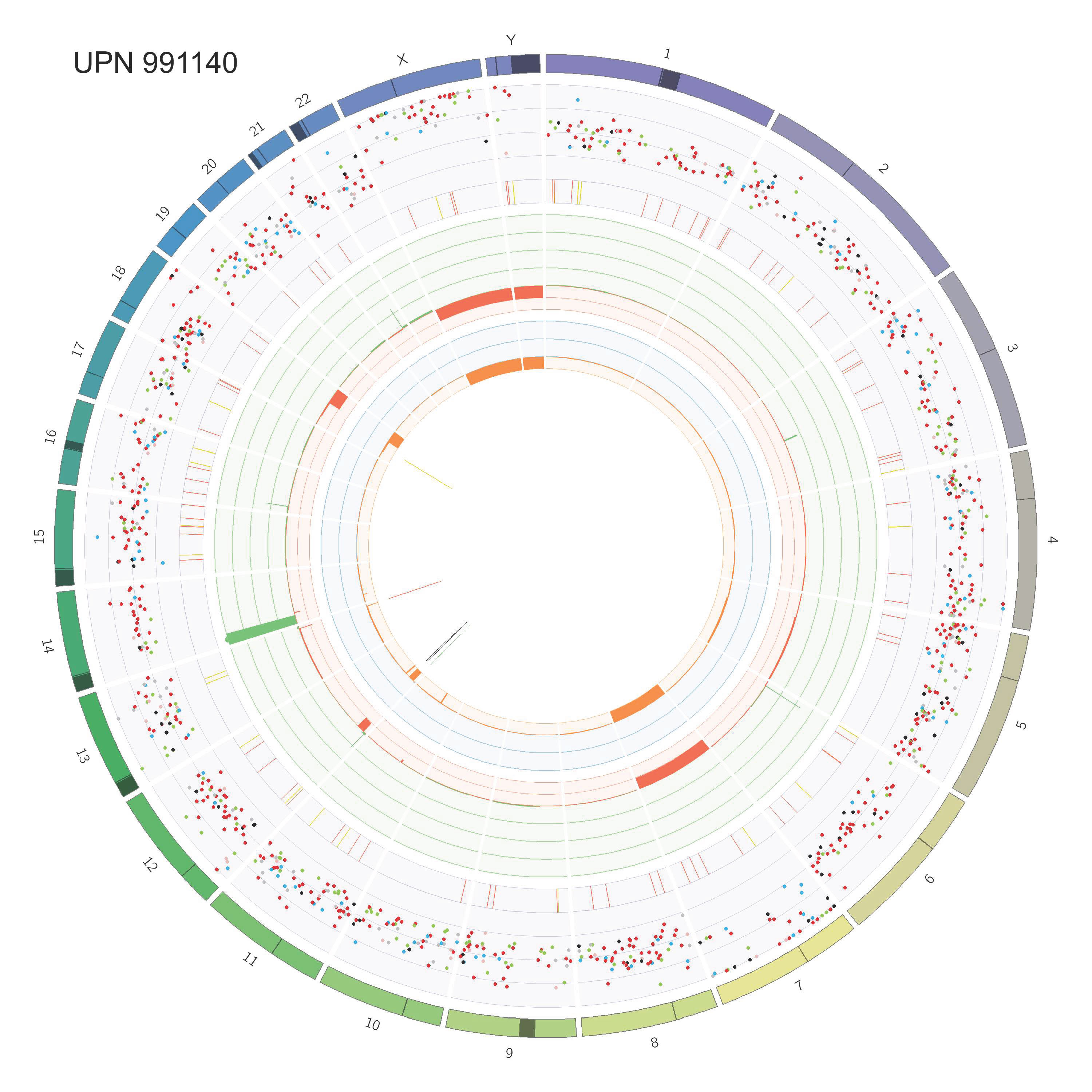

Supplement: Supplement 3 — Supplementary Figure 2. Circos plots [file media-3.zip › Supp_Fig_2_circos_Page_60.jpg]

**A**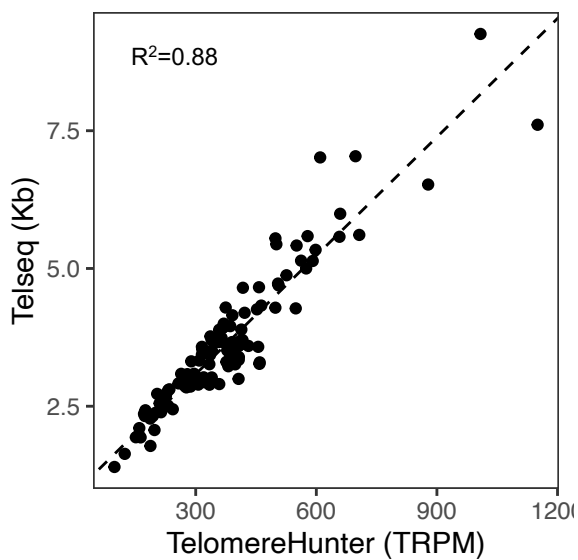**B**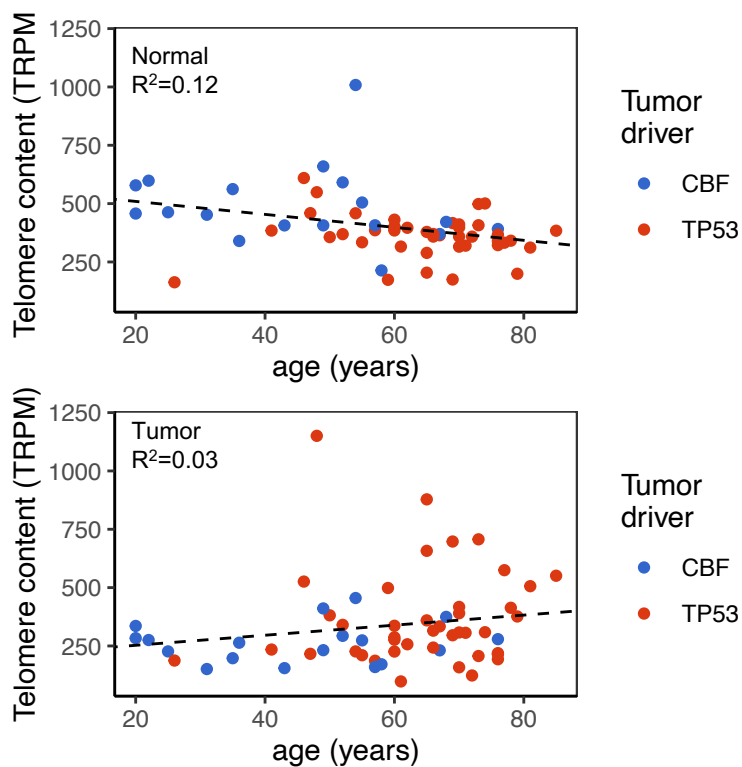

Supplement: Supplement 4 — Supplementary Figure 3. Telomere content agreement between methods. Telomere content by patient age. [file media-4.pdf]

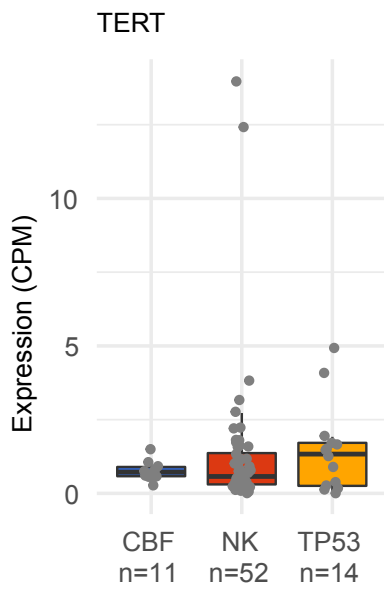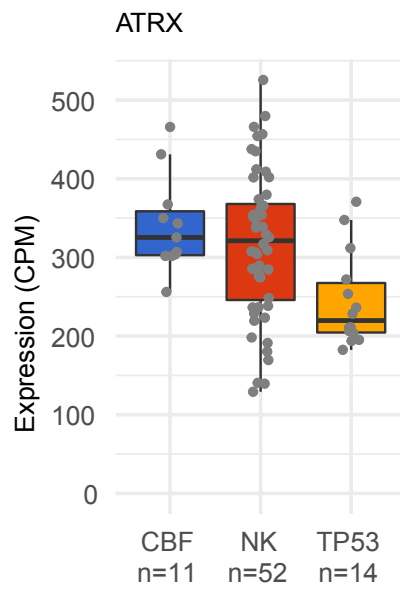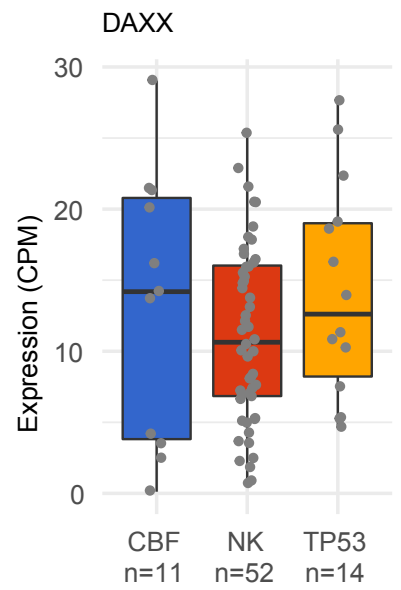

Supplement: Supplement 5 — Supplementary Figure 4. mRNA expression of TERT, ATRX, and DAXX [file media-5.pdf]
